# Battery technology and recycling alone will not save the electric mobility transition from future cobalt shortages

**DOI:** 10.1038/s41467-022-29022-z

**Published:** 2022-03-15

**Authors:** Anqi Zeng, Wu Chen, Kasper Dalgas Rasmussen, Xuehong Zhu, Maren Lundhaug, Daniel B. Müller, Juan Tan, Jakob K. Keiding, Litao Liu, Tao Dai, Anjian Wang, Gang Liu

**Affiliations:** 1 School of Business, Central South University, 410083 Changsha, China; 2 SDU Life Cycle Engineering, Department of Green Technology, University of Southern Denmark, 5230 Odense, Denmark; 3 Institute of Metal Resources Strategy, Central South University, 410083 Changsha, China; 4 Industrial Ecology Programme, Department of Energy and Process Engineering, Norwegian University of Science and Technology, 7491 Trondheim, Norway; 5 Center for Minerals and Materials, Geological Survey of Denmark and Greenland, 1350 Copenhagen, Denmark; 6 Institute of Geographic Sciences and Natural Resources Research, Chinese Academy of Sciences, 100101 Beijing, China; 7 Research Center for Strategy of Global Mineral Resources, Chinese Academy of Geological Sciences and China Geological Survey, 100037 Beijing, China

**Keywords:** Environmental social sciences, Environmental sciences, Energy science and technology

## Abstract

In recent years, increasing attention has been given to the potential supply risks of critical battery materials, such as cobalt, for electric mobility transitions. While battery technology and recycling advancement are two widely acknowledged strategies for addressing such supply risks, the extent to which they will relieve global and regional cobalt demand–supply imbalance remains poorly understood. Here, we address this gap by simulating historical (1998-2019) and future (2020-2050) global cobalt cycles covering both traditional and emerging end uses with regional resolution (China, the U.S., Japan, the EU, and the rest of the world). We show that cobalt-free batteries and recycling progress can indeed significantly alleviate long-term cobalt supply risks. However, the cobalt supply shortage appears inevitable in the short- to medium-term (during 2028-2033), even under the most technologically optimistic scenario. Our results reveal varying cobalt supply security levels by region and indicate the urgency of boosting primary cobalt supply to ensure global e-mobility ambitions.

## Abstract (web summary)

New study finds cobalt-free batteries and recycling progress can significantly alleviate long-term cobalt supply risks, however a cobalt supply shortage appears inevitable in the short- to medium-term, even under the most technologically optimistic scenario.

## Introduction

While renewable energy and low-carbon technology transitions are imperative to achieve the climate neutrality and post-COVID-19 green recovery ambitions of many countries^1,2^, such transitions require various types and significant amounts of critical materials (e.g., rare earth for magnets, platinum for catalysts, and lithium for batteries)^3–7^. In particular, while the decarbonization of the transport sector can benefit from sustainable fuels such as electrofuels and biomethane^8^, battery technology, which depends fundamentally on critical materials such as lithium, cobalt, and nickel, is widely deemed indispensable in renewable energy storage and automobile electrification^9,10^. Both lithium and cobalt are deemed critical materials by major economies such as the U.S.^11^, China^12^, the EU^13^, Japan^14^, and Australia^15^ due to their potential geopolitical supply risks and the importance of the renewable energy transition. Therefore, understanding the demand for such critical materials and exploring mitigation strategies for potential supply risks are essential for ensuring a green and low-carbon future^16,17^.

The global cobalt demand, for example, increased by more than 5 times between 1995 and 2019, and almost half of the global cobalt use in 2019 was for batteries^18^. Such an escalating demand is expected to continue due to the fast diffusion of electric vehicles (EVs) to combat climate and pollution challenges in the coming decades^19^. However, global cobalt mining and refining are very unevenly distributed (e.g., 70% of mine production came from the Democratic Republic of Congo (DRC) and 67% of refining occurred in China in 2019^20,21^), which raises enormous concerns about future demand–supply imbalances among governmental and industry decision-makers.

The two most widely discussed strategies for addressing such supply risks are battery technology development and progress in recycling^22–24^, in addition to further mineral exploration and trade diversification^25^. Indeed, as the price of cobalt has fluctuated (e.g., it tripled from 2016 to 2018) and environmental and social concerns about cobalt mining in the DRC^26^ have increased, the prospect of battery development with less or even no cobalt has gained increasing attention in recent years^27–29^. When more EVs and batteries reach their end of life (EoL), secondary cobalt provision through recycling will be essential to supplement the primary supply^30,31^. There has been a growing body of literature on global and national cobalt material flows^32–38^, trade links^39,40^, demand forecasting^41^, and recycling potential (mostly of lithium-ion batteries)^42–45^. However, the extent to which battery and recycling technology progress will relieve the global and regional cobalt demand–supply imbalance, particularly considering the spatiotemporal variations in different world regions, remains poorly understood.

**Fig. 1:**
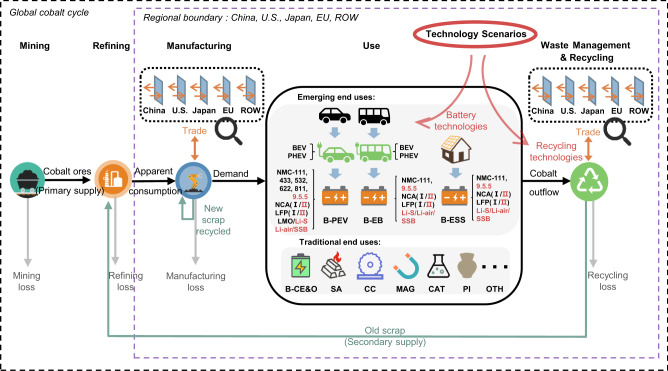
System definition of the global anthropogenic cobalt cycle and modeling framework for cobalt demand and secondary supply potentials. BEV battery electric vehicles, PHEV plug-in hybrid electric vehicles, NMC lithium nickel manganese cobalt oxide, NCA(I) lithium nickel cobalt aluminum oxide, NCA(II) advanced NCA with lower cobalt content and higher energy density, LFP(I) lithium iron phosphate, LFP(II) new form of LFP (e.g., blade LFP battery developed by the company BYD), LMO lithium manganese oxide, Li-air lithium-air, Li-S lithium-sulfur, SSB solid-state battery, B-PEV battery for electric passenger vehicles, B-EB battery for electric buses, B-ESS battery for energy storage systems, B-CE&O battery for consumer electronics and other battery products, SA superalloy, CC cemented carbides, MAG magnets, CAT catalysts, PI pigments, OTH other end uses. Battery cathodes in black and red colors indicate state-of-the-art battery technologies and future battery technologies, respectively.

Here, we aim to answer this question by simulating historical and future global cobalt stocks and flows with regional resolution on major economies (i.e., China, the U.S., Japan, the EU, and the rest of the world (ROW)) based on dynamic material flow analysis (MFA) (see modeling framework in Fig. 1 and details under Methods). Both traditional (e.g., superalloys and magnets) and emerging (e.g., power batteries) end uses of cobalt are considered, while the latter has a higher resolution (e.g., different purposes and battery chemistries) to enable discussion on technological progress. We first characterize the global and regional cobalt cycle from 1998 (when cobalt use started to penetrate the market) to 2019 (the latest data available) and then employ a prospective stock-driven approach^4,46,47^ to explore cobalt demand and secondary supply potential by end use in each region up to 2050. We show that cobalt-free batteries and recycling progress can indeed significantly alleviate cobalt supply risks in the long run; however, a cobalt shortage between 2028 and 2033 appears inevitable, even under the most optimistic scenario, due to global automobile electrification ambitions. Our results reveal significant regional disparities in future cobalt demand-supply balance and supply security levels and indicate the urgency of boosting primary cobalt supply to ensure global e-mobility ambitions.

## Results

### Historical cobalt stocks and flows at global and regional scales

**Fig. 2:**
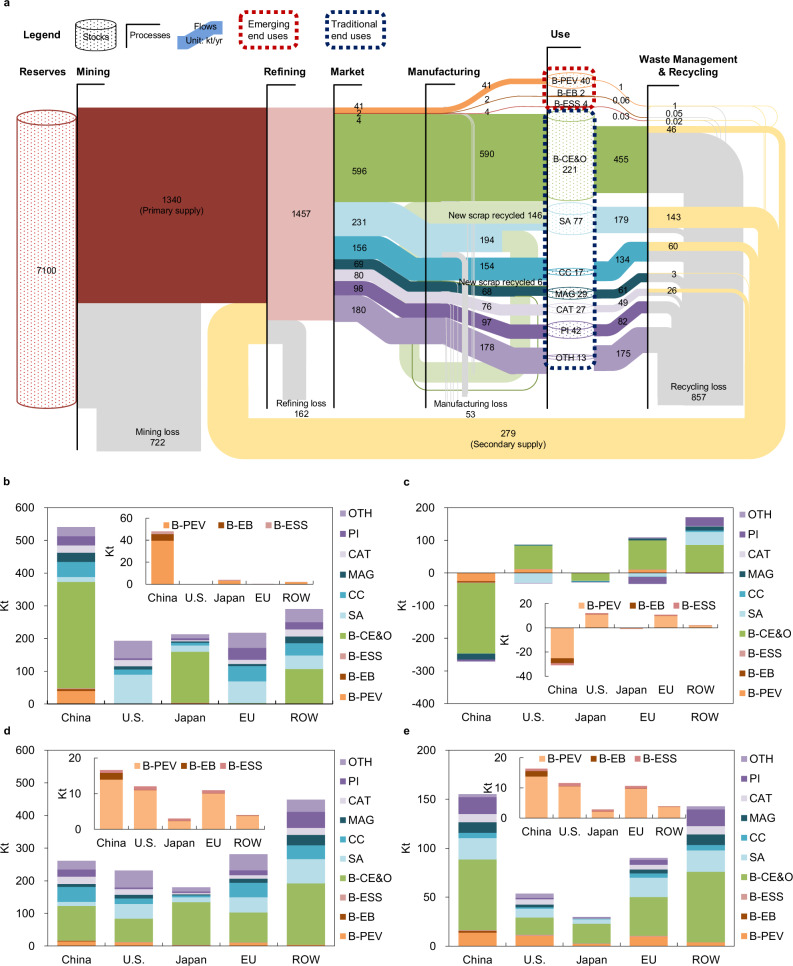
Historical cobalt stocks and flows at global and regional scales, 1998–2019. **a** Cumulative global cobalt cycle, **b** cumulative cobalt apparent consumption (inflows to manufacturing) by sector by region, **c** cumulative net import (positive values) and net export (negative values) of cobalt-containing final products by sector by region, **d** cumulative demand (inflows to in-use stocks) by sector by region, and **e** in-use stocks by end use by region in 2019. All values are in metric kilotons as cobalt metallic equivalent.

The global anthropogenic cobalt cycle (Fig. 1) includes five transformation processes: mining, refining, manufacturing, use, and waste management and recycling. The global cumulative cobalt apparent consumption (flows entering manufacturing processes) amounted to 1455 kt between 1998 and 2019, as shown in Fig. 2a, while owing to manufacturing losses, the global cumulative cobalt demand (inflows into in-use stocks of different end-use categories) added up to 1403 kt during the same period. This demand was mostly for B-CE&O (42%), followed by SA (14%), OTH (13%), and CC (11%). The global in-use stock of cobalt reached 471 kt in 2019, and B-CE&O contributed the largest proportion of this (47%). The cumulative cobalt demand and in-use stock for emerging end uses accounted for a small share (3% and 9%, respectively) because they were still in the early development stage in 2019. Primary production (1340 kt) was still the main source of supply, accounting for 83%. Only 279 kt of old (postconsumer) scrap cobalt was recycled (from mainly SA, CC, and B-CE&O) and reentered the refining process. New scrap that was recycled and reentered the manufacturing process from 1998 to 2019 amounted to 152 kt, but 96% of this scrap was superalloys, due to their high value and purity. The cumulative cobalt losses in the mining, refining, manufacturing, and waste management and recycling stages from 1998 to 2019 were 722, 162, 53, and 857 kt, respectively. Notably, the B-CE&O contributed to 48% of the total cumulative recycling loss due to the low collection rates for EoL consumer electronics.

Among all world regions, China consumed the most refined cobalt (541 kt; in terms of apparent consumption shown in Fig. 2b) from 1998 to 2019. The majority (60%) of this consumption was in B-CE&O manufacturing, which was further exported as semi-finished or final products (216 kt; Fig. 2c) to the other regions. This reflects China’s role as a ‘world factory’ for consumer electronics in this period. Similar to China, Japan also has a large cumulative apparent consumption and a high share of consumption through B-CE&O due to the country’s large electronics industry. In contrast, the U.S. and EU produce only a small amount of B-CE&O and depend mainly on imports for their supply. Instead, most of the cobalt apparent consumption in the U.S. and Europe went into superalloys due to their advanced military and aerospace industry. In terms of cumulative cobalt demand (inflows to in-use stocks from 1998 to 2019, Fig. 2d), the ROW clearly appears to be the highest. China has the highest amount of in-use stocks, reflecting its relatively later development and thus newer stock. When only emerging end-use categories are considered, China shows the largest cobalt cumulative demand (17 kt) and in-use stock (16 kt), notably accounting for 98% of the global cumulative demand and in-use stock of cobalt for B-EB during this period. Among the different types of emerging end uses, B-PEV dominates the cobalt demand, while B-EB and B-ESS have accounted for a small fraction to date.

### Global prospects

In this work, we establish four battery cathode technology (BT) scenarios (state-of-the-art battery development BT1, lower cobalt evolutionary progress BT2, mature cobalt-free technology penetration BT3, and next-generation cobalt-free battery breakthrough BT4; see details in the Methods) with varying market shares based on lithium-ion battery (LIB) technology progress assumptions in terms of cobalt intensity. Based on such BT assumptions and additional key parameters (Table 1) such as battery lifetime and progress in recycling, we have selected and presented seven scenarios (as detailed in Table 2 in the Methods) to discuss global and regional cobalt demand–supply imbalance and resource security that consider varying battery technologies (S1–S4), battery lifetime technologies (S5), recycling progress (S6), and the most ambitious technology combinations (S7).

**Table 1:** Description and assumptions of key model parameters.

Key parameters	Descriptions/Assumptions	Details
Cobalt intensity	The cobalt intensity of different battery cathode materials varies by each type^43,78,79^ and is assumed to remain at the current level in the future.	Supplementary Table 6
Battery cathode material market share	The cathode materials of the state-of-the-art battery cathode technologies are assumed to shift from NMC-111 toward NCA and NMC-811^19,68,75^. The low-cobalt battery cathode technologies (NMC-9.5.5 and advanced NCA)^71,80^, new LFP^10^, and next-generation cobalt-free technologies^28,81^ are assumed to gradually penetrate the market from 2020, 2020, and 2030, respectively, replacing the state-of-the-art technologies, and then further approaching 100% by 2050.	Supplementary Figs. 6–8, Supplementary Table 6
The share of BEV/PHEV in EV sales	PHEV is assumed to transform gradually to BEV, and BEV will dominate the EV market in the future^82^.	Supplementary Figs. 11, 12, Supplementary Table 9
Battery capacity of BEV/PHEV	The battery capacity for PHEV and BEV are both assumed to increase based on the prediction of IEA^82^ that considers regional differences in battery technology roadmaps.	Supplementary Figs. 13, 14, Supplementary Table 9
EV market share	The EV market share is assumed to increase based on the prediction of IEA^82^, which considers regional differences in carbon mitigation ambitions and existing EV policies.	Supplementary Figs. 15, 16, Supplementary Table 9
Vehicle ownership	Vehicle ownership is assumed to grow or level off based on regional historical levels, their potential socioeconomic development, and mobility as a service pattern.	Supplementary Figs. 17, 18, Supplementary Table 9
ESS stock	The global ESS stock is assumed to grow, based on the literature^76,83,84^, with regional proportions set according to per capita renewable electricity generation^85^.	Supplementary Fig. 19, Supplementary Table 9
Battery lifetime	The average lifetimes of B-PEV, B-EB, and B-ESS are assumed to be 8^32^, 7^75^, and 10^76^ years, respectively, in the base scenario. Their lifetimes are assumed to double in the extending battery lifetime scenario^77^ enabled by technology innovation.	Supplementary Table 7
Recycling rate	The recycling rates for each end use are assumed to rise by 10% in 2050 based on the historical levels^32,86^ in the base scenario and to approach 95% by 2050 for all recyclable end uses in the high recycling rate scenario. The recycling rate for each region is assumed the same.	Supplementary Fig. 9, Supplementary Table 8
Cobalt stock per capita for traditional end uses	Assumptions are based on the historical cobalt stock levels and potential technology changes considering regional differences.	Supplementary Table 10
Population	The predicted population per region is based on the World Population Prospects 2019 published by the United Nations Population Division^87^.	Supplementary Table 10
Primary supply	The primary cobalt supply from ore from 2020 to 2030 and afterward is based on previous research^41^ and categorized as two (base and high) scenarios.	Supplementary Fig. 10

The development of emerging end uses (especially B-PEV) would significantly increase the global cobalt demand in the following decades. For example, the cobalt demand for B-PEV would reach 1258 and 591 kt in 2050 for the S1 and S2 scenarios, respectively, corresponding to 79 and 66% of their respective total demand. Compared with state-of-the-art battery cathode technologies (S1), low-cobalt battery cathode technologies (S2) would effectively decrease cobalt demand, and the diffusion of cobalt-free battery cathode technologies (S3 and S4) would totally change the picture. Under the S3 and S4 scenarios with cobalt-free battery technologies, the cobalt demand for B-PEV would peak at 175 kt in 2033 and 612 kt in 2038, respectively, and fall to 6 and 3 kt in 2050, respectively (2% and 1% of their respective totals). The battery cathode technology advancement can also reduce the cobalt demand in B-EB and B-ESS, despite much lower levels due to their smaller market shares. Doubling battery lifetimes (S5) would also reduce cobalt demand by 619 kt by 2050, which would be nearly half the total demand in S1. Cobalt demand for traditional end uses would double from 144 kt in 2020 to 273 kt in 2050 for all scenarios. Traditional end-use sectors would gradually dominate again in the total cobalt demand with the penetration of new battery technologies.

Recycling cobalt as a secondary supply would be an essential way to supplement to primary supply. It would gradually become the major source of cobalt supply as more cobalt-containing products reach their EoL. The increase in the EoL recycling rate for cobalt-containing products would improve the secondary supply by 3680 kt in total from 2020 to 2050 in the S6 scenario. Under the S4 and S7 scenarios, the secondary cobalt supply could exceed the total demand after 2044 and 2043, respectively, indicating a closure of the cobalt cycle in the long run through recycling only.

**Fig. 3:**
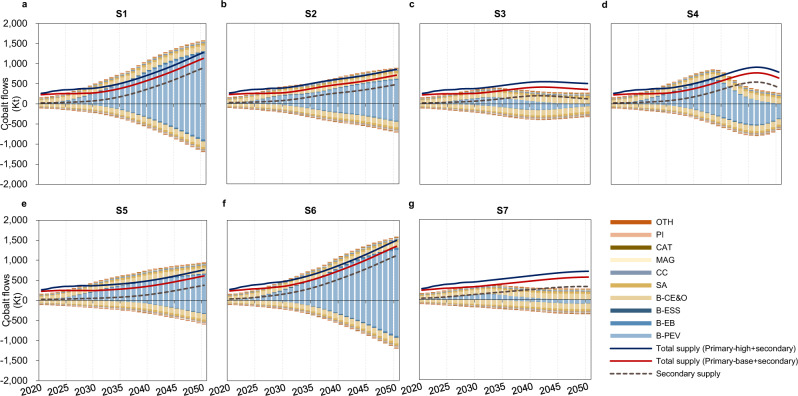
Prospective global cobalt demand (positive values), scrap generation (negative values), and total supply (primary + secondary) under the seven scenarios. **a** S1: state-of-the-art battery cathode technology scenario as the reference scenario; **b** S2: low-cobalt battery cathode technology scenario; **c** S3: LFP-dominant cobalt-free battery cathode technology scenario; **d** S4: next-generation cobalt-free battery cathode technology scenario; **e** S5: extending battery lifetime scenario; **f** S6: high recycling rate scenario; and **g** S7: the most optimistic technology scenario. The scenarios are detailed in Table 2 in the Methods section. The primary-base and primary-high indicate two primary supply scenarios, as shown in Table 1 and Methods.

Primary supply, however, is found to be essential to achieve supply-demand balance in most scenarios. When all the announced or scheduled cobalt production in the operating mines^41^ is considered (primary-base supply scenario), a cobalt supply shortage is still inevitable in all scenarios. Even under the most optimistic scenario (S7), with the most advantageous battery technologies and recycling technologies are employed, a slight supply shortage would still occur from 2028 to 2033. This suggests that battery technology and recycling alone will not save the global ambitious electric mobility transition from cobalt supply shortages if global primary production increases as planned (primary-base supply scenario, the red line in Fig. 3). Only if unscheduled mining projects that have not already been announced^41^ are additionally considered (primary-high supply scenario, the blue line in Fig. 3; detailed in the Methods) can future cobalt supply shortages be prevented under the most technologically optimistic scenarios (S3 and S7 only).

In addition to the abovementioned critical influencing factors, EV market shares and vehicle ownership are the other two key parameters affecting the prospective cobalt demand of power batteries. The sensitivity analysis results of global cobalt demand in total and by end-use sector for all key parameters are shown in Supplementary Figs. 30–39.

### Regional disparities

**Fig. 4:**
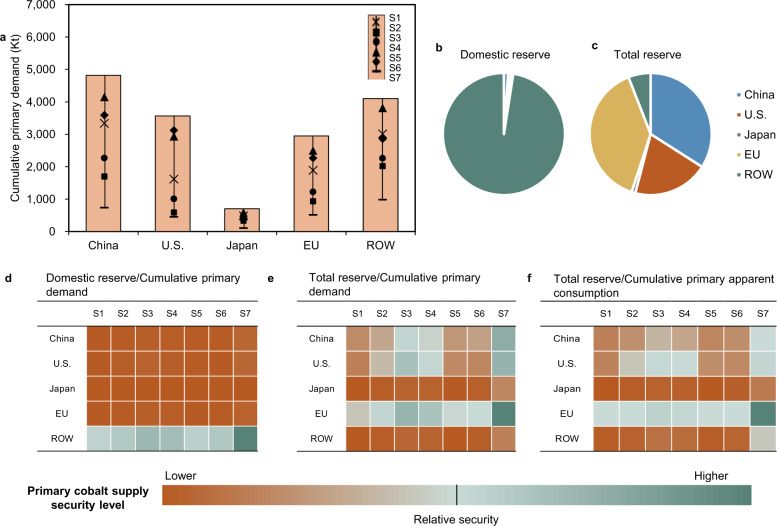
Prospective cumulative primary demand, domestic and total reserve, and corresponding supply security levels under the seven selected scenarios. **a** The regional cumulative primary demand (gross demand minus secondary supply) under the seven scenarios from 2020 to 2050; **b** shares of domestic reserves by region in 2019; **c** shares of total reserves (domestic reserves plus overseas cobalt reserve ownership^88^) by region in 2019 and the primary cobalt supply security level during 2020–2050 measured by **d** domestic reserve divided by cumulative primary demand; **e** total reserve divided by cumulative primary demand; and **f** total reserve divided by cumulative primary apparent consumption (primary demand plus net export of cobalt final products, which are assumed the same as the 2019 levels).

Figure 4 shows the cumulative primary demand (gross demand minus secondary supply) of cobalt in the five world regions under the seven selected scenarios (S1–S7) and their primary cobalt supply security levels (assuming cobalt reserves remain the same as in 2019) measured by different approaches. The results reveal that China has the highest cumulative cobalt primary demand under scenario S1, followed by the ROW, U.S., and EU, while Japan’s cumulative net demand is much lower than that of the other regions (in Fig. 4a). However, the domestic cobalt reserves of China, the U.S., the EU, and Japan are very small, amounting to less than 3% of the global total (7100 kt), as shown in Fig. 4b. Such large gaps between demand and supply imply enormous supply risk for the four major economies.

In reality, of course, these major economies (China, the U.S., Japan, and the EU) have also managed to continuously expand their ownership of foreign cobalt mines through overseas mining projects. Therefore, when the total cobalt reserves (domestic reserves plus foreign cobalt reserve ownership; 2383, 1401, 70, 2732, and 415 kt, respectively, for China, the U.S., Japan, the EU, and the ROW, as shown in Fig. 4c) are considered, the cobalt demand–supply gap would be very different, as shown in Fig. 4d, e. In fact, the EU would no longer face a supply shortage under scenarios S2–S7. In addition, the U.S. and China could avoid supply shortages if the mature or upcoming cobalt-free battery technologies gradually penetrated the market (under scenarios S3, S4, and S7). However, Japan and the ROW would still face supply shortages under all scenarios, even under the most optimistic scenario (S7). The international trade of cobalt-containing products (e.g., final products and scrap) can affect the regional cobalt demand–supply imbalance to some extent. For example, if the future regional trade patterns of cobalt final products remain the same as in 2019 (Fig. 4f), China, as the main exporter of cobalt-containing products (mainly batteries), would no longer be able to secure its cobalt supply, even after the penetration of cobalt-free technologies (LFP dominant in S3 and next generation in S4), whereas the EU would be able to ensure cobalt supply security in all seven scenarios.

## Discussion

Our results reveal that battery technology innovation, especially cobalt-free technologies, can significantly alleviate the cobalt supply risk. Many battery producers have already prioritized lowering the cobalt content in LIBs and producing NMC-811 instead of NMC-111 in the face of expensive cobalt and geopolitical supply risks^24^. However, due to the rapid expansion of the EV market and increasing battery capacity, the cobalt shortage appears inevitable in the future if the primary supply follows only the scheduled extraction plan as announced for each deposit^41^ (primary-base supply scenario). Low-cobalt battery cathode technology development could alleviate, but not prevent, the supply crisis. This demand–supply gap would still occur around 2028–2033, even though cobalt-free LFP technology already penetrated the market in 2020 and it is predicted that the next-generation cobalt-free battery technologies will become commercialized by 2030. Nevertheless, current (LFP) and upcoming (Li-air, Li-S, and SSB) cobalt-free technology penetration could still reduce cobalt cumulative demand by 62% and 41%, respectively (see Fig. 3c). Extending the battery lifetime with technology innovation is another effective measure to mitigate cobalt supply pressure. Our results show that doubling the battery lifetime would nearly halve the cobalt demand. Therefore, battery technology is crucial to mitigate potential cobalt shortages, and joint efforts are urgently needed to further accelerate the research and market penetration of various decobaltization battery technologies, particularly cobalt-free technologies. In view of the short- to medium-term (during 2028–2033) shortage of cobalt supply, further accelerated penetration of the new LFP battery technology might be the most effective cure. However, due to the significant barrier of the low energy density of the LFP cathode, next-generation cobalt-free technologies are still indispensable in making more powerful batteries and alleviating mileage anxiety in PEVs, which requires governmental financial and regulatory support and multistakeholder cooperation, including that between industries and research institutes.

Cobalt recycling is a vital strategy for supplementing the primary supply. The increasing secondary cobalt availability under various scenarios suggests considerable potential for recycling to relieve primary resource pressure in the future, especially in the long term. For example, secondary cobalt accounts for 66% of the accumulative cobalt demand under scenario S7, 75% of which would be generated from 2035 to 2050. Therefore, combined efforts in technology, regulatory, and economic areas should be explored to motivate cobalt recycling^48^ so as to ensure cobalt supply security in the future. First, further technological development for the recycling of products with currently low recycling rates would be essential to harness the increasing potential of urban mining. For example, the present recycling rate of B-CE&O is only 10% due to high cost and technological challenges, which can be mitigated by further development and the application of leaching-regeneration hydrometallurgy^49^. Second, an extended producer responsibility system and design for remanufacturing, reuse, and recycling should be further promoted to stimulate closed-loop recycling among manufacturers, especially for cars and battery producers, due to their significant role in future cobalt consumption. Third, a better societal collection and recycling system should be constructed to reduce the EoL losses, taking advantage of emerging information technologies such as internet Plus, integrated logistics networks, and the big data platform^36^ to connect consumers with qualified recyclers, logistics companies, processing companies, and producers and to promote online and offline interactions. Finally, relevant regulations and industrial standards should be established to attract more investment in the recycling industry, and policy support should be provided to enterprises, particularly small and medium-sized enterprises, to ensure sustained profitability when facing the increasing financial costs of technology advancement and equipment upgrading^50^.

Primary supply is necessary and important for ensuring future cobalt demand–supply balance, especially in the short- to medium-term. Our scenario results show that increasing primary supply is urgently needed to close the cobalt demand–supply gap that cannot be fully filled by battery technology and recycling progress alone. A primary supply increase could be realized via more exploration, investment, and technology development for both existing and unexplored cobalt deposits. Therefore, enhancing the exploration of cobalt deposits, deep-sea mining enabled by advanced extraction technologies, and improving the efficiency of ore extraction, smelting, and refining could all further boost the primary supply. For example, the future prevalence of high-pressure acid leaching in the EU would improve cobalt extraction from laterite deposits^25^, and the reopening of Mutanda would significantly increase cobalt mine production^51^.

Despite the significance of primary supply, a few key uncertainties and challenges in increasing primary cobalt supply remain. First, cobalt ore production is economically susceptible to short-term price fluctuations and rising production costs^52^. For example, the international mining giant Glencore, which is the world’s top cobalt mine producer, had to close the Mutanda copper-cobalt mine in the DRC, which provided 1/5 of the global cobalt production in 2018 due to the increasing input costs and taxes as well as the continually decreasing price^52^. Second, it typically takes over a decade from the discovery of undiscovered deposits to the extraction of the first ore, and such a time lag makes it difficult to address the global and regional demand–supply imbalance in the short- to medium-term. Third, 94% of cobalt production is accompanied by copper or nickel ores as byproducts^53^, so cobalt is closely related to the production and price fluctuations of these two metals. Fourth, given by the massive extraction of specific cobalt deposits, the decreasing ore grade of cobalt is inevitable, which would increase cobalt production costs (economically, socially, and environmentally) and affect cobalt production to some extent. Last but not least, the cobalt primary supply faces various types of geopolitical (e.g., civil wars and unstable governmental systems in producing countries) and supply chain risks, as exemplified by the on-going COVID-19 pandemic-induced lockdown in the DRC and corresponding impacts on shipping ports (e.g., Durban in South Africa) and production capacities^54^.

Given the regional resolution of our data, our results can shed light on regional resource security concerns and mitigation strategies. China is expected to consume much more cobalt than any other region in the future, primarily due to its enormously booming domestic EV market and expanding battery production capacity in the future. In 2019, China’s cobalt reserve was only 80 kt, but it contributed to 67% of the global cobalt refinery production^55^, which resulted in its strong dependence on overseas primary cobalt resources. Although we show that China could secure its cobalt demand–supply balance under the most optimistic scenario (Fig. 4f), its supply risk will still be considerably high if the country continues to expand exports of batteries or cannot accelerate the market penetration of cobalt-free technologies. Reducing the cobalt supply risk thus requires both the diversification of secured import channels of primary cobalt and system planning for industry policy, urban mining, and battery technology innovation.

The cobalt supply security levels for the EU and U.S. are relatively high. They have, on the one hand, transferred part of the supply pressure of primary cobalt to China and Japan by outsourcing battery production there and, on the other hand, controlled most of overseas cobalt mine production by their mining giants. However, this may change as they plan to build a stronger domestic battery industry to reduce supply chain risks and boost domestic industrial competition and employment (e.g., as planned in the EU’s Battery 2030 + Strategy^56^) and as competition for overseas mining projects becomes fiercer in the future. Targeted initiatives have already been implemented by some companies to address the vulnerable cobalt supply chain. For example, Apple now attempts to procure cobalt directly from miners^57^, and Tesla’s battery suppliers have been expanded from only Panasonic to multiple Japanese and Chinese battery producers^58^. The cooperation between manufacturers and their suppliers regarding technology development and market information should be encouraged to make the supply chain more transparent and robust. For example, Tesla has already begun to partially replace their NCA batteries with new LFP technologies in some EV models that have been put on the Chinese market by collaborating with the local battery supplier CATL^59^.

Although the cobalt demand for Japan is much lower than that for other regions, the limited domestic cobalt reserves and overseas reserve ownership lead to a relatively higher supply risk in Japan. In this regard, Japan’s ambition to build a ‘hydrogen society’, which signifies a preference for fuel cell vehicles (cobalt-free vehicles) in the transportation system instead of BEVs and PHEVs, could significantly reduce the country’s dependency on cobalt and mitigate potential supply shortages to some extent.

Our study primarily investigated the extent to which battery and recycling technology progress can relieve the global and regional cobalt demand–supply imbalance in the coming decades. Due to data gaps, the absolute results should be interpreted with caution and with unavoidable uncertainties in mind. First, only PEVs and EBs are included for the electric mobility transition, while HEVs, electric two-wheelers, electric bicycles, and electric trucks are not explicitly considered. Second, the battery lifetime is set as half or the same as the PEV lifetime; this indicator could be further improved by considering battery health status and real-time modeling in the future. Third, EoL management and circular economy strategies other than recycling, such as remanufacturing and reuse for grid stability or private energy storage, need to be further discussed in the future. Nevertheless, we believe our scenario results still provide robust conclusions about the characteristics of global and regional historical and future cobalt cycles and the role of technology innovations in addressing the demand–supply imbalance. The identified inevitable supply shortage in the short- to medium-term calls for multistakeholder, beyond-technology, and urgent actions and joint efforts to increase primary supply and boost technology innovations for securing a green transition in the future.

## Methods

### System definition

The global anthropogenic cobalt cycle (as shown in Fig. 1) includes five transformation processes: mining, refining, manufacturing, use, and waste management & recycling. Cobalt flows into the refining process can be derived from three sources: cobalt ores produced from mining (primary supply), cobalt old scrap recycled from EoL products (secondary supply), and the net import of cobalt old scrap. The cobalt-containing final products can be classified into three emerging end uses and seven traditional end uses. Emerging end uses include batteries for passenger electric vehicles (B-PEVs), batteries for electric buses (B-EBs), and batteries for energy storage systems (B-ESSs). Traditional end uses include batteries for consumer electronics and other battery products (B-CE&O), superalloys (SA), cemented carbides (CC), magnets (MAG), catalysts (CAT), pigments (PI), and other uses (OTH). Losses were considered in mining, refining, manufacturing, and waste management and recycling processes; part of the new scrap generated in manufacturing can be efficiently recycled in corresponding manufacturing sectors. The global cobalt cycle and relevant stocks and flows are disaggregated into five regions: China, the U.S., Japan, the EU, and the ROW. The international trade of cobalt-containing final products and old scrap between these five regions are also taken into consideration.

### Historical cobalt cycle and stocks and flows

A dynamic MFA approach^4,60^ was employed to quantify the global cobalt cycle from 1998 to 2019. For example, the outflows were calculated as shown below:

1 $${outflow}\left(t\right)=\int _{{t}_{0}}^{t}{inflow}\left(\tau \right)* \left(\frac{1}{\sigma * \sqrt{2* \pi }}* {{e}^{-\frac{(\tau \,-\,\mu )}{{2\sigma }^{2}}}}^{2}\right)$$

where $\left(\frac{1}{\sigma \,*\, \sqrt{2\,*\, \pi }}* {{e}^{-\frac{(\tau \,-\,\mu )}{{2\sigma }^{2}}}}^{2}\right)$ represents the lifetime distribution (assumed to be the normal distribution) of cobalt-containing end-use products, *t* is the actual time,*τ* is the age cohort (time of input), *μ* is the average lifetime of the products, and *σ* is the standard deviation.

The cobalt stocks in year t were determined by the initial stock and annual net stocks that equal inflow minus outflow over the past t year. The initial cobalt in-use stocks of each end use before 1998 were assumed to be negligible. Cobalt inflows data were compiled from a wide range of statistics and literature. Losses were determined by relative coefficients of the corresponding process. Other flows are calculated by the mass balance principle. The indirect trade of cobalt embodied in final products in international trade was also accounted for since the regional cobalt apparent consumption data by end uses did not include that part. We have detailed the quantification of historical cobalt stocks and flows from 1998 to 2019 in Supplementary Table 2.

### Prospective cobalt cycle and stocks and flows

Stock-driven dynamic MFA models^61–63^ were employed to simulate prospective cobalt demand by end use by region. Seven traditional end uses and three emerging end uses were considered for all five regions and the global total, but the simulation processes varied between them.

The prospective cobalt demand for traditional end uses was determined by the lifetime distribution function and future stock patterns derived from population prospects and per capita cobalt stock assumptions. The per capita cobalt stock of each traditional end use was simulated using a logistic model^64,65^ that complies with the S-shaped curve based on the historical patterns^4,47,62^ and considers the possible impact of technological changes (as shown in Eq. (2)).

2 $$S\left(t\right)=\frac{K}{1+\left(\frac{K}{{S}_{0}}-1\right)* {e}^{-a\,*\, (t\,-\,{t}_{0})}}$$

where *S(t)* is the stock at time *t*, *K* is the assumed stock saturation level, *S*_*0*_ is the initial stock, and *a* is a constant.

The prospective cobalt demand for emerging end uses was based on a product-specific stock-driven model that consists of the fleet module and the material module. The prospective cobalt demand for B-PEV and B-EB was determined by the cobalt intensity, battery cathode material market shares, average battery capacity per vehicle, and battery demand for EVs. The cobalt demand for B-ESS was affected by cobalt intensity, battery cathode material market shares, and future assumed ESS demand.

### Battery cathode technology scenarios

We set four BT scenarios with varying market shares based on the assumed technological progress in terms of cobalt intensity: state-of-the-art battery development, cobalt-less evolutionary progress, new LFP development with zero cobalt, and next-generation cobalt-free revolutionary breakthrough, as summarized below and detailed in Supplementary Figs. 6–8.

- BT1 assumes that the ‘state-of-the-art’ LIB technology and its foreseeable inventions in the near future (i.e., expected shift from NMC-111, NMC-433, NMC-532, and NMC-622 to NMC-811 with less cobalt intensive chemistry^66,67^) will remain until 2050.
- BT2 considers evolutionary progress in LIB technology with the penetration of ‘low-cobalt batteries’, such as NMC-9.5.5^68–70^ and advanced NCA^71^, with an average cobalt intensity of 0.05 kg/kWh^30^ from 2020. State-of-the-art battery cathode technology will accordingly be substituted from 2020 onward in BT2.
- BT3 assumes that the relatively mature cobalt-free battery technology LFP with a higher energy density than the former generation (e.g., blade battery developed by BYD company) will gradually replace NMC and NCA from 2020 onward and will dominate the market completely by 2050^10^.
- BT4 includes a revolutionary breakthrough with ‘cobalt-free battery cathode technology’^72^, such as lithium-air, lithium-sulfur^73^, and solid-state batteries^74^. We assume that next-generation cobalt-free battery cathode technologies will start penetrating and substituting state-of-the-art battery technologies in 2030^45^.

### Battery lifetime scenarios

Battery lifetime is an important parameter for determining cobalt inflows and outflows. We set two scenarios for battery lifetime to explore the impact of battery lifetime extension: one as the current value (8^32^, 7^75^, and 10^76^ years, respectively, for B-PEV, B-EB, and B-ESS), and the other doubling the present level as a result of improved battery technologies (e.g., electrolyte technology)^77^.

### Recycling scenarios

Two recycling progress scenarios were used to quantify the potential secondary cobalt supply: one assumed that the present EoL recycling rates increase by 10% in 2050 based on historical levels, and the other assumed that these recycling rates increase gradually to a high level of 95% by 2050 following an S-shaped curve (except for some end uses that cannot be recycled; see details in Supplementary Fig. 9).

### Primary supply scenarios

Two primary supply scenarios (one called primary-base and the other one called primary-high) are assumed accordingly based on the literature^41^. The primary-base supply scenario considered only announced or scheduled cobalt mine production until 2030. The primary-high supply scenario considered both the scheduled and unscheduled cobalt mine production until 2030. The primary supply after 2030 was assumed to increase with an annual growth rate of 1% for both scenarios (see details in Supplementary Fig. 10).

### Description of the seven selected scenarios

Based on the scenario set for the key parameters, we selected and presented seven scenarios (as shown in Table 2) to reveal the impact of various technologies (e.g., battery cathode technology, battery lifetime, and recycling technology) on cobalt demand and supply. S1 is the base scenario for presenting the possible situation under state-of-the-art battery cathode technology, current battery lifetime, and recycling technology. S2, S3, and S4 are scenarios in which there is a shift to BT2, BT3, and BT4 batteries. S5 considers extending the battery lifetime. S6 considers a high cobalt EoL recycling rate. S7 is the optimistic scenario with the lowest demand and highest supply under the most advantageous technology assumptions. More details regarding the parameters in Table 2 can be found in Supplementary Tables 6–8 and Supplementary Figs. 6–10.

**Table 2:** Description of the seven selected scenarios.

Scenarios	S1	S2	S3	S4	S5	S6	S7
Descriptions/Assumptions	The EV market expands with the state-of-the-art battery cathode technology, with no changes in battery lifetime and recycling rate	High nickel battery technology with lower cobalt intensity penetrates the market from 2020	New LFP battery technology with zero cobalt penetrates the market from 2020	Next-generation cobalt-free battery technology penetrates the market from 2030	Extending the battery lifetime through improved electrolyte technology	Improving the recycling technology of EoL products	The most optimistic scenario with the lowest demand, highest supply, and the most advantageous technologies
End-use categories	Emerging end uses	Emerging end uses	Emerging end uses	Emerging end uses	Emerging end uses	All end uses	All end uses
Battery cathode market shares	NMC-811 and NCA gradually dominate (100% by 2050), varying among the five regions	NMC-9.5.5/advanced NCA gradually dominate (100% by 2050)	New LFP gradually dominate (100% by 2050)	Li-S/Li-Air/SSB gradually dominate (100% by 2050)	NMC-811 and NCA gradually dominates (100% by 2050), varied among the five regions	NMC-811 and NCA gradually dominates (100% by 2050), varied among the five regions	New LFP gradually dominate (100% by 2050)
Battery lifetime (years)	B-PEV: 8 B-EB: 7 B-ESS: 10	B-PE V: 8 B-EB: 7 B-ESS: 10	B-PEV: 8 B-EB: 7 B-ESS: 10	B-PEV: 8 B-EB: 7 B-ESS: 10	B-PEV: 16 B-EB: 14 B-ESS: 20	B-PEV: 8 B-EB: 7 B-ESS: 10	B-PEV: 16 B-EB: 14 B-ESS: 20
EoL recycling rate	Gradually rising by 10% for all recyclable end uses	Gradually rising by 10% for all recyclable end uses	Gradually rising by 10% for all recyclable end uses	Gradually rising by 10% for all recyclable end uses	Gradually rising by 10% for all recyclable end uses	Reaching 95% for all recyclable end uses by 2050	Reaching 95% for all recyclable end uses by 2050

## Supplementary information

SUPPLEMENTARY INFO

## Data Availability

All data analyzed in this study are included in its Supplementary information files.

## Code availability

The mathematical algorithm used in this study is available from the corresponding author upon reasonable request.
