# Supplementary material for: Battery technology and recycling alone will not save the electric mobility transition from future cobalt shortages
Source: Nat Commun. 2022 Mar 15;13:1341. doi: 10.1038/s41467-022-29022-z (PMC8924274; doi:10.1038/s41467-022-29022-z)
Supplement: Supplementary file 1 — SUPPLEMENTARY INFO [file 41467_2022_29022_MOESM1_ESM.pdf]

Supporting Information for

Battery technology and recycling alone will not save the electric mobility transition from future cobalt shortages

Anqi Zeng[1,2,3#], Wu Chen[2,#], Kasper Dalgas Rasmussen[2], Xuehong Zhu[1,3★], Maren Lundhaug[4], Daniel B. Müller[4], Juan Tan[5], Jakob K. Keiding[5], Litao Liu[6], Tao Dai[7★], Anjian Wang[7], Gang Liu[2,6★]

1. School of Business, Central South University, 410083 Changsha, China

2. SDU Life Cycle Engineering, Department of Green Technology, University of Southern Denmark, 5230 Odense, Denmark

3. Institute of Metal Resources Strategy, Central South University, 410083 Changsha, China

4. Industrial Ecology Programme, Department of Energy and Process Engineering, Norwegian University of Science and Technology, 7491 Trondheim, Norway

5. Center for Minerals and Materials, Geological Survey of Denmark and Greenland, 1350 Copenhagen, Denmark

6. Institute of Geographic Sciences and Natural Resources Research, Chinese Academy of Sciences, 100101 Beijing, China

7. Research Center for Strategy of Global Mineral Resources, Chinese Academy of Geological Sciences and China Geological Survey, 100037 Beijing, China

[#] Equal contribution

[★] Corresponding author: zhuxh@csu.edu.cn; daitao@cags.ac.cn; gli@igt.sdu.dk

Number of pages: 69

Number of tables: 10

Number of figures: 40

Number of notes: 3

Acronyms and abbreviations

EV	Electric vehicle
PV	Passenger vehicle
DRC	Democratic Republic of Congo
MFA	Material flow analysis
the U.S.	the United States
the EU	the European Union
ROW	Rest of World
BEV	Battery electric vehicle
PHEV	Plug-in hybrid electric vehicle
EoL	End of life
EoL-RR	End of life recycling rate
NMC	Lithium Nickel Manganese Cobalt Oxide
NCA	Lithium Nickel Cobalt Aluminum Oxide
LFP	Lithium Iron Phosphate
LMO	Lithium manganese oxide
LIB	Lithium-ion battery
B-PEV	Battery for passenger electric vehicles
B-EB	Battery for electric buses
B-ESS	Battery for energy storage systems
B-CE&O	Battery for consumer electronics and other battery products
SA	Superalloys
CC	Cemented Carbides
MAG	Magnets
CAT	Catalysts
PI	Pigments
OTH	Other end uses
NI	Net import
BT	Battery cathode technology scenario
HEV	Hybrid electric vehicle
NiCd	Nickel cadmium battery
NiMH	Nickel metal hydride battery
HSS	High-speed steel
Li-S	Lithium-Sulphur
Li-air	Lithium-Air
SSB	Solid-state batteries
JOGMEC	Japan Oil, Gas and Metals National Corporation
CNIA	China Non-Ferrous Metals Industry Association
USGS	United States Geological Survey
CI	Cobalt intensity
MSCM	Battery cathode material market share
ABC	Average battery capacity
EVBD	Battery demand for EV
VD	Vehicle demand
ESSD	ESS demand
SPC	Cobalt stock per capita
P	Population

ES	ESS stock
VO	Vehicle ownership
MS-EV	Market share of electric vehicles
L	Battery lifetime

Supplementary Table 1 Commodity list of cobalt-containing final products and waste and corresponding cobalt content.

End uses	No.	HS code (2017)	Commodity name	Co content	Uncertainty
Batteries	1	870240	Vehicles; public transport type; BEV[a]	0.03%	medium
	2	870360	Vehicles; passenger cars with both spark-ignition internal combustion reciprocating piston engine and electric motor for propulsion; PHEV[b]	0.29%	medium
	3	870370	Vehicles; passenger cars with both compression-ignition internal combustion piston engine and electric motor for propulsion; PHEV[b]	0.29%	medium
	4	870380	Vehicles; passenger cars; BEV[b]	0.69%	medium
	5	850750	Electric accumulators; nickel-metal hydride[c]	3%	high
	6	850760	Electric accumulators; lithium-ion[d]	6.85%	high
	7	847130	Automatic data processing machines; portable, weight<10kg[e]	0.85% /1.28%	high
	8	847141	Automatic data processing machines; comprising in the same housing at least a central processing unit and an input and output unit[e]	0.06%	medium
	9	850730	Electric accumulators; nickel-cadmium[d]	0.60%	medium
	10	851020	Hair clippers; with a self-contained electric motor[f]	0.03%	medium
	11	851030	Hair-removing appliances; with self-contained electric motors[f]	0.03%	medium
	12	851010	Shavers; with self-contained electric motors[f]	0.04%	medium
	13	851711	Line telephone sets with cordless handsets[g]	0.02%	medium
	14	851712	Telephones for cellular networks or for other wireless networks[h]	1.44% /2.88%	high
	15	852580	Television cameras, digital cameras, and video camera recorders[i]	1.87%	medium
Super alloys	16	840710	Engines; for aircraft, spark-ignition reciprocating or rotary internal combustion piston engines[j]	2.75%	medium
	17	840910	Engines; parts of aircraft engines (spark-ignition reciprocating or rotary internal combustion piston engines)[j]	2.75%	medium
	18	841111	Turbo-jets; of a thrust not exceeding 25kN[k]	7.86%	high
	19	841112	Turbo-jets; of a thrust exceeding 25kN[k]	5.50%	high
	20	841121	Turbo-propellers; of a power not exceeding 1100kW[l]	7.86%	high
	21	841122	Turbo-propellers; of a power exceeding 1100kW[l]	5.50%	high
	22	841181	Turbines; gas-turbines (excluding turbo-jets and turbo-propellers), of a power not exceeding 5000kW[m]	11.00%	high
	23	841182	Turbines; gas-turbines (excluding turbo-jets and turbo-propellers), of a power exceeding 5000kW[m]	3.67%	high
	24	841191	Turbines; parts of turbo-jets and turbo-propellers[m]	0.47%	high
	25	841199	Turbines; parts of gas turbines (excluding turbo-jets and turbo-propellers)[m]	0.44%	high
	26	880211	Helicopters; of an unladen weight not exceeding 2000kg[n]	3.67%	high
	27	880212	Helicopters; of an unladen weight exceeding 2000kg[n]	1.83%	high
	28	880220	Airplanes and other aircraft; of an unladen weight not exceeding 2000kg[n]	7.33%	high
	29	880230	Airplanes and other aircraft; of an unladen weight exceeding 2000kg but not exceeding 15,000kg[n]	1.10%	high
	30	880240	Airplanes and other aircraft; of an unladen weight exceeding 15,000kg[n]	0.65%	high

	31	880260	Spacecraft; (including satellites) and suborbital and spacecraft launch vehicles[o]	0.13%	high
Cemented carbides[p]	32	845921	Machine-tools; for drilling, numerically controlled	0.15%	medium
	33	845929	Machine-tools; for drilling, other than numerically controlled	0.15%	medium
	34	845931	Machine-tools; for boring-milling, numerically controlled	0.15%	medium
	35	845939	Machine-tools; for boring-milling, other than numerically controlled	0.15%	medium
	36	845941	Machine-tools; for boring, numerically controlled boring machines	0.15%	medium
	37	845949	Machine-tools; for boring, not numerically controlled boring machines	0.15%	medium
	38	845951	Machine-tools; for milling, knee-type, numerically controlled	0.15%	medium
	39	845959	Machine-tools; for milling, knee-type, other than numerically controlled	0.15%	medium
	40	845961	Machine-tools; for milling, (not knee-type), numerically controlled	0.15%	medium
	41	845969	Machine-tools; for milling, not knee-type, other than numerically controlled	0.15%	medium
	42	845970	Machine-tools; for threading or tapping	0.15%	medium
	43	846024	Machine-tools; grinding machines (excluding flat-surface, cylindrical and centreless), numerically controlled	0.15%	medium
	44	846029	Machine-tools; grinding machines (excluding flat-surface), other than numerically controlled	0.15%	medium
	45	846031	Machine-tools; sharpening (tool or cutter grinding) machines, numerically controlled	0.15%	medium
	46	846039	Machine-tools; sharpening (tool or cutter grinding) machines, other than numerically controlled	0.15%	medium
	47	846040	Machine-tools; for honing or lapping	0.15%	medium
	48	846120	Machine-tools; shaping or slotting machines	0.15%	medium
	49	846130	Machine-tools; broaching machines	0.15%	medium
	50	846140	Machine-tools; gear cutting, gear grinding or gear finishing machines	0.15%	medium
	51	846150	Machine-tools; sawing or cutting-off machines	0.15%	medium
Magnets[q]	52	847190	Magnetic or optical readers, machines for transcribing data onto data media in coded form and machines for processing such data	0.03%	medium
	53	850110	Electric motors of an output not exceeding 37.5 W	0.02%	medium
	54	850511	Magnets; permanent magnets and articles intended to become permanent magnets after magnetization, of metal	0.90%	low
	55	847170	Units of automatic data processing machines; storage units	0.03%	medium
	56	851821	Loudspeakers; single, mounted in their enclosures	0.05%	medium
	57	851822	Loudspeakers; multiple, mounted in the same enclosure	0.05%	medium
	58	851829	Loudspeakers; not mounted in their enclosures	0.05%	medium
Catalysts[r]	59	381511	Catalysts, supported; reaction initiators, reaction accelerators and catalytic preparations, with nickel or nickel compounds as the active substance	0.45%	high
Pigments and enamels[s]	60	320710	Pigments; prepared pigments, opacifiers, colors and similar preparations	0.25%	high
	61	320720	Enamels and glazes	0.35%	high
	62	320740	Glass; glass frit and other glass, in the form of powder, granules or flakes	0.10%	high
Others	63	321100	Driers; prepared[t]	0.02%	medium

	64	902121	Dental fittings; artificial teeth[u]	5.00%	medium
	65	902131	Artificial parts of the body[u]	5.00%	medium
	66	902139	Artificial parts of the body; excluding artificial joints[u]	5.00%	medium
Waste	67	810530	Cobalt; waste and scrap[v]	68%	medium

Note: Cobalt content is obtained from various sources (e.g., cobalt industry, expert consultation, and literature) as detailed below the table. Uncertainty level (low-10%, medium-30%, high-50%) is assumed depending on the data source's reliability and relevance.

[a] For electric buses, the average energy capacity of BEV is 81.1 kwh/product[1]. The energy capacity of NiMH is assumed as 100wh/kg by mass of 30g which contains 0.9g Co[1], so it can be calculated that 1 kwh NiMH battery requires 0.3kg Co. It is assumed that all batteries used by BEV bus are lithium-ion batteries, which are composed of 13% NMC, 84% LFP and 3% LMO[2] and the average weight of electric buses are 15000kg.

[b] The battery mass of PHEV and BEV of passenger cars are set 88.9kg and 210kg respectively[3]. It is assumed that current PHEV and BEV only use Li-ion batteries[1]. The market shares of different LIBs cathode materials in passenger electric vehicles are 59% (NMC) and LFP (41%). Assuming the average weight of passenger vehicles are 1500kg.

[c] Assuming 0.90 grams Co per NiMH battery (weigh 30g on average) and 0.6% by mass for NiCd batteries[4].

[d] Generally, the energy capacity of lithium-ion batteries with LCO, NMC and NCA cathode materials are 150-240wh/kg, 200-300wh/kg and 150-220wh/kg respectively[5], which assumed to be 200wh/kg, 250wh/kg and 200wh/kg respectively. 1 kwh LCO cathode contains 0.959kg Co, 1 kwh NCA cathode contains 0.143kg Co, and 0.48kg Co is contained in 1 kwh NMC cathode on average[6,7]. We could obtain that producing 1g LCO LIB should consume 0.192g Co, 1g NCA LIB 0.036g cobalt and 1g NMC LIB 0.096g Co. In 2016, the market shares of 5 different types of cathod materials used in LIBs are 21% (LCO), 26% (NMC), 9% (NCA), 8% (LMO) and 36% (LFP) respectively[8].

[e] Considering the trend of getting thinner and lighter for laptops in recent years, the average weight of laptops is assumed 4.5kg during 1998-2011 and 3kg from 2012 to 2019, and for computers, the average weight is regarded as 32kg[9]. It is assumed that all computers and laptops use lithium-ion batteries with LCO cathode by the weight of 100g and 200g respectively[4].

[f] It is assumed that all shavers, hair clippers and hair-removing appliances use NiCd batteries and a battery weigh a mass of 30 grams[4]. Assuming the average weight of electric shavers are 450g and hair clippers are 550g.

[g] It is assumed that 75% of cordless phones use NiCd batteries and 25% use NiMH batteries, both by mass of 30 grams[4,9]. Assuming line phone sets with cordless handsets weigh 1500g each.

[h] It is assumed that all mobile phones use lithium-ion batteries with LCO cathode materials, and LIBs per product weigh 15g during 1998-2011, and 30g from 2012-2019[9], due to the increasing need of battery lasting time and each cellphone weighs 200g on average.

[i] It is assumed that 75% of television cameras, camcorders and cameras are digital, and the remaining 25% are analog, and all the digital television cameras, camcorders and cameras and 50% analog of which use LCO Li-ion batteries; for the rest 50%, approximately 45% use NiCd batteries, and 5% use NiMH batteries[9]. For LIB batteries, we assume a mass of 100 grams[4], while NiMH and NiCd batteries weigh 30 grams each[4]. Assuming television cameras weigh 1500g, digital cameras weigh 500g, camcorders weigh 700g, so the average weight are regarded as 900g.

[j] There are five types of aircraft engines, it is assumed that the average weight of aircraft engines is 2000kg and each engine contains 55 kilograms cobalt[4].

[k] Assuming the turbo-jets of a thrust not exceeding 25kN weigh 1000kg, and the turbo-jets of a thrust not exceeding 25kN weigh 700kg averagely. And each turbo-jets contains 55 kilograms cobalt[4].

[l] Assuming the average weight of turbo-propellers exceeding 1100kW is 1000kg and that of turbo-propellers not exceeding 1100kW is 700kg. And each turbo-propeller contains 55 kilograms cobalt[4].

[m] Assuming the gas turbines not exceeding 5000kW weigh 500kg and the gas turbines exceeding 5000kW weigh 1500kg averagely. And each gas turbine contains 55 kilograms cobalt[4]. And as for parts of gas turbines and turbojets, their cobalt contents are regarded as 60% of that of the total gas turbine.

[n] There are two types of aircrafts, assuming airplanes with two engines per product, helicopters with one engine per product, and with 55 kilograms cobalt per engine[4,10]. Assuming that helicopters of an unladen weight not exceeding 2000kg weigh 1500kg and that of an unladen weight exceeding 2000kg weigh 3000kg on average, and airplanes of an unladen weight not exceeding 2000kg weigh 1500kg, airplanes of an unladen weight exceeding 2000kg but not exceeding 15,000kg weigh 10000kg and that of an unladen weight exceeding 15,000kg weigh 17000kg on average.

[o] It is assumed that spacecrafts weigh 165000kg per product, a spacecraft has 4 engines, and with 55 kilograms cobalt per engine.

[p] For machine tools for drilling, milling, boring, grinding, sawing, threading, turning and broaching, it is assumed that 10% of machine tools are made of high-speed steel (HSS) and 30% of which add 5% cobalt as material composition in cemented carbides[4].

[q] It is assumed that there are 5% by weight of magnet materials in loudspeakers, 3% of hard disk drive, and 2% of electric motors[11]. Assuming 30% the products use Sm-Co magnets or Al-Ni-Co magnets, which contains 3% cobalt[12].

[r] Assuming that 30% catalysts with nickel or nickel compounds contains cobalt and it is assumed that the cobalt intensity is 1.5% in catalysts for petroleum refining[4]. As for catalysts to produce polyester precursors, we couldn’t find the exact

cobalt intensity per unit product, so it is also assumed the cobalt intensity is the same as that for petroleum refining which is 1.5%.

[s] It is assumed that 5% of pigment contains cobalt, and the average cobalt intensity is 5% by weight[13]. It is assumed that 5% glass and enamels use cobalt to colorize. For increasingly blue but still transparent glass, one can add up to 2% Co and for Darker blue enamels can contain as much as 7% Co[14].

[t] It is assumed that 60% of paint dryers add cobalt as a composition to avoid drying delays, 40% of which contain 0.02% cobalt by weight and 20% of which contain 0.04%[15].

[u] It is assumed that 10% of materials making artificial teeth and bones contain cobalt and the material usually containing 28–68 percent cobalt, with chromium, nickel, and molybdenum, so we assume 50% cobalt is contained in those materials[13].

[v] It is assumed that the average recycling efficiency of all types of cobalt old scrap is 68%[16].

Supplementary Table 2 Detailed calculating processes of historical regional and global cobalt flows and stocks.

Flows	Description	Calculation
$F_{1\text{-}2}$	Cobalt ores from mining process entering the refining process	$F_{1\text{-}2} = F_{2\text{-}3} + F_{2\text{-}0} - F_{25\text{-}2}$
$F_{1\text{-}0}$	Loss in the mining process	$F_{1\text{-}0} = F_{1\text{-}2}/(1 - mining\ loss\ rate) * mining\ loss\ rate$
$F_{2\text{-}0}$	Loss in the refining process	$F_{2\text{-}0} = \sum_n^i F_{3-i}/(1 - refining\ loss\ rate_i) * refining\ loss\ rate_i, i \in n, n = \{4,9,10,11,12,13,14\}$
$F_{2\text{-}3}$	Refinery cobalt entering the market	$F_{2\text{-}3} = F_{3\text{-}4} + F_{3\text{-}9} + F_{3\text{-}10} + F_{3\text{-}11} + F_{3\text{-}12} + F_{3\text{-}13} + F_{3\text{-}14}$
$F_{3\text{-}4}$	Refinery cobalt going to the manufacturing process of all battery end uses	Statistics
$F_{3\text{-}9}$	Refinery cobalt going to the manufacturing process of SA	Statistics
$F_{3\text{-}10}$	Refinery cobalt going to the manufacturing process of CC	Statistics
$F_{3\text{-}11}$	Refinery cobalt going to the manufacturing process of MAG	Statistics
$F_{3\text{-}12}$	Refinery cobalt going to the manufacturing process of CAT	Statistics
$F_{3\text{-}13}$	Refinery cobalt going to the manufacturing process of PI	Statistics
$F_{3\text{-}14}$	Refinery cobalt going to the manufacturing process of OTH	Statistics
$F^t_{0\text{-}i}/F^t_{i\text{-}0}$	Import/export of cobalt final products for each region	Statistics ($i \in \{4,9,10,11,12,13,14\}$)
$F_{5\text{-}5}$	Recycled new scrap of B-PEV reentering the manufacturing process	$F_{5\text{-}5} = F_{5\text{-}15}/(1 - manufacturing\ loss\ rate_5) * new\ scrap\ generation\ rate_5 * new\ scrap\ recycling\ efficiency_5$
$F_{6\text{-}6}$	Recycled new scrap of B-EB reentering the manufacturing process	$F_{6\text{-}6} = F_{6\text{-}16}/(1 - manufacturing\ loss\ rate_6) * new\ scrap\ generation\ rate_6 * new\ scrap\ recycling\ efficiency_6$
$F_{7\text{-}7}$	Recycled new scrap of B-ESS reentering the manufacturing process	$F_{7\text{-}7} = F_{7\text{-}17}/(1 - manufacturing\ loss\ rate_7) * new\ scrap\ generation\ rate_7 * new\ scrap\ recycling\ efficiency_7$
$F_{8\text{-}8}$	Recycled new scrap of B-CE&O reentering the manufacturing process	$F_{8\text{-}8} = F_{8\text{-}18}/(1 - manufacturing\ loss\ rate_8) * new\ scrap\ generation\ rate_8 * new\ scrap\ recycling\ efficiency_8$
$F_{j\text{-}j}$	Recycled new scrap of cobalt-containing products reentering the manufacturing process	$F_{j\text{-}j} = F_{3\text{-}j} * new\ scap\ generation\ rate * new\ scrap\ recycling\ efficiency_j, j \in [9,14]$
$F_{5\text{-}0}$	Loss in the manufacturing process for B-PEV	$F_{5\text{-}0} = F_{5\text{-}15}/(1 - manufacturing\ loss\ rate_5) * manufacturing\ loss\ rate_5$
$F_{6\text{-}0}$	Loss in the manufacturing process for B-EB	$F_{6\text{-}0} = F_{6\text{-}16}/(1 - manufacturing\ loss\ rate_6) * manufacturing\ loss\ rate_6$
$F_{7\text{-}0}$	Loss in the manufacturing process for B-ESS	$F_{7\text{-}0} = F_{7\text{-}17}/(1 - manufacturing\ loss\ rate_7) * manufacturing\ loss\ rate_7$
$F_{8\text{-}0}$	Loss in the manufacturing process for B-CE&O	$F_{8\text{-}0} = F_{8\text{-}18}/(1 - manufacturing\ loss\ rate_8) * manufacturing\ loss\ rate_8$
$F_{j\text{-}0}$	Loss in the manufacturing	$F_{j\text{-}0} = F_{3\text{-}j} * manufacturing\ loss\ rate_j, j \in [9,14]$

	process for cobalt final products	
$F_{5\text{-}15}$	Cobalt containing final products of B-PEV entering use phase	$F_{5\text{-}15} = cobalt\ intensity * battery\ capacity * PEV\ demand$
$F_{6\text{-}16}$	Cobalt containing final products of B-EB entering use phase	$F_{6\text{-}16} = cobalt\ intensity * battery\ capacity * EB\ demand$
$F_{7\text{-}17}$	Cobalt containing final products of B-ESS entering use phase	$F_{7\text{-}17} = cobalt\ intensity * ESS\ demand$
$F_{8\text{-}18}$	Cobalt containing final products of B-CE&O entering use phase	$F_{8\text{-}18} = F_{3\text{-}4} - (F_{5\text{-}15} + F_{5\text{-}0}) - (F_{6\text{-}16} + F_{6\text{-}0}) - (F_{7\text{-}17} + F_{7\text{-}0}) - F_{8\text{-}0}$
$F_{9\text{-}19}$	Cobalt containing final products of SA entering use phase	$F_{9\text{-}19} = F_{3\text{-}9} + F_{9\text{-}9} - F_{9\text{-}9} - F_{9\text{-}0}$
$F_{10\text{-}20}$	Cobalt containing final products of CC entering use phase	$F_{10\text{-}20} = F_{3\text{-}10} + F_{10\text{-}10} - F_{10\text{-}10} - F_{10\text{-}0}$
$F_{11\text{-}21}$	Cobalt containing final products of MAG entering use phase	$F_{11\text{-}21} = F_{3\text{-}11} + F_{11\text{-}11} - F_{11\text{-}11} - F_{11\text{-}0}$
$F_{12\text{-}22}$	Cobalt containing final products of CAT entering use phase	$F_{12\text{-}22} = F_{3\text{-}12} + F_{12\text{-}12} - F_{12\text{-}12} - F_{12\text{-}0}$
$F_{13\text{-}23}$	Cobalt containing final products of PI entering use phase	$F_{13\text{-}23} = F_{3\text{-}13} + F_{13\text{-}13} - F_{13\text{-}13} - F_{13\text{-}0}$
$F_{14\text{-}24}$	Cobalt containing final products of OTH entering the use phase	$F_{14\text{-}24} = F_{3\text{-}14} + F_{14\text{-}14} - F_{14\text{-}14} - F_{14\text{-}0}$
$F_{15\text{-}25}$	Obsolete cobalt-containing products of B-PEV going to waste management process	$F_{15\text{-}25}(t) = \sum_R^r \int_{t_0}^t F_{5-15}^r(\tau) * \left(\frac{1}{\sigma*\sqrt{2*\pi}} * e^{-\frac{(\tau-\mu)^2}{2\sigma^2}}\right) r \in R, R = \{China, U.S., Japan, EU, ROW\}$[17,18]
$F_{16\text{-}25}$	Obsolete cobalt-containing products of B-EB going to waste management process	$F_{16\text{-}25}(t) = \sum_R^r \int_{t_0}^t F_{6-16}^r(\tau) * \left(\frac{1}{\sigma*\sqrt{2*\pi}} * e^{-\frac{(\tau-\mu)^2}{2\sigma^2}}\right) r \in R, R = \{China, U.S., Japan, EU, ROW\}$
$F_{17\text{-}25}$	Obsolete cobalt-containing products of B-ESS going to waste management process	$F_{17\text{-}25}(t) = \sum_R^r \int_{t_0}^t F_{7-17}^r(\tau) * \left(\frac{1}{\sigma*\sqrt{2*\pi}} * e^{-\frac{(\tau-\mu)^2}{2\sigma^2}}\right) r \in R, R = \{China, U.S., Japan, EU, ROW\}$
$F_{18\text{-}25}$	Obsolete cobalt-containing products of B-CE&O going to waste management process	$F_{18\text{-}26}(t) = \sum_R^r \int_{t_0}^t F_{8-18}^r(\tau) * \left(\frac{1}{\sigma*\sqrt{2*\pi}} * e^{-\frac{(\tau-\mu)^2}{2\sigma^2}}\right) r \in R, R = \{China, U.S., Japan, EU, ROW\}$
$F_{19\text{-}25}$	Obsolete cobalt-containing products of SA going to waste management process	$F_{19\text{-}25}(t) = \sum_R^r \int_{t_0}^t F_{9-19}^r(\tau) * \left(\frac{1}{\sigma*\sqrt{2*\pi}} * e^{-\frac{(\tau-\mu)^2}{2\sigma^2}}\right) r \in R, R = \{China, U.S., Japan, EU, ROW\}$
$F_{20\text{-}25}$	Obsolete cobalt-containing products of CC going to waste management process	$F_{20\text{-}25}(t) = \sum_R^r \int_{t_0}^t F_{10-20}^r(\tau) * \left(\frac{1}{\sigma*\sqrt{2*\pi}} * e^{-\frac{(\tau-\mu)^2}{2\sigma^2}}\right) r \in R, R = \{China, U.S., Japan, EU, ROW\}$
$F_{21\text{-}25}$	Obsolete cobalt-containing products of MAG going to waste management process	$F_{21\text{-}25}(t) = \sum_R^r \int_{t_0}^t F_{11-21}^r(\tau) * \left(\frac{1}{\sigma*\sqrt{2*\pi}} * e^{-\frac{(\tau-\mu)^2}{2\sigma^2}}\right) r \in R, R = \{China, U.S., Japan, EU, ROW\}$
$F_{22\text{-}25}$	Obsolete cobalt-containing products of CAT going to waste	$F_{22\text{-}25}(t) = \sum_R^r \int_{t_0}^t F_{12-22}^r(\tau) * \left(\frac{1}{\sigma*\sqrt{2*\pi}} * e^{-\frac{(\tau-\mu)^2}{2\sigma^2}}\right) r \in R, R = \{China, U.S., Japan, EU, ROW\}$

	management process	
$F_{23\text{-}25}$	Obsolete cobalt-containing products of PI going to waste management process	$F_{23\text{-}25}(t) = \sum_R^r \int_{t_0}^t F_{13-23}^j(\tau) * \left(\frac{1}{\sigma*\sqrt{2*\pi}} * e^{-\frac{(\tau-\mu)^2}{2\sigma^2}}\right) r \in R, R = \{China, U.S., Japan, EU, ROW\}$
$F_{24\text{-}25}$	Obsolete cobalt-containing products of OTH going to waste management process	$F_{24\text{-}25}(t) = \sum_R^j \int_{t_0}^t F_{14-24}^r(\tau) * \left(\frac{1}{\sigma*\sqrt{2*\pi}} * e^{-\frac{(\tau-\mu)^2}{2\sigma^2}}\right) r \in R, R = \{China, U.S., Japan, EU, ROW\}$
$F^t_{0\text{-}25}/F^t_{25\text{-}0}$	Import/export of cobalt waste and scrap for each region	Statistics
$F_{25\text{-}0}$	Loss in the waste management	$F_{25\text{-}0}= \sum_R^r \sum_m^k F_{k-25}^r * (1 - EoL\ recycling\ rate_k), k \in m, m = [15,24]; r \in R, R = \{China, U.S., Japan, EU, ROW\}$
$F_{25\text{-}2}$	Cobalt scrap recycled from EoL products reenter into the refining process	$F_{25\text{-}2}=\sum_R^r \sum_m^k F_{k-25}^r * EoL\ recycling\ rate_k, k \in m, m = [15,24]; r \in R, R = \{China, U.S., Japan, EU, ROW\}$

Note: The flows described below correspond to Supplementary Figure 2. The coefficients mentioned below are listed in Supplementary Table 5.

Supplementary Table 1 The global and regional cobalt apparent consumption.

Regions	Categories	Periods	Data sources
China	Total amount	1998-2013	JOGMEC[19]
		2006-2019	CNIA-CO[20]
	Quantity by end-uses	2006-2019	CNIA-CO[20]
US	Total amount	1998-2019	USGS[21]
	Quantity by end-uses	1998-2019	USGS[21]
Japan	Total amount	1998-2019	JOGMEC[19]
	Quantity by end-uses	2010-2015	JOGMEC[19]
EU	Total amount	1998-2019	JOGMEC[19], BGS[22], UN Comtrade[23]
	Quantity by end-uses	2012	Slavko et al. (2015)[24]
Global	Total amount	1998-2019	JOGMEC[19], USGS[21], Chen et al. (2019)[25]
	Quantity by end-uses	2010-2015	Antaike[26]

Note: Historical regional cobalt apparent consumption by end-uses during 1998-2019 are shown in Supplementary Figure 20. Values for the other years without reported data are assumed based on the existing data.

Supplementary Table 4 Key parameters for bottom-up and historical quantification of cobalt demand for emerging end-uses over the five regions.

Parameters	End uses	Regions	Historical values/Assumptions
Cobalt intensity[5–7] (kg/kWh)	B-PEV<br>B-EB<br>B-ESS	All	NMC-111: 0.394<br>NMC-433: 0.312<br>NMC-532: 0.23<br>NMC-622: 0.214<br>NMC-811: 0.094 NCA: 0.143<br>LMO: 0<br>LFP: 0
Market shares of battery cathode chemistry in 2019	B-PEV	China[27]	NMC-111: 9%; NMC-622: 40%; NMC-811: 11%; NCA: 10%; LFP: 30%
		U.S.[28]	NMC-111: 5%; NMC-433: 10%; NMC-622: 5%; NMC-811: 4%; NCA: 75%; LFP: 1%
		Japan[29]	NMC-111: 21%; NMC-433: 5%; NMC-532: 28%; NMC-622: 34%; NMC-811: 8%; NCA: 3%; LFP: 1%
		EU[30]	NMC-111: 7%; NMC-622: 34%; NMC-811: 30%; NCA: 28%; LFP: 1%
		ROW[30]	NMC-111: 11%; NMC-532: 28%; NMC-622: 34%; NMC-811: 6%; NCA: 20%; LFP: 1%
	B-EB	All[2]	NMC-111: 12%; NCA: 2%; LFP: 86%
	B-ESS	All[31]	NMC-111: 64%; NCA: 18%; LFP: 18%
Battery capacity of BEV/PHEV (kWh)	B-PEV[8]	China	32/16
		U.S.	58/13
		Japan	30/12
		EU	33/10
		ROW	20/10
	B-EB	All[1,2]	81/21
ESS stock (GWh) by 2019	B-ESS[32,33]	China	2.033
		U.S.	2.440
		Japan	1.626
		EU	2.033
		ROW	0.407

Supplementary Table 5 Assumptions for average product lifetime, end-of-life recycling rate (EoL-RR), and the loss rate of each process for cobalt end-use sectors.

	Average product lifetime (Year)	End-of-life recycling rate (%)[34]	New scrap generation rate (%)[35]	New scrap recycling efficiency (%)[35]	Manufacturing loss rate (%)[35]	Refining loss rate (%)	Mining loss rate (%)
Battery-PEV	8[4]	80	1	0	1	10[36]	35[37]
Battery-EB	7[2]	80	1	0	1		
Battery-ESS	10[38]	80	1	0	1		
Battery-CE&O	3[25]	10	1	0	1		
Superalloys	5[25]	80	79	80	16		
Cemented carbides	1[25]	45[4]	0	0	1		
Magnets	5[25]	5	10	80	2		
Catalysts	5.5[4]	53	0	0	5		
Pigments	5[39]	0	0	0	1		
Others	1[25]	0	0	0	1		

Note: The initial cobalt in-use stock of each end use by 1997 is regarded as zero due to data deficiency. A normal distribution is used for the lifetime in this paper.

Supplementary Table 6 Assumptions of key parameters for battery cathode technology scenarios per region for emerging end uses.

Parameters	Scenarios (by 2050)			
	BT1	BT2	BT3	BT4
Cobalt intensity (kg/kWh)	NMC-111:0.394; NMC-433:0.312; NMC-532:0.23; NMC-622:0.214; NMC-811:0.094; NCA:0.143; LMO/LFP(Ⅰ)/ LFP(Ⅱ): 0; NMC-9.5.5/NCA(II):0.05[40]; Li-S/Li-air/SSB: 0[5–7,40]			
Battery cathode material market shares	State-of-the-art technology occupies 100% of the market. And the cathode shares of the state-of-the-art technologies are assumed shifting from NMC-111 towards NCA and NMC-811[41,42]	Low-cobalt battery cathode technologies (NMC-9.5.5[30,43,44] and advanced NCA[45]) penetrate the market from 2020 (100% by 2050)[45,46]	New LFP battery cathode technology gradually dominants the market (100% by 2050)[47]	Next-generation battery technologies (Li-S/Li-air[48]/SSB[49]) penetrate the market of BT1 from 2030 (100% by 2050)[50]

Supplementary Table 7 Battery lifetime scenarios per region for emerging end uses.

Parameters	End uses	Regions	Scenarios	
			Base	Extended[54,55]
Battery lifetime	B-PEV	All	8 years[4]	16 years
	B-EB	All	7 years[2]	14 years
	B-ESS	All	10 years[38]	20 years

Supplementary Table 8 Assumptions of recycling scenarios for all end uses over five regions.

Parameters	End uses	Regions	Scenarios (by 2050)	
			Base[4,34]	High
Recycling rate	B-PEV	All	88%	95% by 2050 following an S-shape curve
	B-EB		88%	
	B-ESS		88%	
	B-CE&O		11%	
	SA		88%	
	CC		49%	
	MAG		5%	
	CAT		58%	
	PI		0%	0%
	OTH		0%	0%

Supplementary Table 9 The assumptions for other parameters by 2050 and variation range in sensitivity analysis per region for emerging end uses.

Parameters	End uses	Regions	Value (2050)	Variation range	
				Low	High
The share of BEV/PHEV in EV sales[56]	B-PEV	All	90%/10%	90%/10%	90%/10%
	B-EB	All	95%/5%	95%/5%	95%/5%
Battery capacity of BEV/PHEV (kWh)[56]	B-PEV	China	120/30	82/20	158/40
		U.S.	150/50	102/34	198/66
		Japan	120/30	82/20	158/40
		EU	120/30	82/20	158/40
		ROW	70/15	47/10	92/19
	B-EB	All	320/80	218/54	422/106
EV market share[56]	B-PEV	China	50%	30%	80%
		U.S.	50%	30%	70%
		Japan	50%	30%	70%
		EU	60%	40%	80%
		ROW	25%	15%	35%
	B-EB	China	80%	70%	90%
		U.S.	25%	15%	35%
		Japan	30%	15%	45%
		EU	70%	55%	85%
		ROW	25%	10%	40%
Vehicle ownership (vehicles per 1000 people)	B-PEV[a]	China	400	300	500
		U.S.	850	720	900
		Japan	570	468	620
		EU	600	495	650
		ROW	200	100	300
	B-EB	China	3	3	4
		U.S.	9	7	10
		Japan	9	7	10
		EU	6	5	7
		ROW	3	2	3
ESS stock[b] (GWh)	B-ESS[57]	China	250	200	300
		U.S.	300	240	360
		Japan	200	160	240
		EU	250	200	300
		ROW	50	40	60

[a] The vehicle ownership of PEV for the U.S., the EU and Japan are relatively higher, so the low scenarios of which decrease 10% more considering the prevalence of shared mobility.

[b] The shares of ESS stocks among five regions are set according to per capita renewable electricity generation[33], considering the close relationship between ESS and renewable energy.

Supplementary Table 2 The assumptions for other parameters by 2050 and variation range in sensitivity analysis per region for traditional end uses.

Parameters	End uses	Regions	Value (2050)	Variation range	
				Low (-20%)	High (+20%)
Cobalt stock per capita (kg per 1000 people)	B-CE&O	China	100.5	80.4	120.7
		U.S.	100.6	80.5	120.7
		Japan	458.0	366.4	549.6
		EU	151.1	120.9	181.3
		ROW	27.1	21.7	32.5
	SA	China	76.0	60.8	91.2
		U.S.	136.4	109.1	163.6
		Japan	140.8	112.7	169.0
		EU	156.3	125.1	187.6
		ROW	12.3	9.8	14.8
	CC	China	11.5	9.2	13.8
		U.S.	8.8	7.1	10.6
		Japan	7.4	5.9	8.9
		EU	17.2	13.8	20.7
		ROW	2.1	1.7	2.5
	MAG	China	22.7	18.2	27.2
		U.S.	8.8	7.0	10.6
		Japan	1.2	1.0	1.5
		EU	8.6	6.9	10.3
		ROW	4.1	3.3	4.9
	CAT	China	11.7	9.4	14.0
		U.S.	16.0	12.8	19.2
		Japan	3.8	3.0	4.5
		EU	18.6	14.9	22.3
		ROW	3.2	2.5	3.8
	PI	China	17.9	14.3	21.5
		U.S.	4.6	3.7	5.5
		Japan	3.5	2.8	4.2
		EU	10.8	8.6	12.9
		ROW	6.4	5.2	7.7
	OTH	China	1.1	0.9	1.3
		U.S.	13.1	10.4	15.7
		Japan	2.0	1.6	2.4
		EU	2.1	1.7	2.6
		ROW	0.6	0.5	0.8

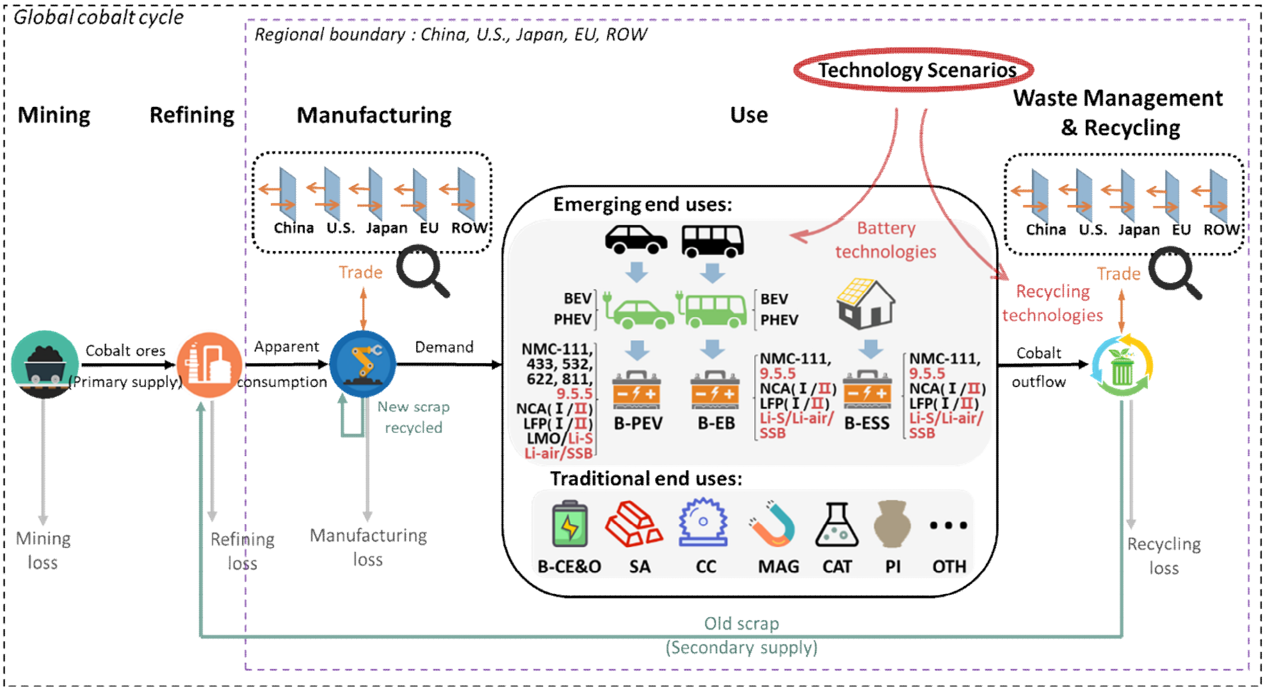

Supplementary Figure 1 System definition of the global anthropogenic cobalt cycle and modeling framework for cobalt demand and secondary supply potentials. BEV: battery electric vehicles. PHEV: plug-in hybrid electric vehicles. NMC: lithium nickel manganese cobalt oxide. NCA(I): lithium nickel cobalt aluminum oxide. NCA(II): advanced NCA with lower cobalt content and higher energy density. LFP(I): lithium iron phosphate. LFP(II): a new form of LFP (e.g., blade LFP battery developed by the company BYD). LMO: lithium manganese oxide. Li-air: lithium-air. Li-S: lithium-sulfur. SSB: solid-state battery. B-PEV: battery for electric passenger vehicles. B-EB: battery for electric buses. B-ESS: battery for energy storage systems. B-CE&O: battery for consumer electronics and other battery products. SA: superalloys. CC: cemented carbides. MAG: magnets. CAT: catalysts. PI: pigments. OTH: other end uses. Battery cathodes in black and red colors indicate state-of-the-art battery technologies and future battery technologies, respectively.

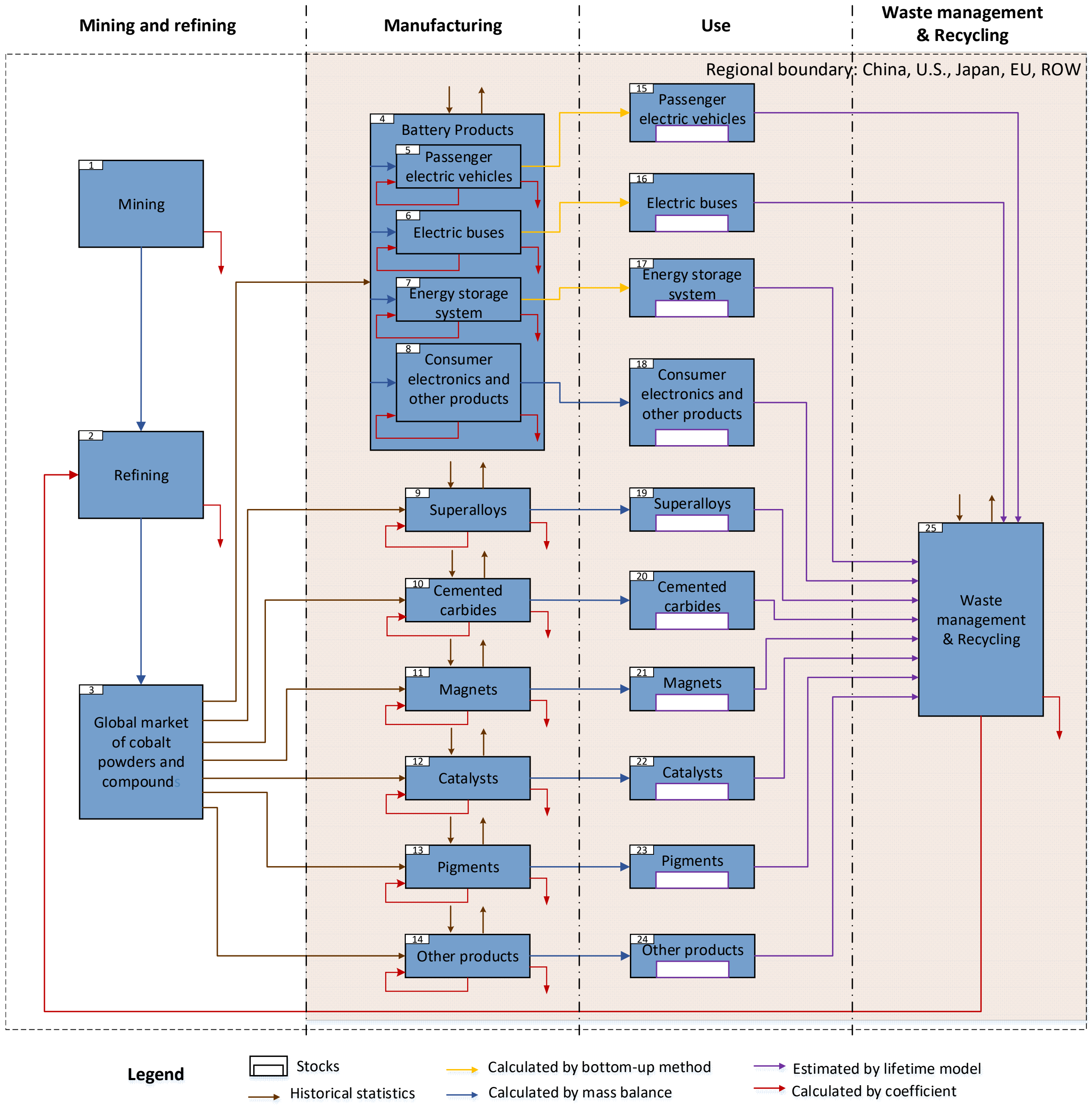

Supplementary Figure 2 Historical quantification of the global anthropogenic cobalt cycle with regional resolution. Transformation processes and market processes are displayed in boxes with a specific number. Flows are distinguished by different colors. All quantifications of cobalt stocks and flows are cobalt equivalent.

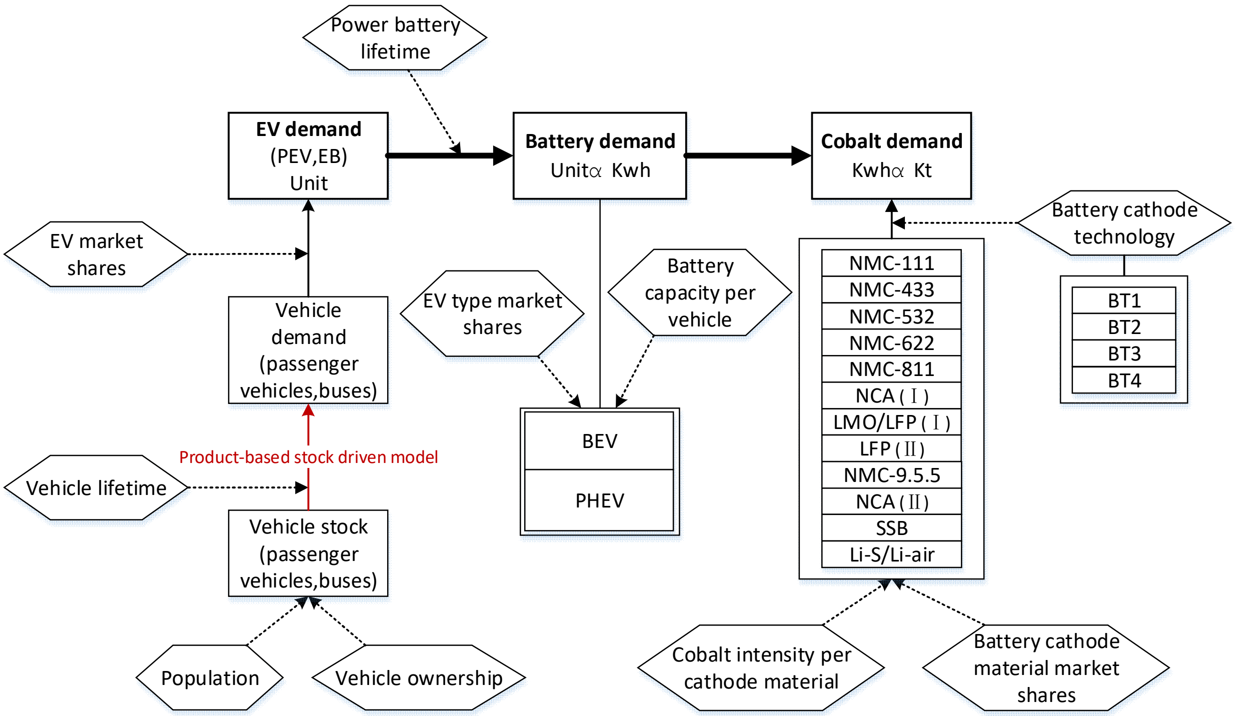

Supplementary Figure 3 Framework of stock driven modelling for B-PEV and B-EB.

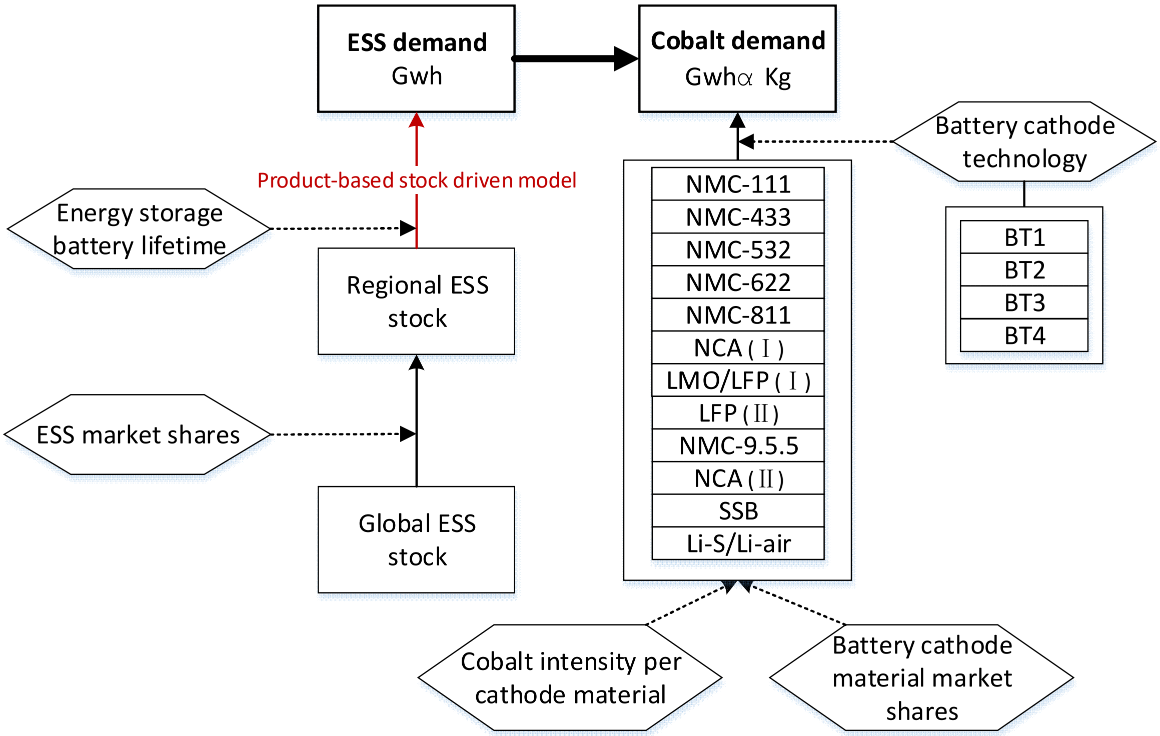

Supplementary Figure 4 Framework of stock-driven modelling for B-ESS.

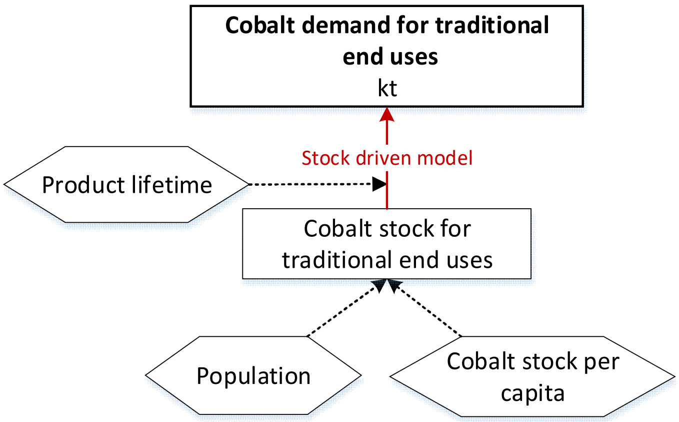

Supplementary Figure 5 Framework of stock-driven modelling for traditional end uses.

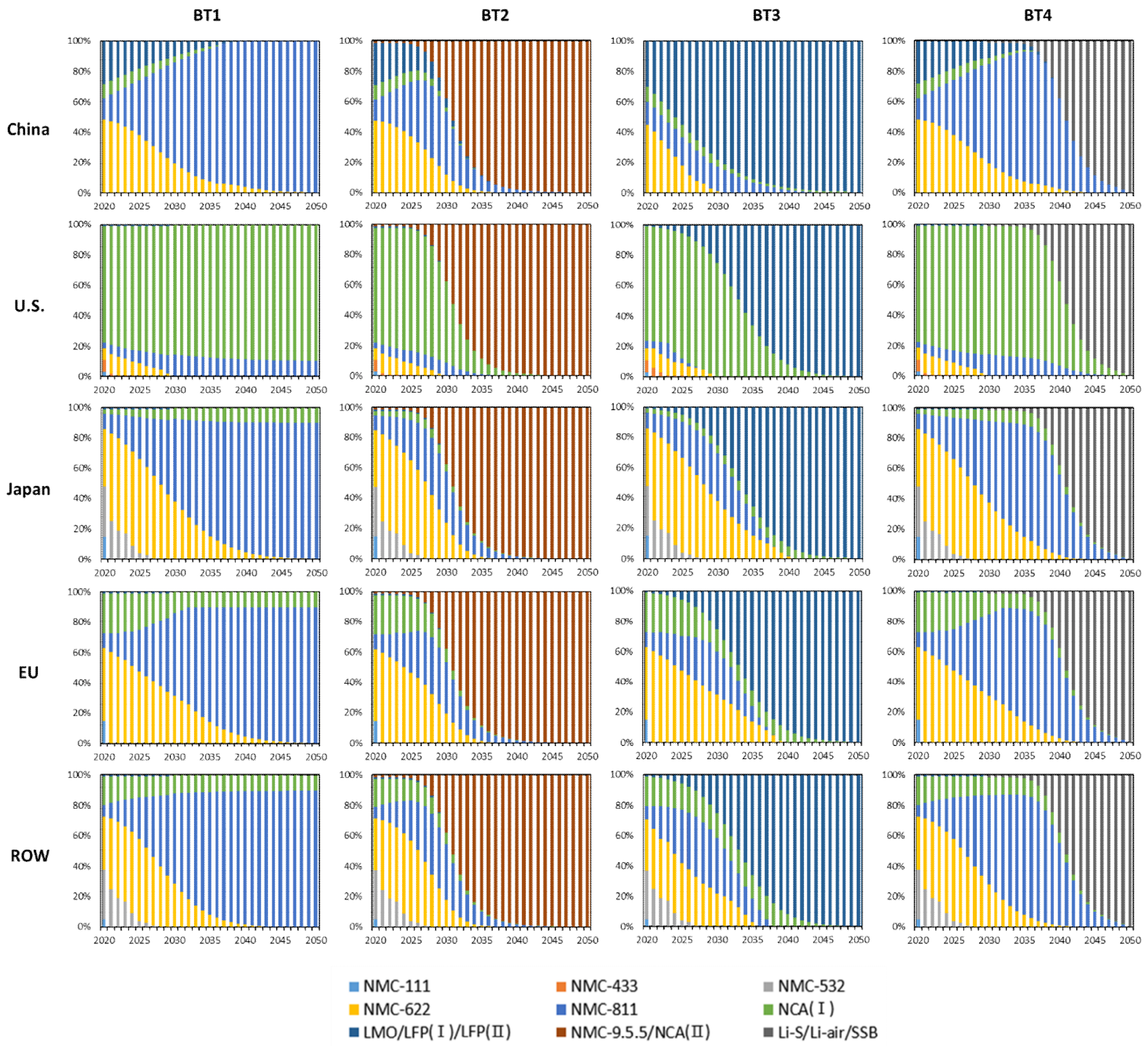

Supplementary Figure 6 Scenarios of battery cathode material market shares for B-PEV per region from 2020 to 2050. B-PEV: battery for passenger electric vehicles; NMC: lithium nickel manganese cobalt oxide. NCA(I): lithium nickel cobalt aluminum oxide. NCA(II): advanced NCA with less cobalt. LFP(I): lithium iron phosphate. LFP(II): a new form of LFP with higher energy density (e.g., blade LFP battery developed by the company BYD). LMO: lithium manganese oxide. Li-air: lithium-air battery. Li-S: lithium-sulfur battery. SSB: solid-state battery.

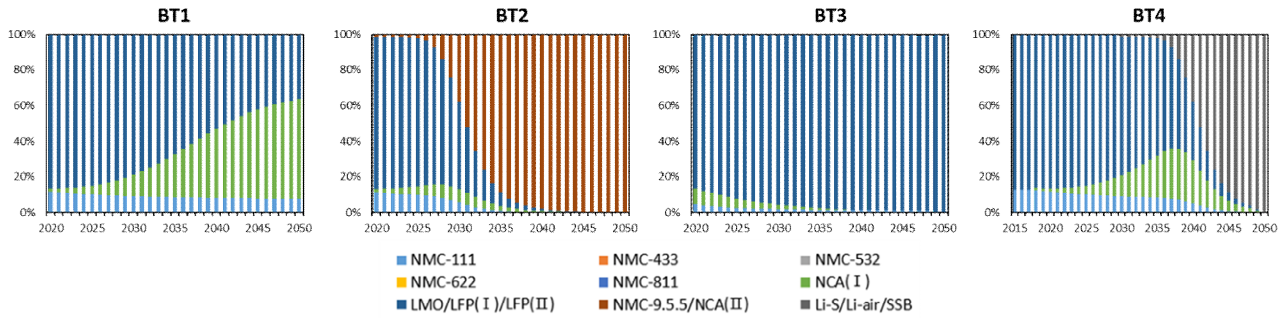

Supplementary Figure 7 Scenarios of battery cathode material market shares for B-EB per region from 2020 to 2050.

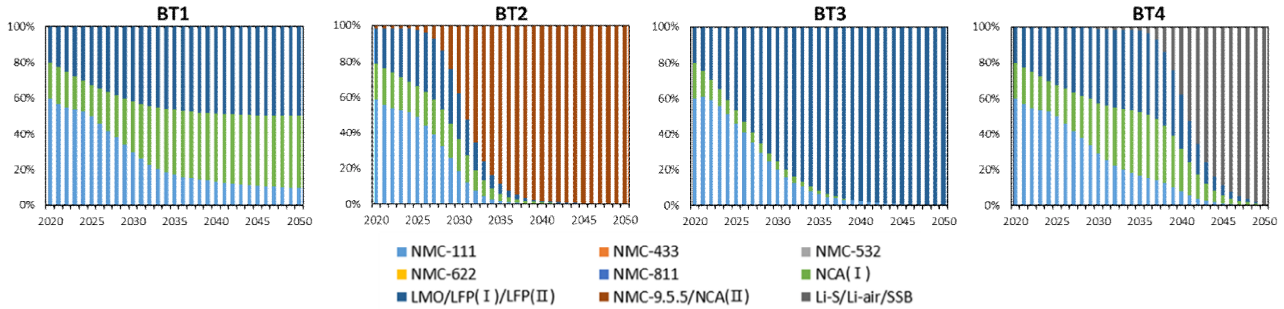

Supplementary Figure 8 Scenarios of battery cathode material market shares for B-ESS per region from 2020 to 2050.

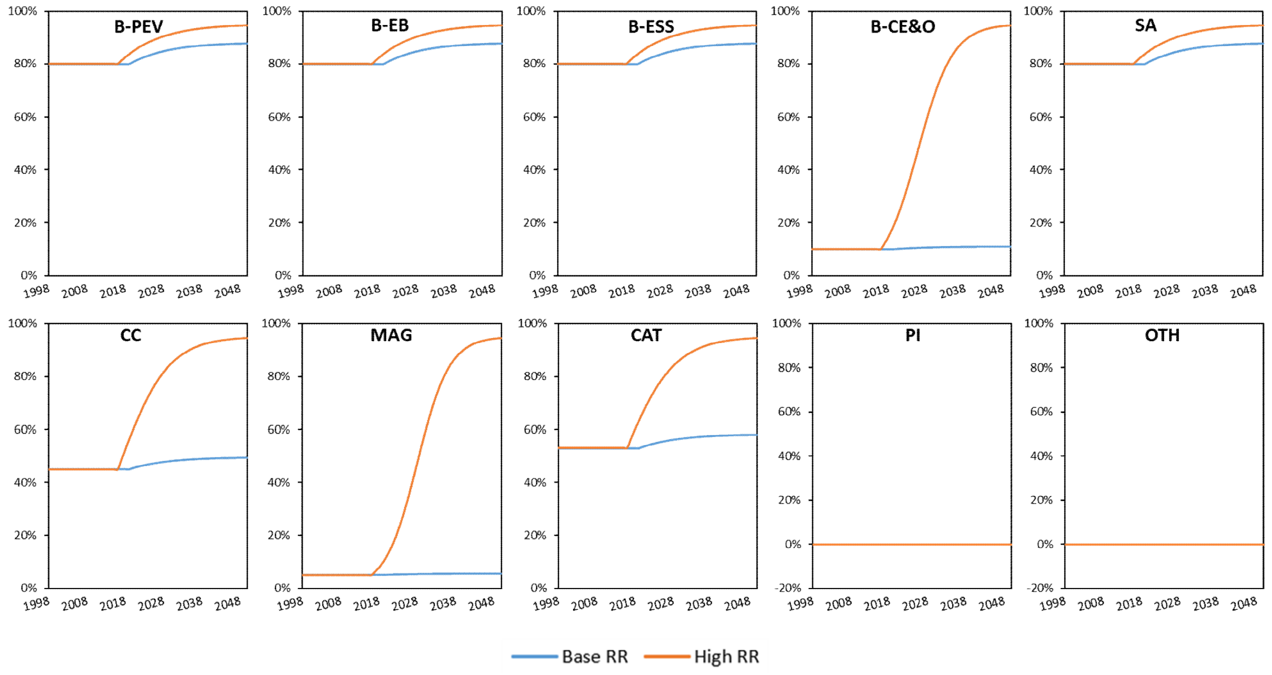

Supplementary Figure 9 Scenarios of cobalt EoL-RR by end uses.

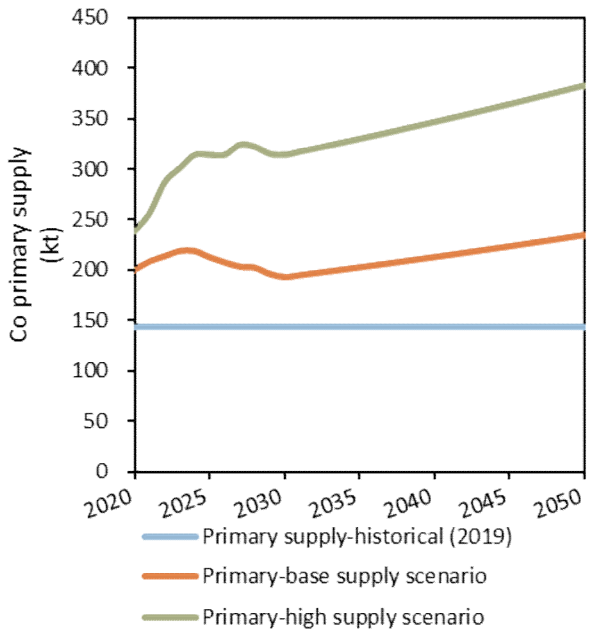

Supplementary Figure 10 Scenarios of global cobalt primary supply from 2020 to 2050 in comparison with historical level.

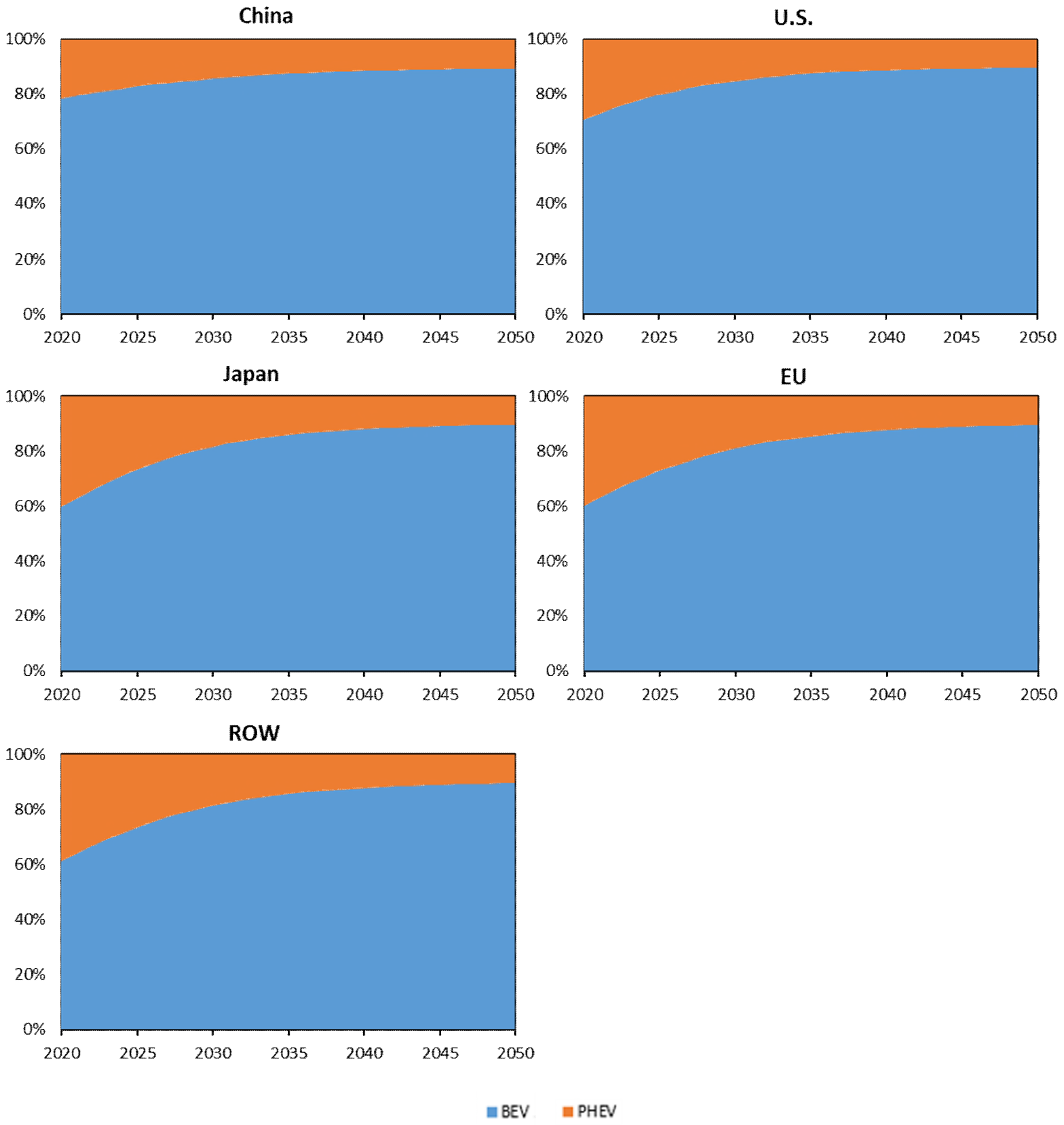

Supplementary Figure 11 The share of BEV/PHEV in B-PEV sales per region from 2020 to 2050. B-PEV: battery for passenger electric vehicles; BEV: battery electric vehicles; PHEV: plug-in hybrid electric vehicles.

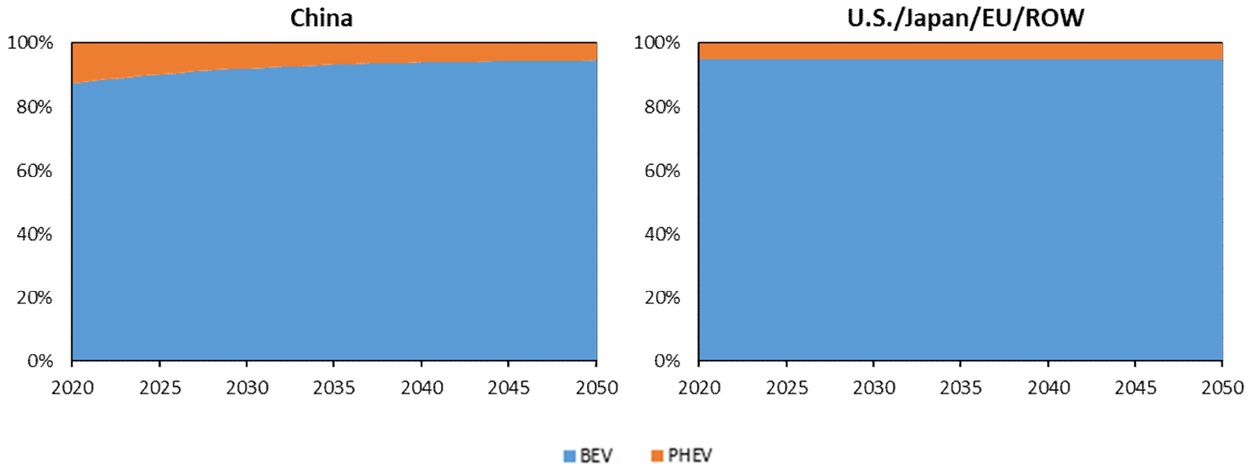

Supplementary Figure 12 The share of BEV/PHEV in B-EB sales per region from 2020 to 2050.

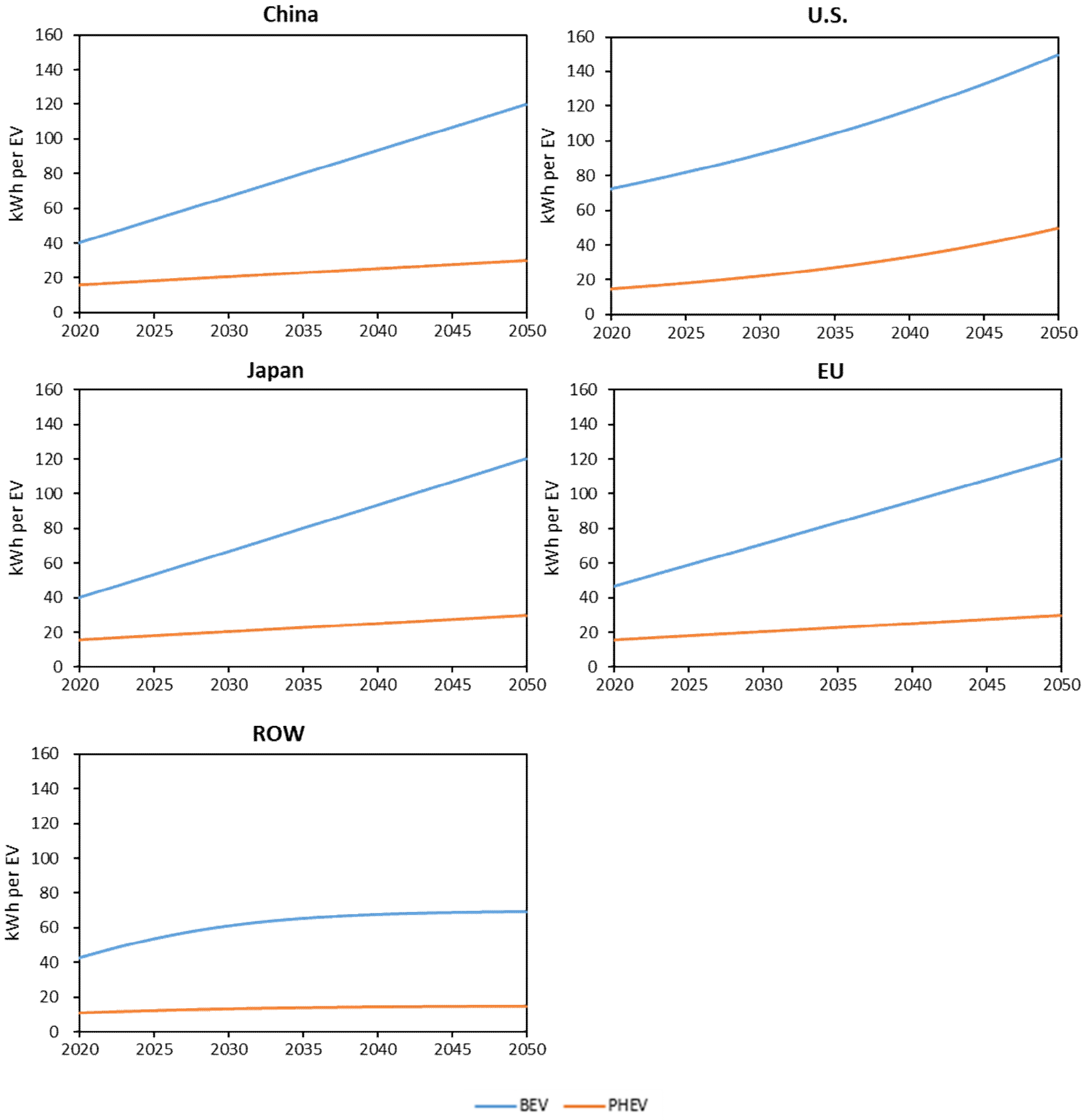

Supplementary Figure 13 Battery capacity of BEV and PHEV for B-PEV per region from 2020 to 2050.

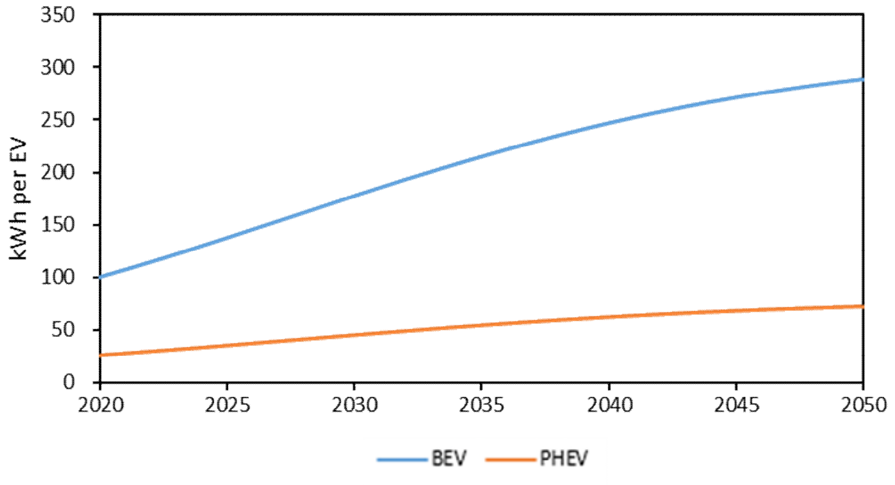

Supplementary Figure 14 Battery capacity of BEV and PHEV for B-EB from 2020 to 2050. All battery capacity assumptions are the same for all regions.

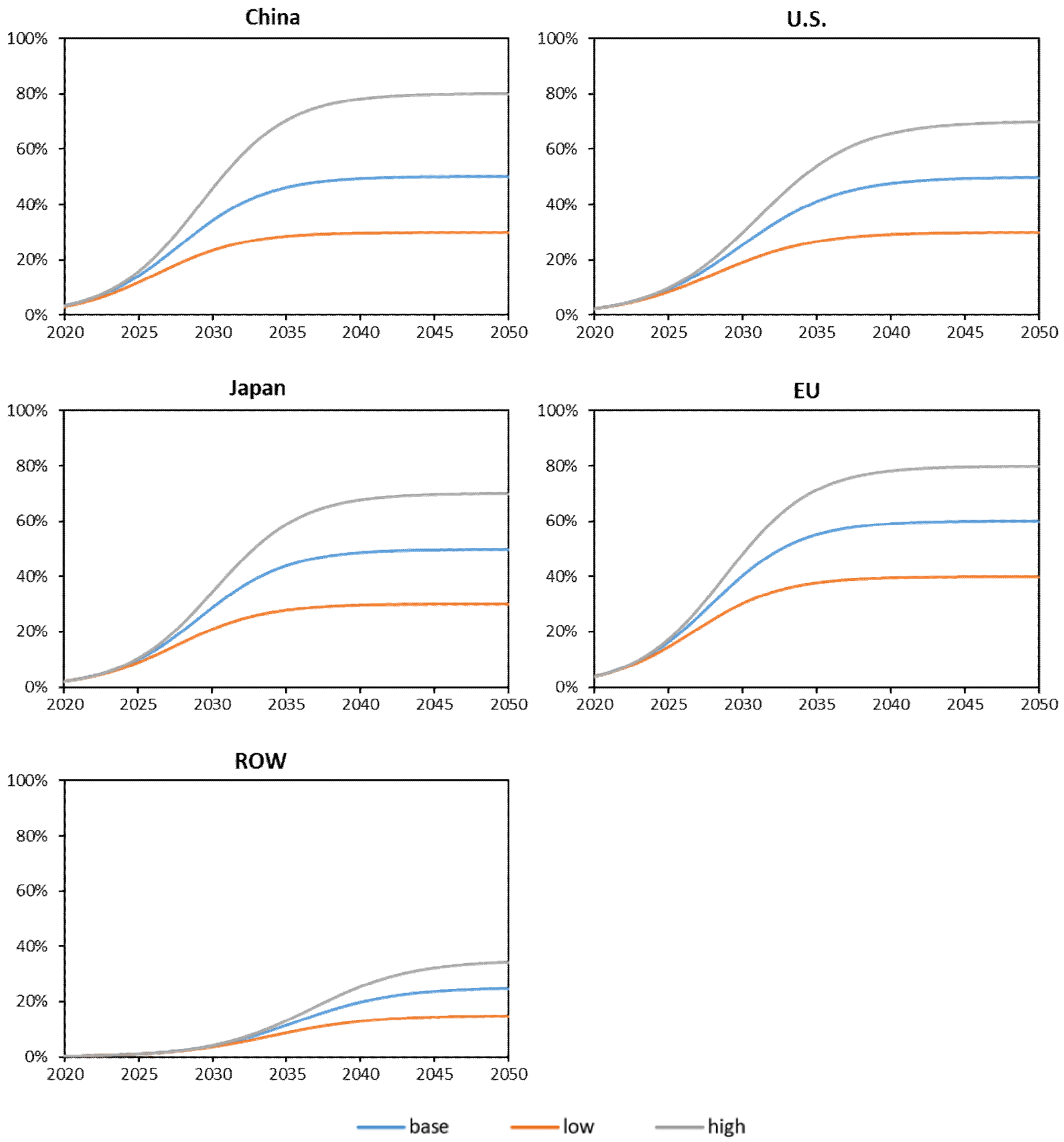

Supplementary Figure 15 Market shares and variation range of passenger electric vehicles per region from 2020 to 2050.

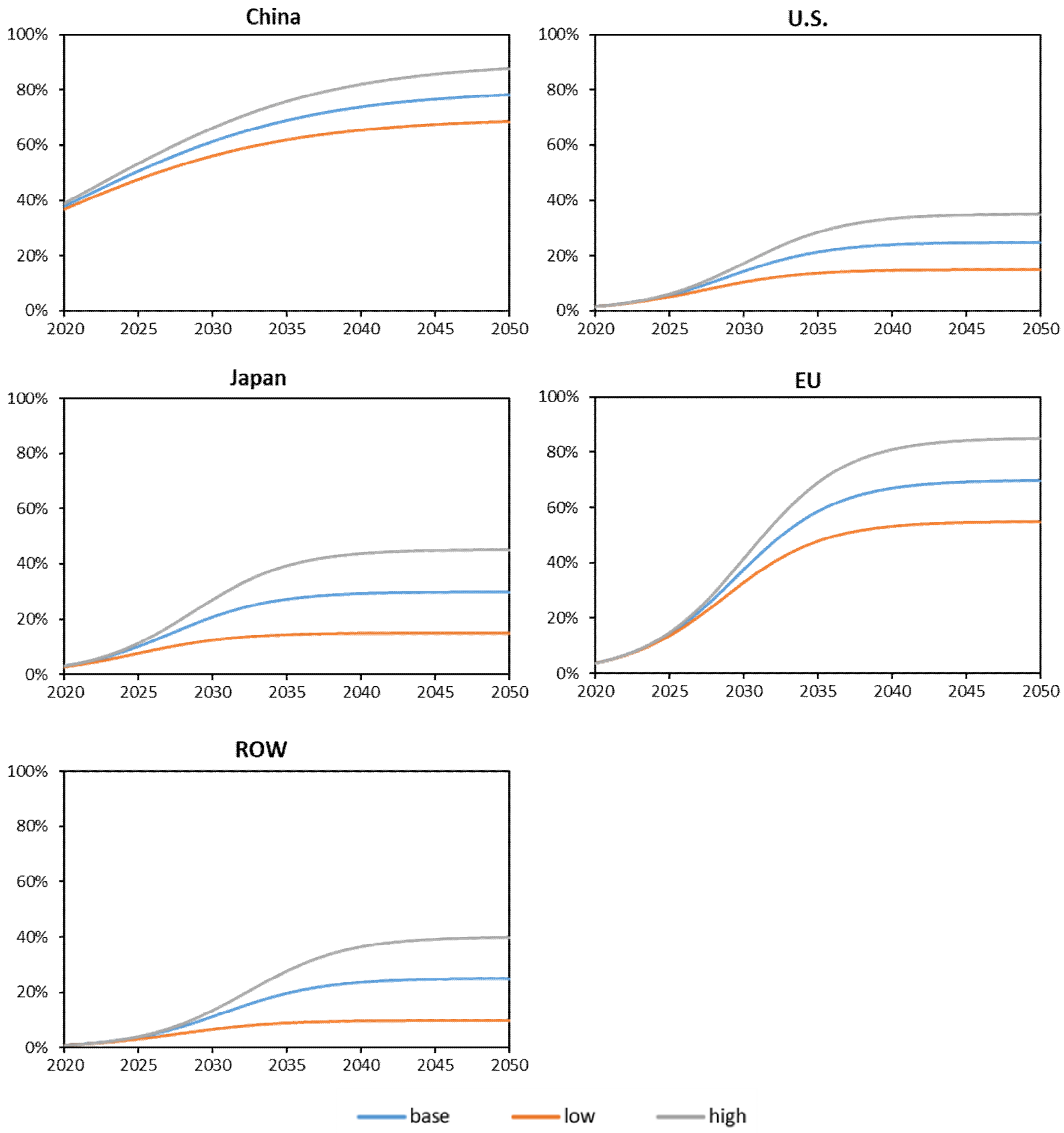

Supplementary Figure 16 Market shares and variation range of electric buses per region from 2020 to 2050.

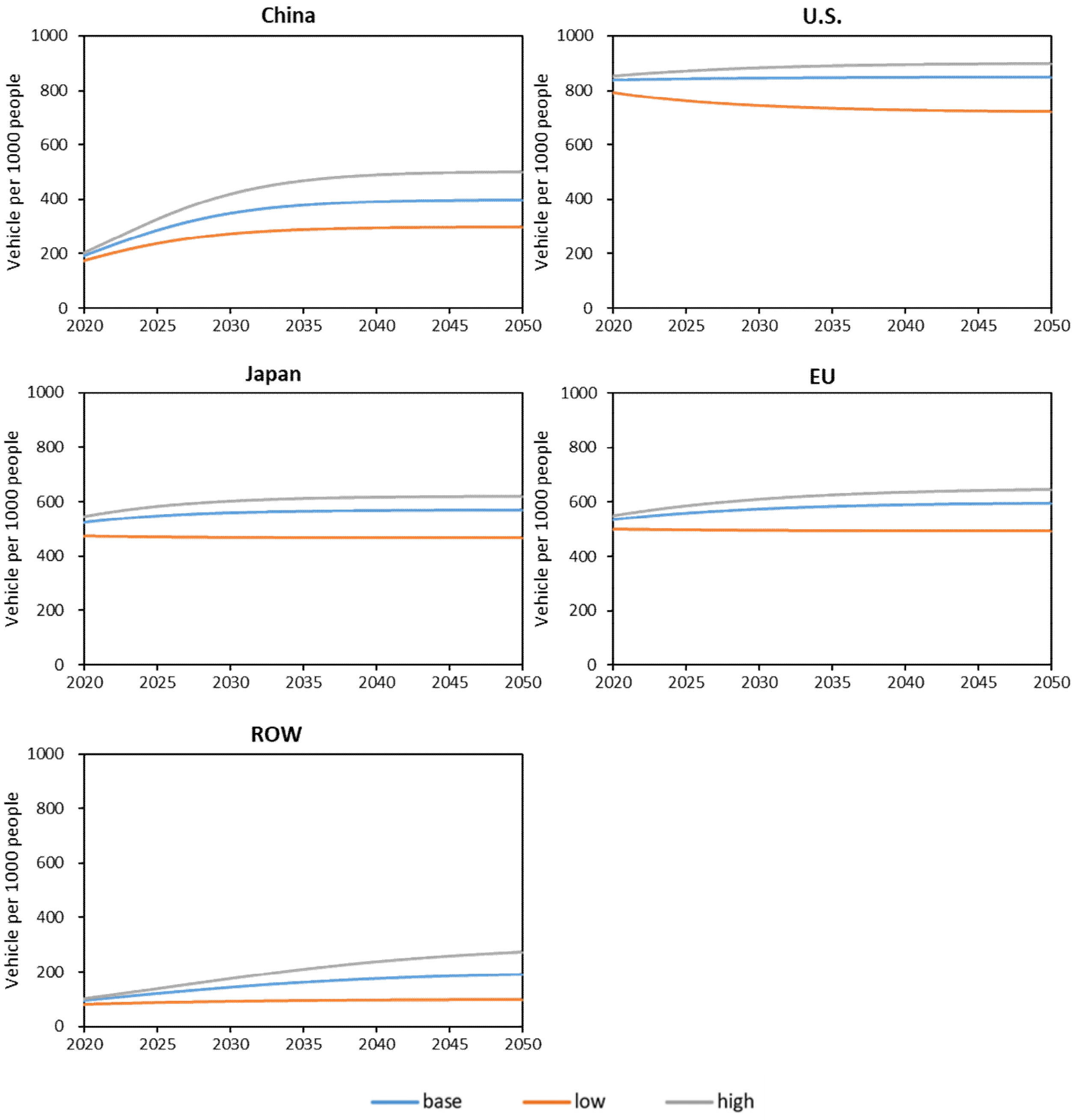

Supplementary Figure 3 Passenger vehicle ownership and variation range per region from 2020 to 2050. The adoption of shared mobility is considered in the low scenarios (10% more reduction) of the U.S., the EU, and Japan, considering their relatively higher vehicle ownership.

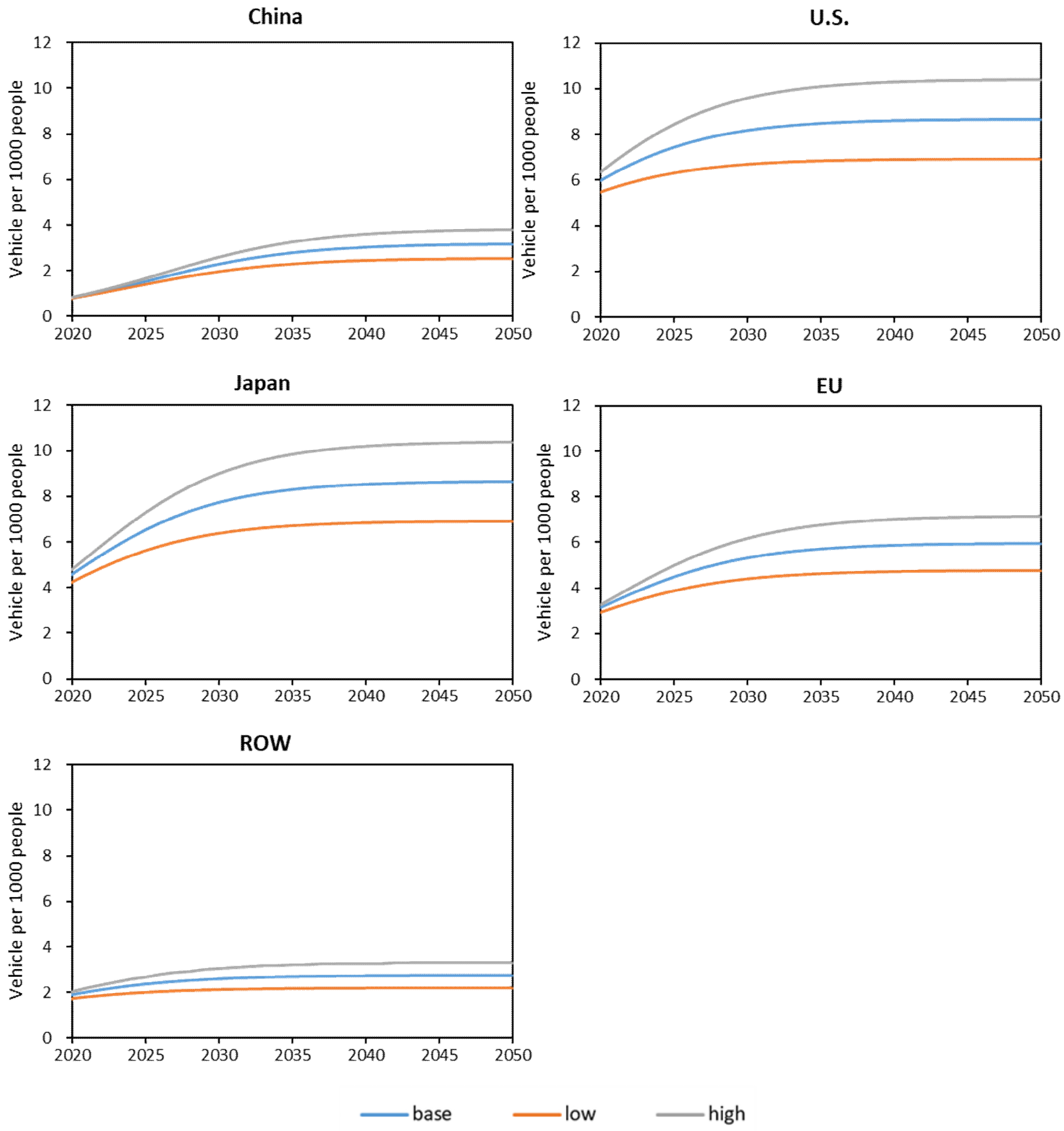

Supplementary Figure 4 Bus ownership and variation range per region from 2020 to 2050.

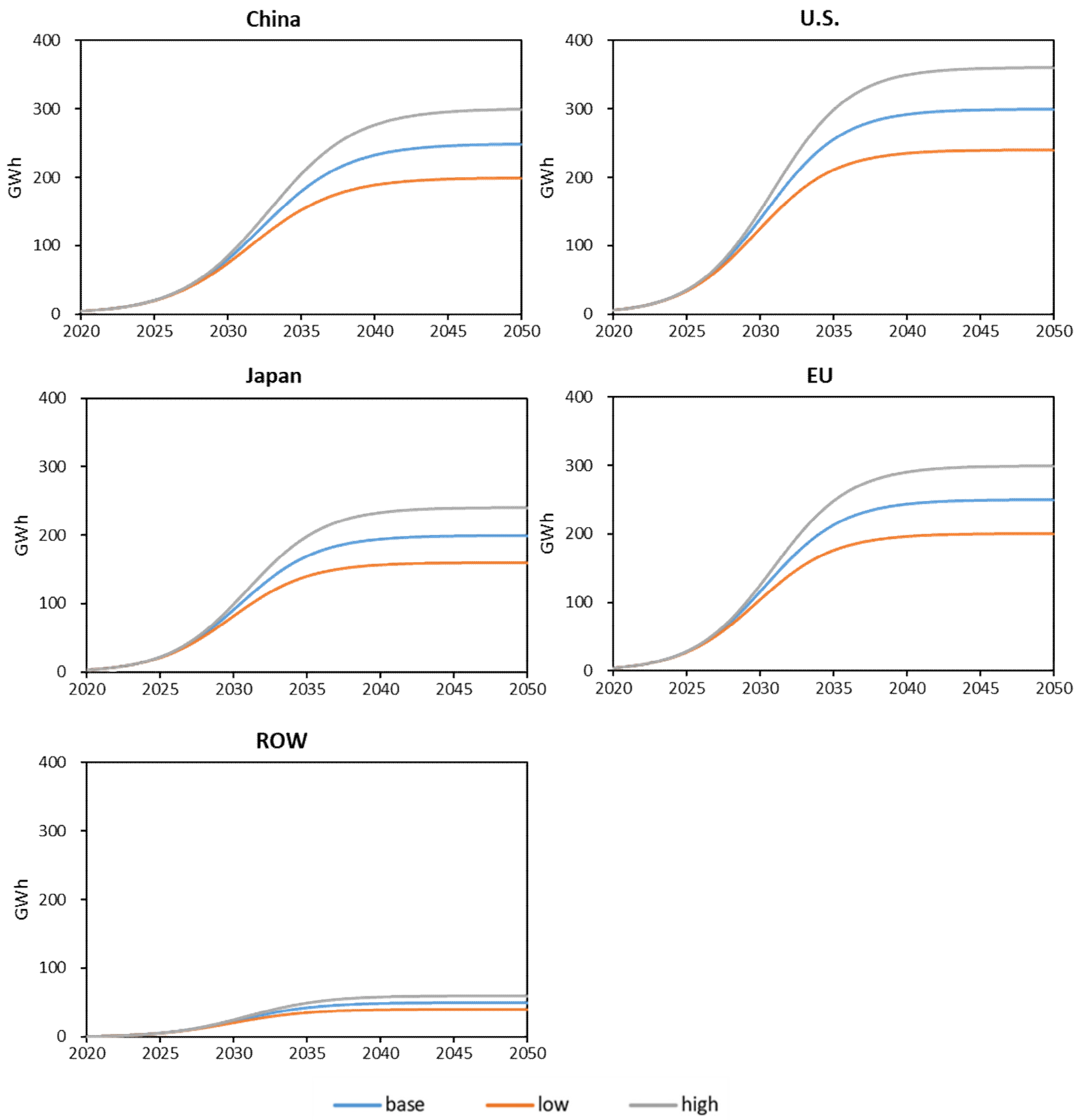

Supplementary Figure 5 ESS stock and variation range per region from 2020 to 2050.

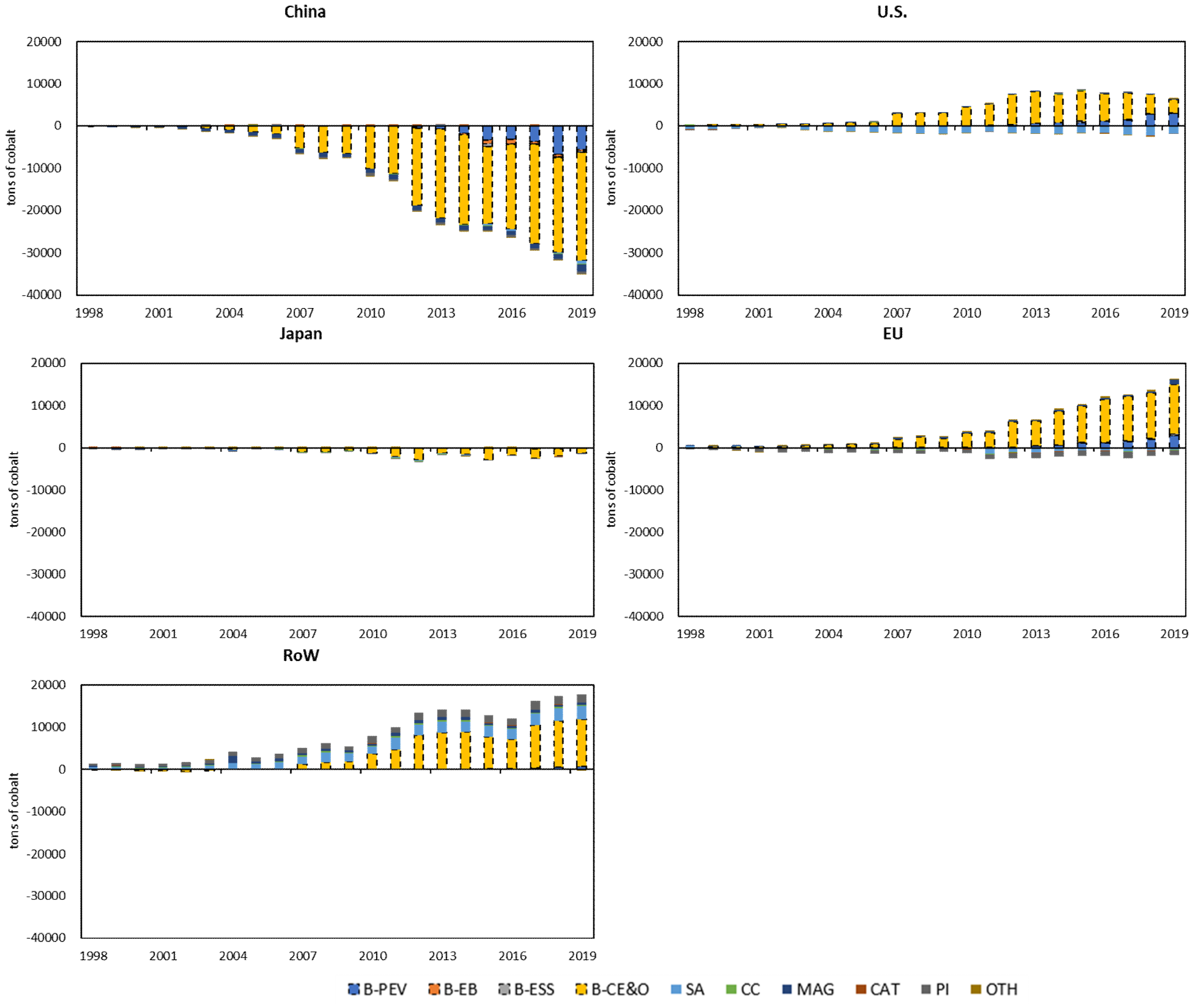

Supplementary Figure 20 Net import of cobalt by end-uses during 1998-2019 among the five regions. Net import denotes the difference between import flow and export flow. The positive value represents that the country is a net importer of this end-use. And the negative value stands for more exports. The bars of battery end uses are portrayed in dashed lines, owing to the inability to distinguish between the trade data for different types of battery products, thereby the net imports of four battery products are calculated by the share of their corresponding cobalt demand.

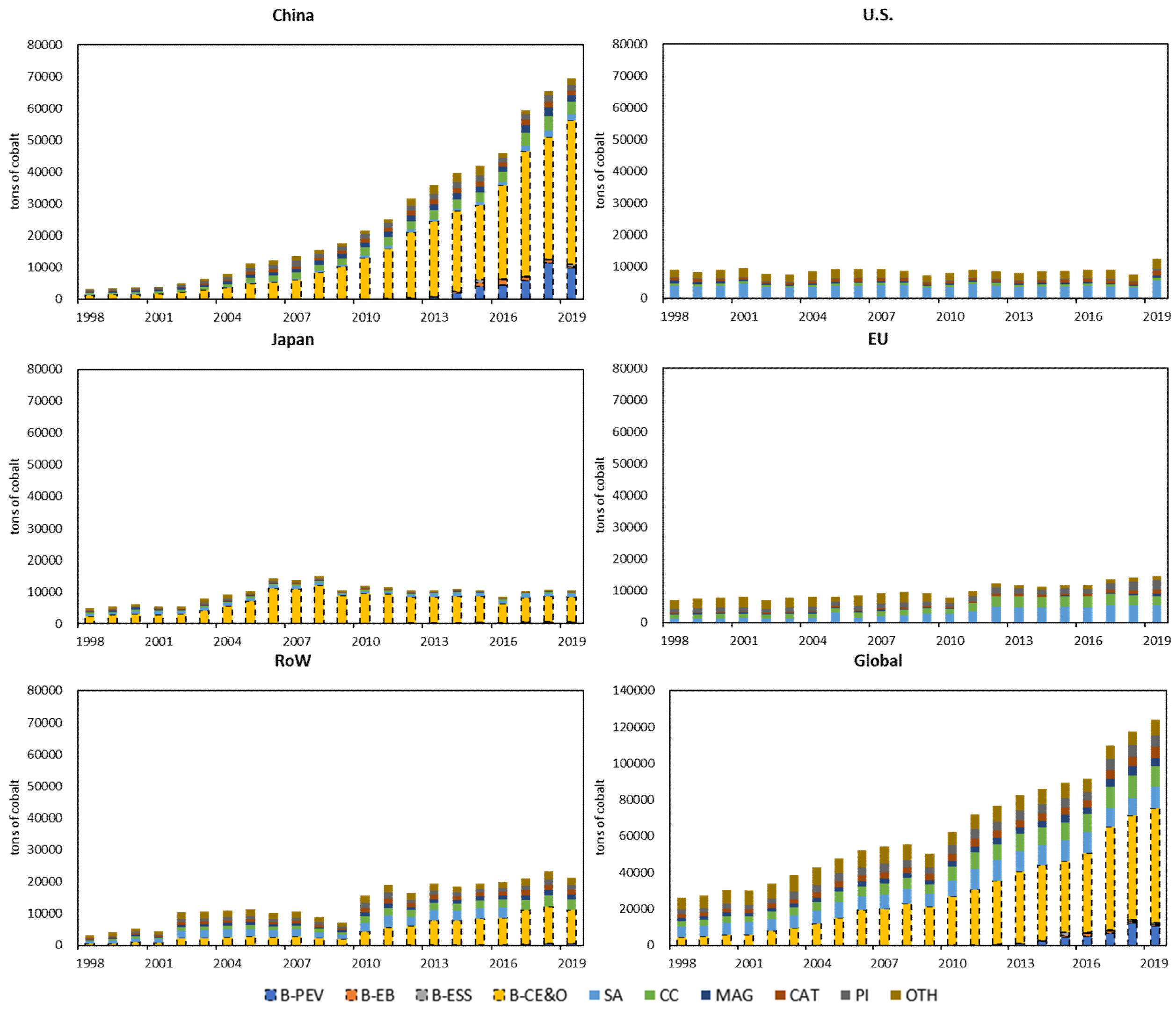

Supplementary Figure 21 Historical cobalt apparent consumption by end uses during 1998-2019 among the five regions. The bars of battery end uses are portrayed in dashed lines to indicate the further bottom-up quantification of different battery applications due to data deficiency.

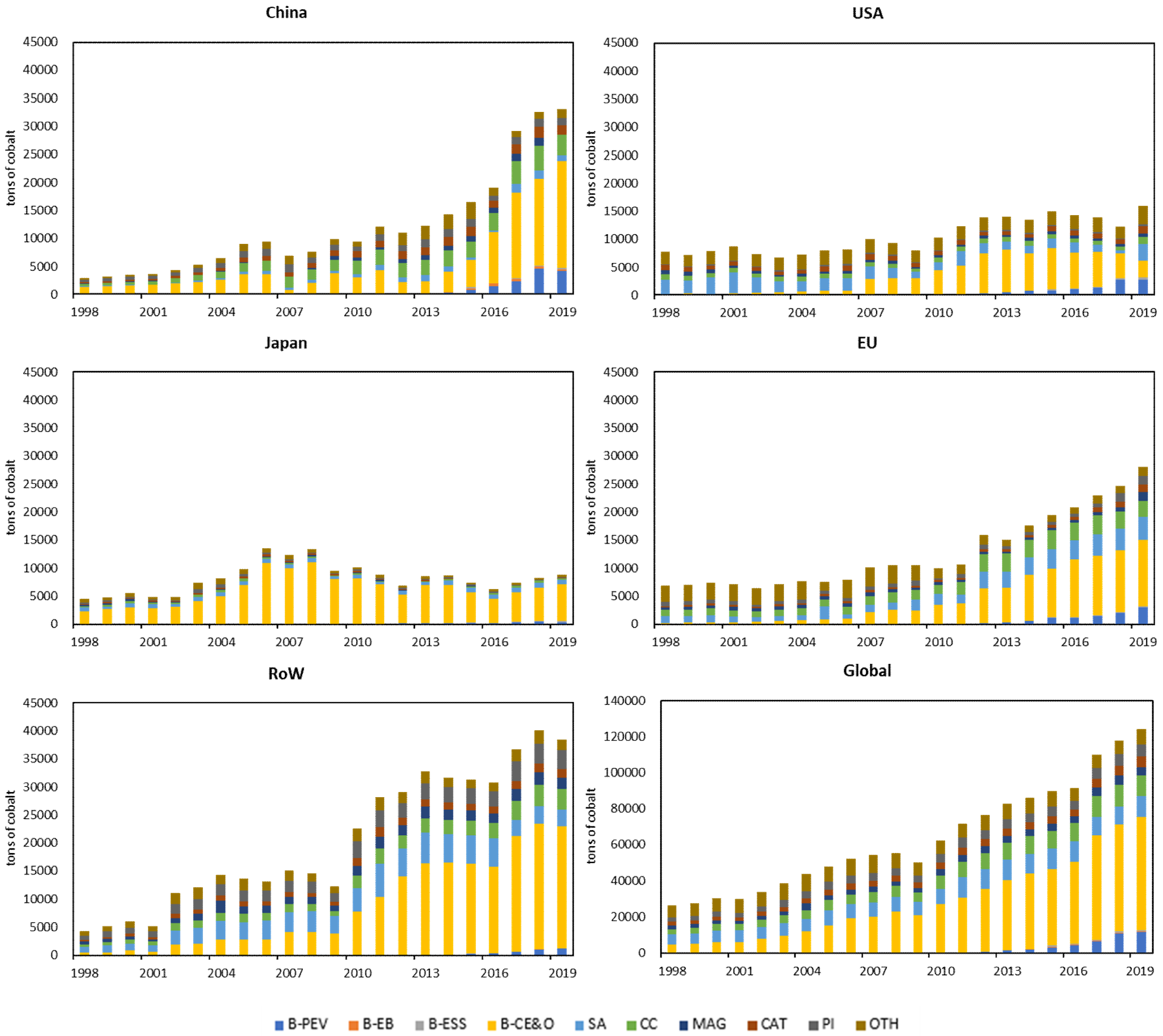

Supplementary Figure 22 Historical cobalt demand by end uses during 1998-2019 among different regions. The cobalt demand for B-PEV, B-EB, and B-ESS are calculated by a bottom-up approach, for traditional uses including all types of battery are calculated by the top-down method. The cobalt demand for B-CE&O is the difference between all types of battery and three emerging battery end uses.

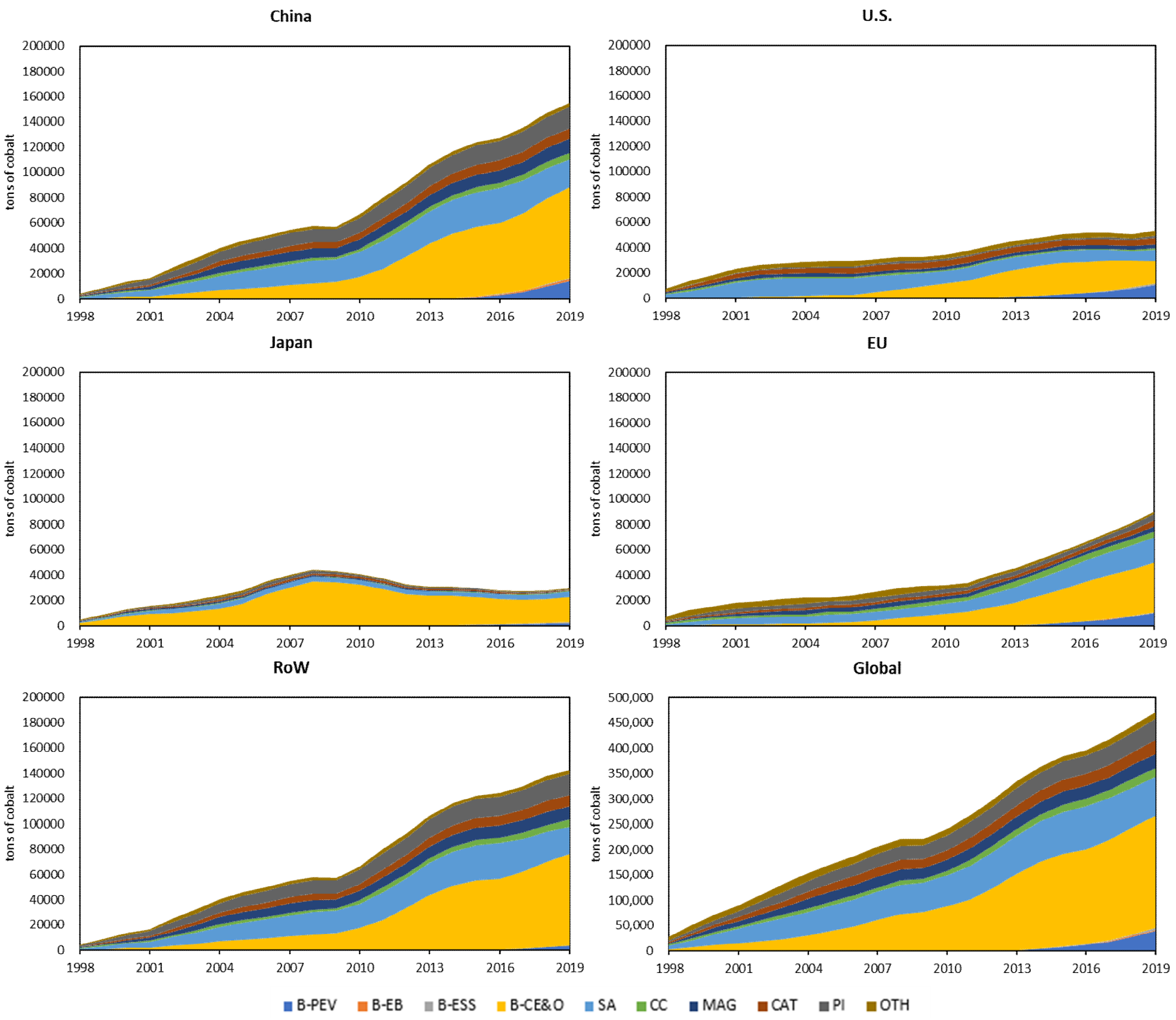

Supplementary Figure 23 Historical cobalt in-use stock by end uses during 1998-2019 among different regions.

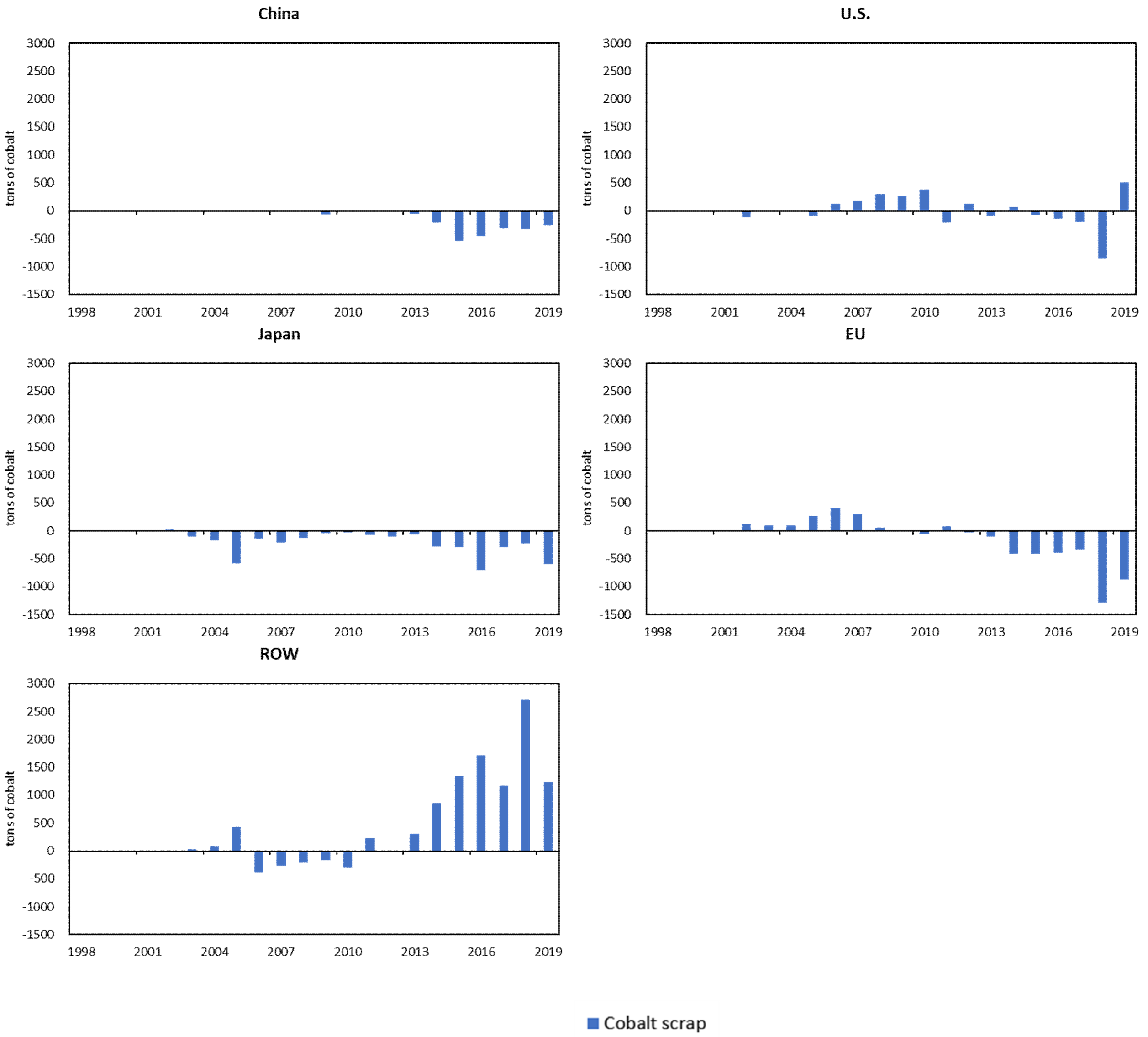

Supplementary Figure 24 Net import of cobalt content for cobalt scrap during 1998-2019 among the five regions. Net import denotes the difference between import flow and export flow, and the positive value represents that the country is a net importer of this end-use, and the negative value stands for more exports.

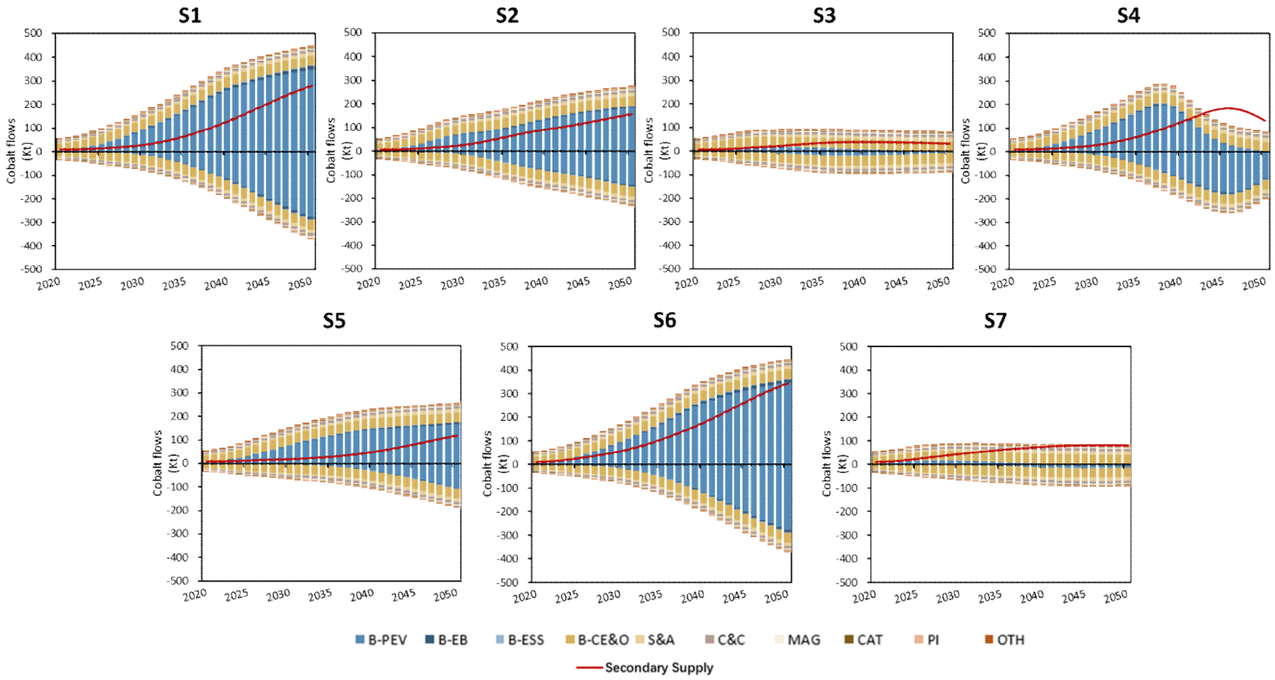

Supplementary Figure 25 Prospective cobalt inflow, outflow, and secondary supply of China under the seven scenarios from 2020 to 2050. The positive values represent cobalt inflows and negative values represent cobalt outflows.

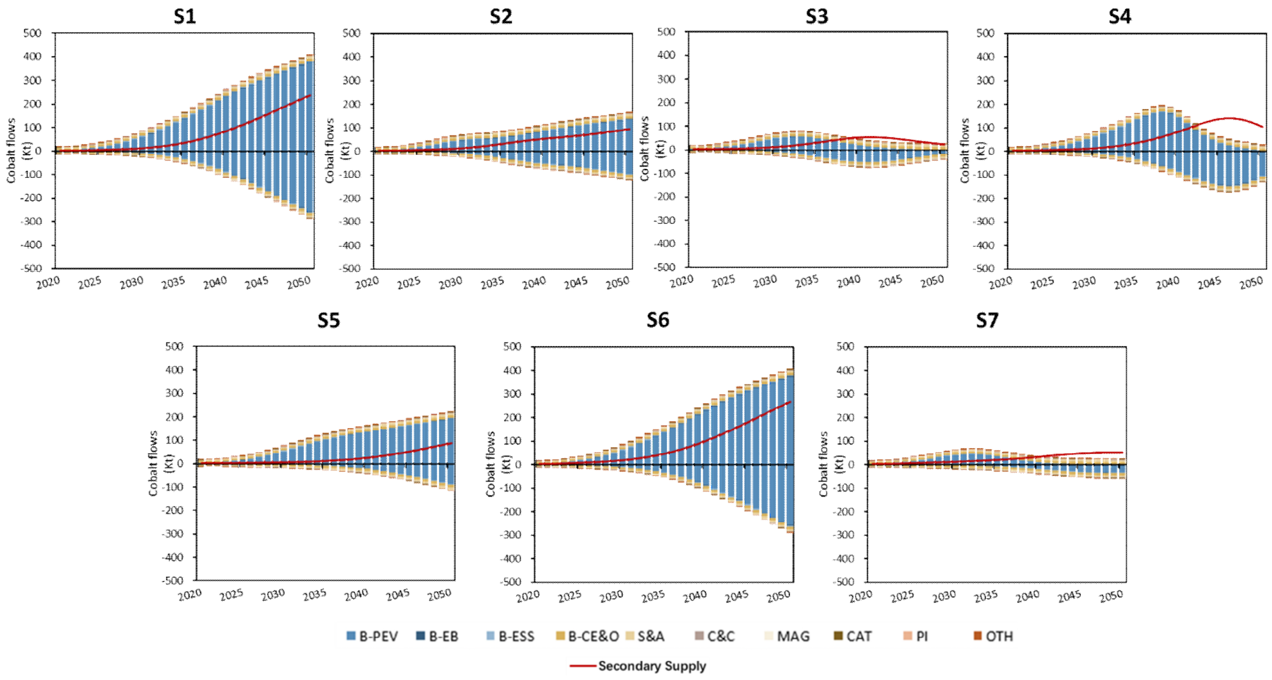

Supplementary Figure 6 Prospective cobalt inflow, outflow, and secondary supply of the U.S. under seven scenarios from 2020 to 2050. The positive values represent cobalt inflows and negative values represent cobalt outflows.

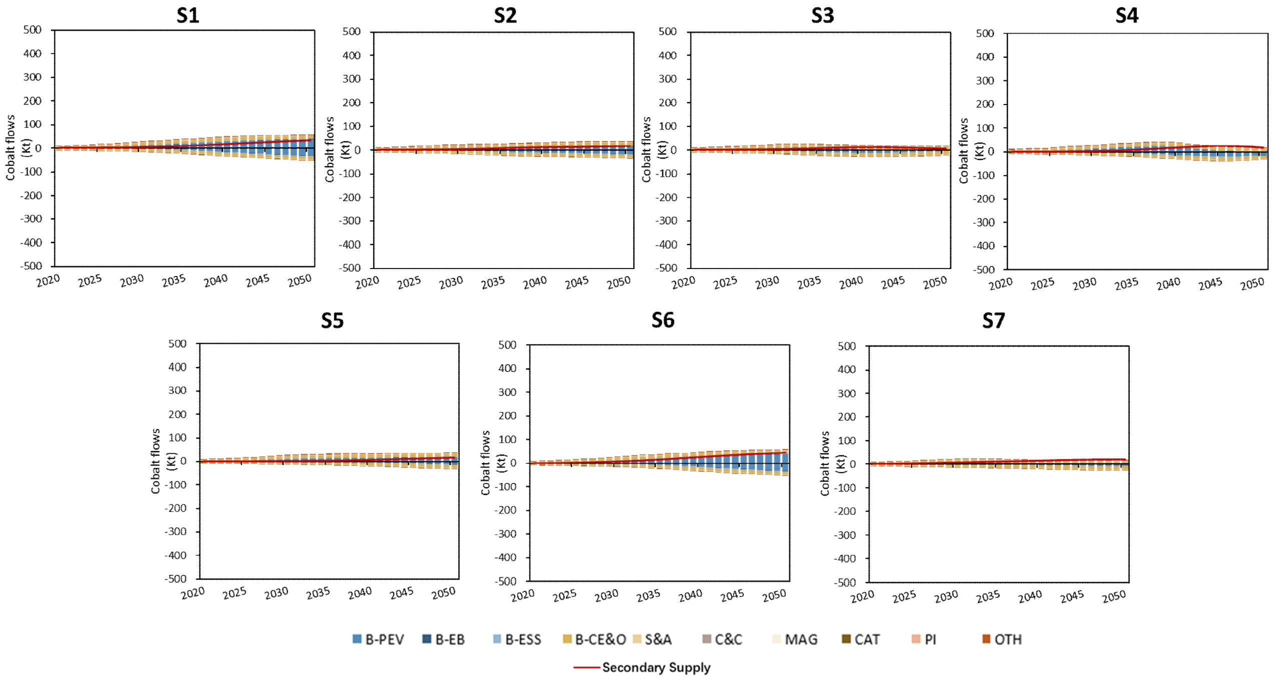

Supplementary Figure 7 Prospective cobalt inflow, outflow, and secondary supply of Japan under seven scenarios from 2020 to 2050. The positive values represent cobalt inflows and negative values represent cobalt outflows.

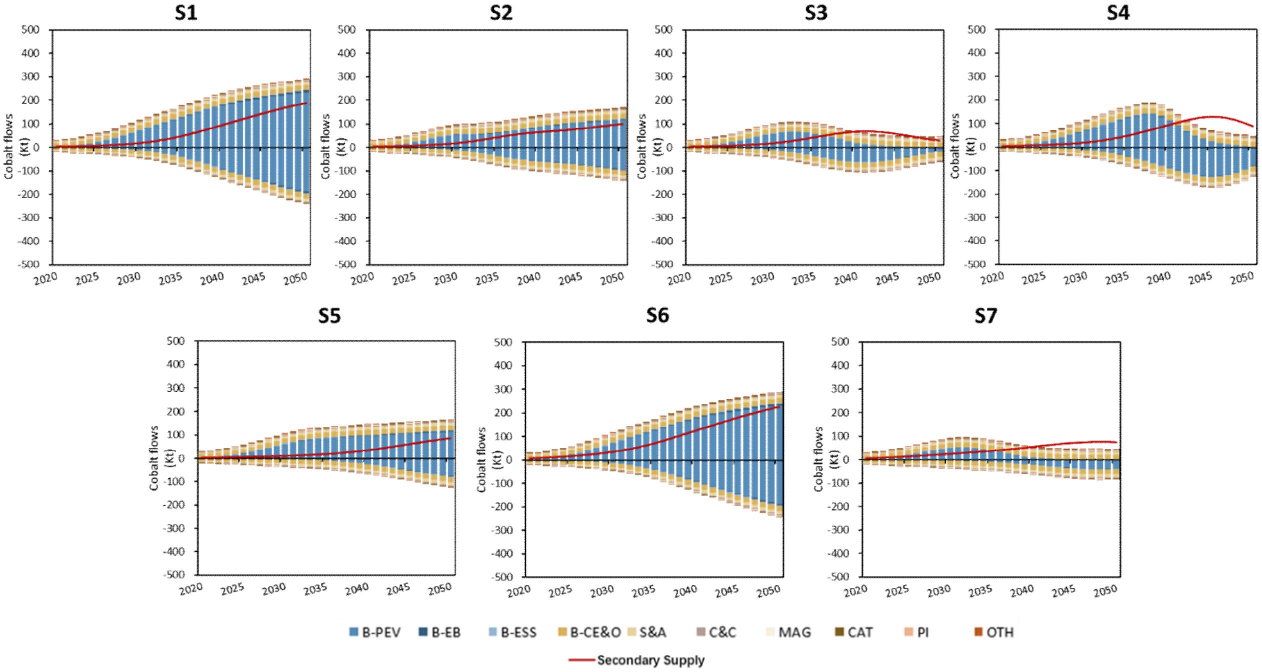

Supplementary Figure 8 Prospective cobalt inflow, outflow, and secondary supply of the EU under seven scenarios from 2020 to 2050. The positive values represent cobalt inflows and negative values represent cobalt outflows.

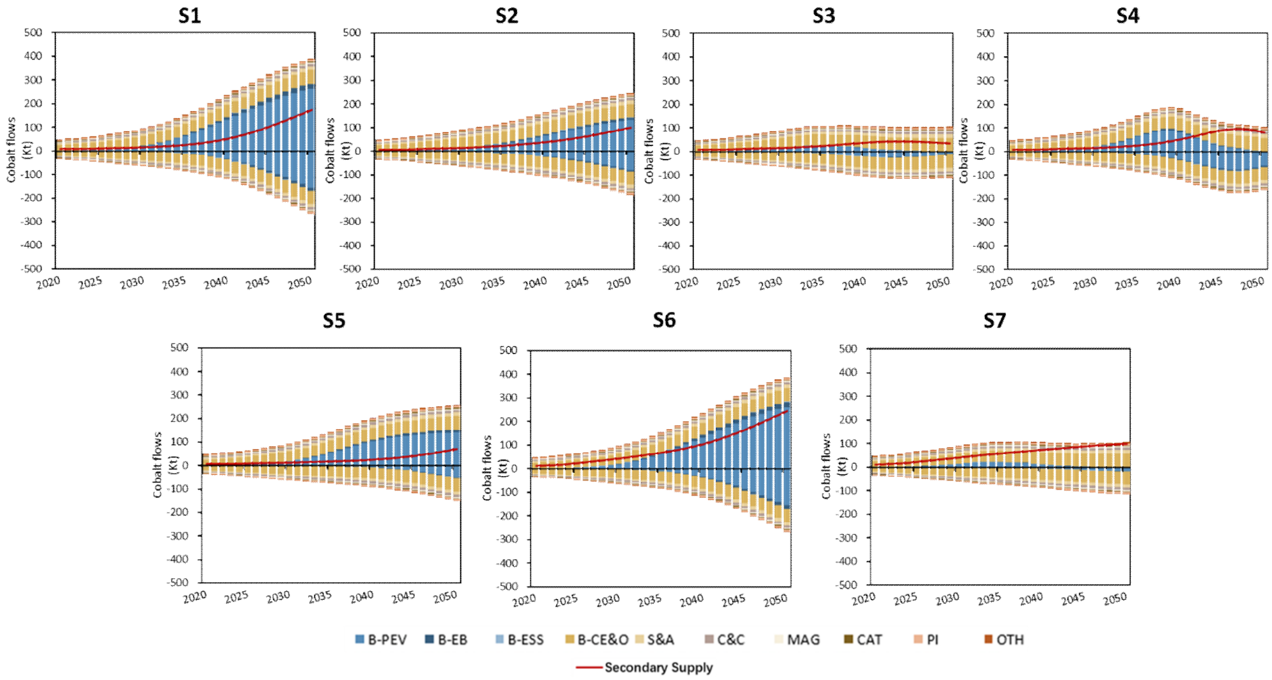

Supplementary Figure 9 Prospective cobalt inflow, outflow, and secondary supply of the ROW under seven scenarios from 2020 to 2050. The positive values represent cobalt inflows and negative values represent cobalt outflows.

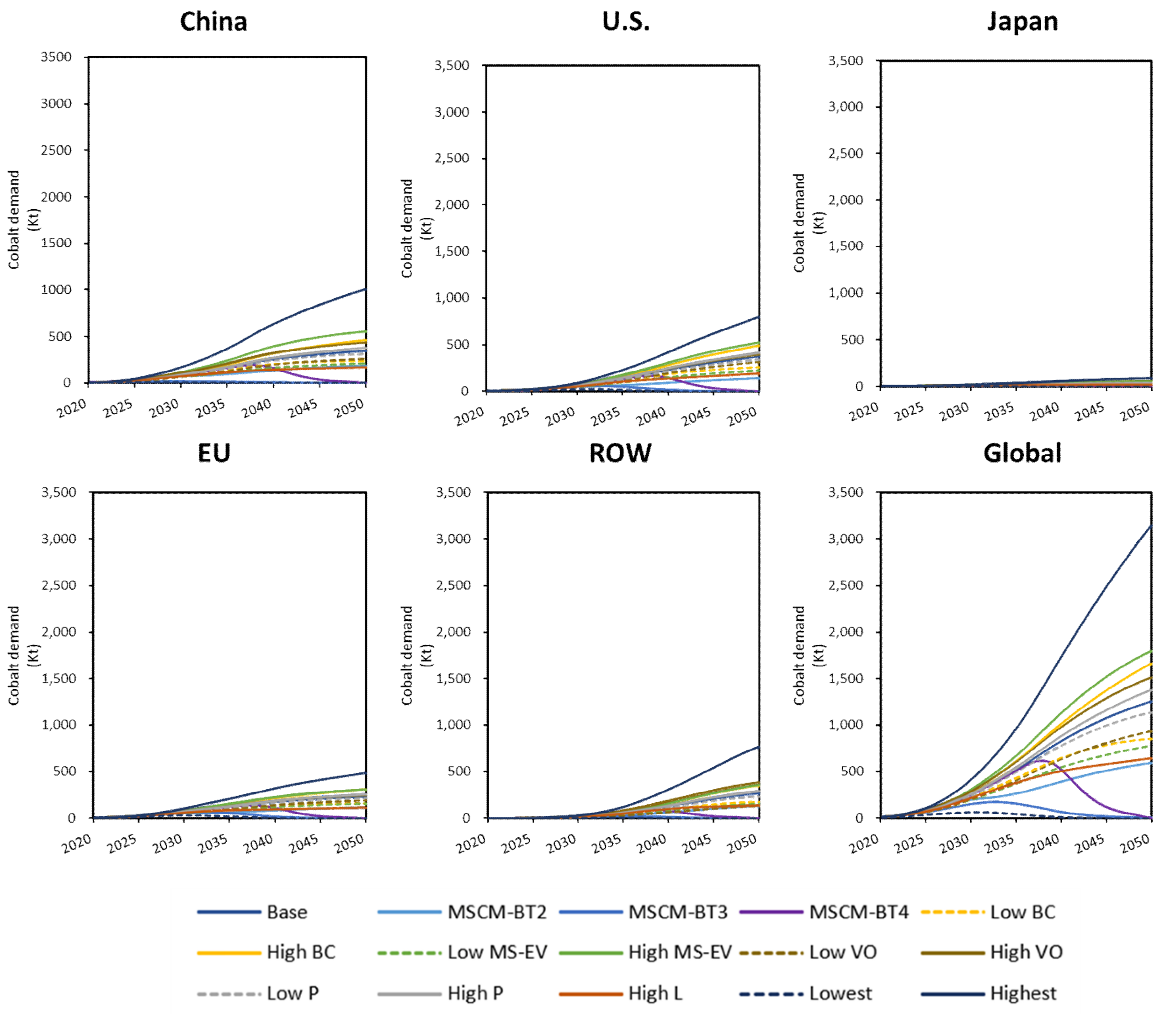

Supplementary Figure 30 Prospective regional and global cobalt demand for B-PEV by 2050, with absolute sensitivities by altering only one parameter at one time. MSCM-BT2: battery cathode material market shares in low-cobalt battery cathode technology scenario; MSCM-BT3: battery cathode material market shares in LFP-dominant battery cathode technology scenario; MSCM-BT4: battery cathode material market shares in next-generation cobalt-free battery cathode technology scenario; BC: battery capacity; MS-EV: market share of passenger electric vehicles; VO: passenger vehicle ownership; P: population; L: battery lifetime. Base (highest/lowest) scenario means all the key parameters are set in base (high/low) value.

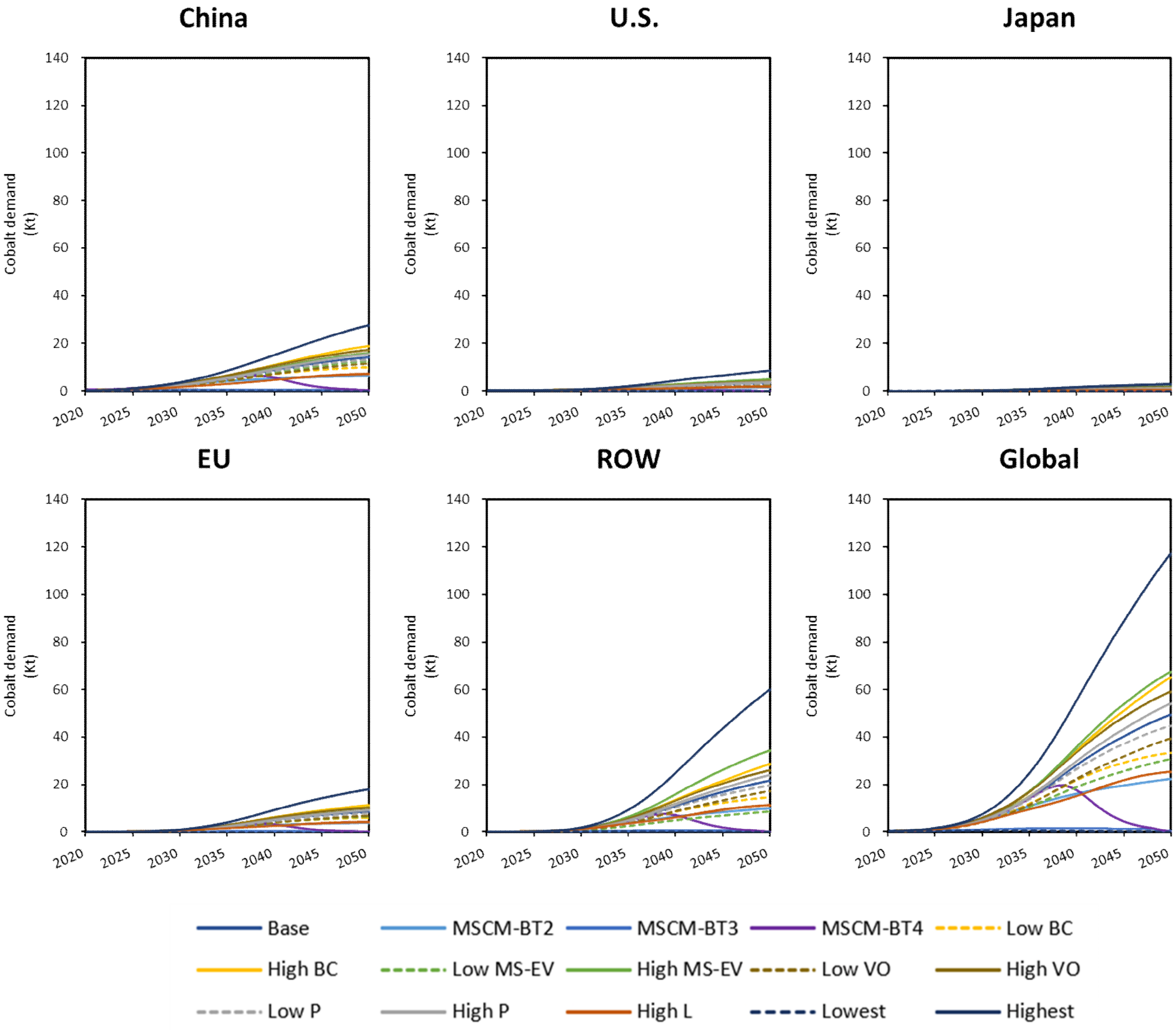

Supplementary Figure 31 Prospective regional and global cobalt demand for B-EB by 2050, with absolute sensitivities by altering only one parameter at one time.

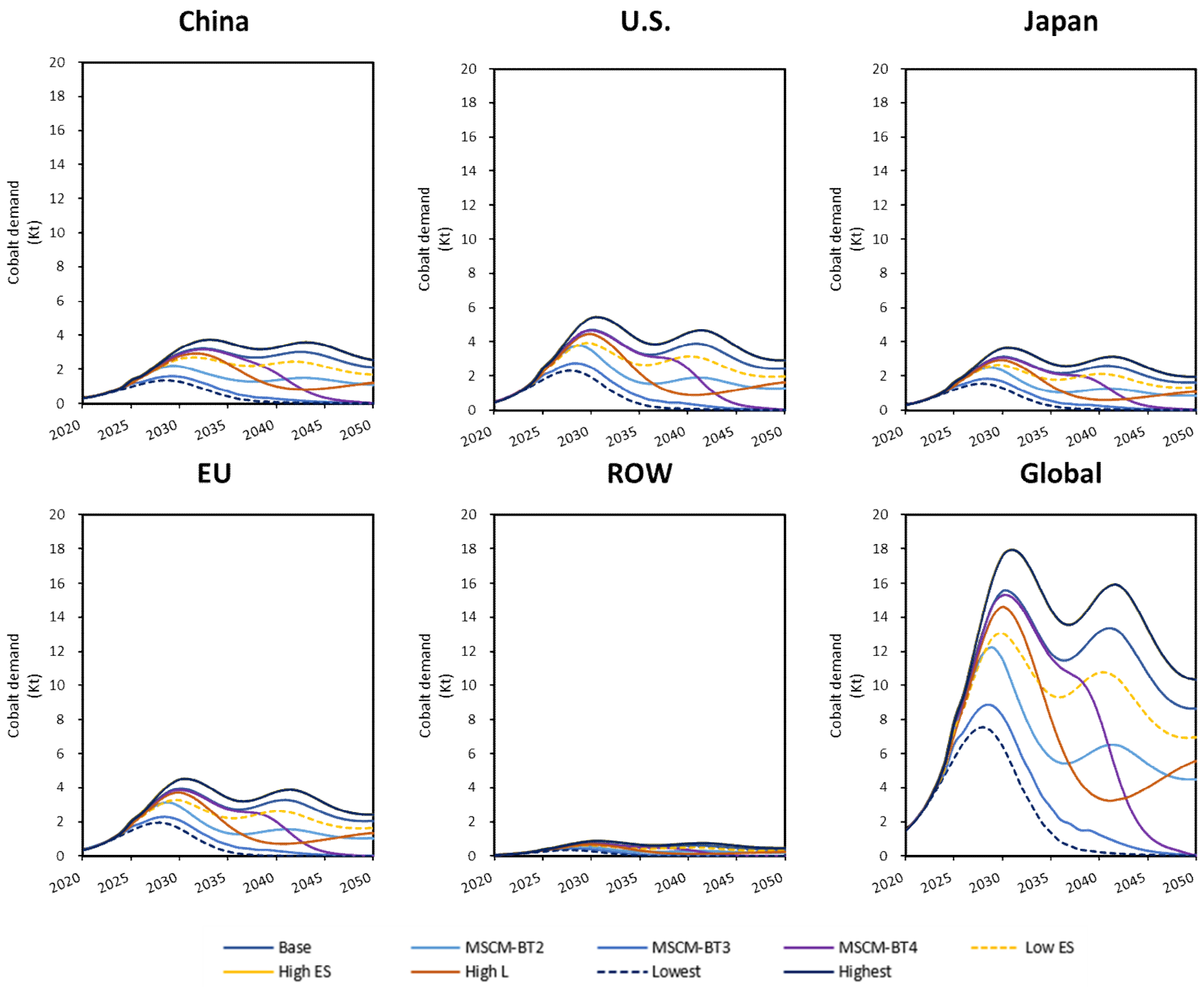

Supplementary Figure 32 Prospective regional and global cobalt demand for B-ESS by 2050, with absolute sensitivities by altering only one parameter at one time. ES: ESS stock.

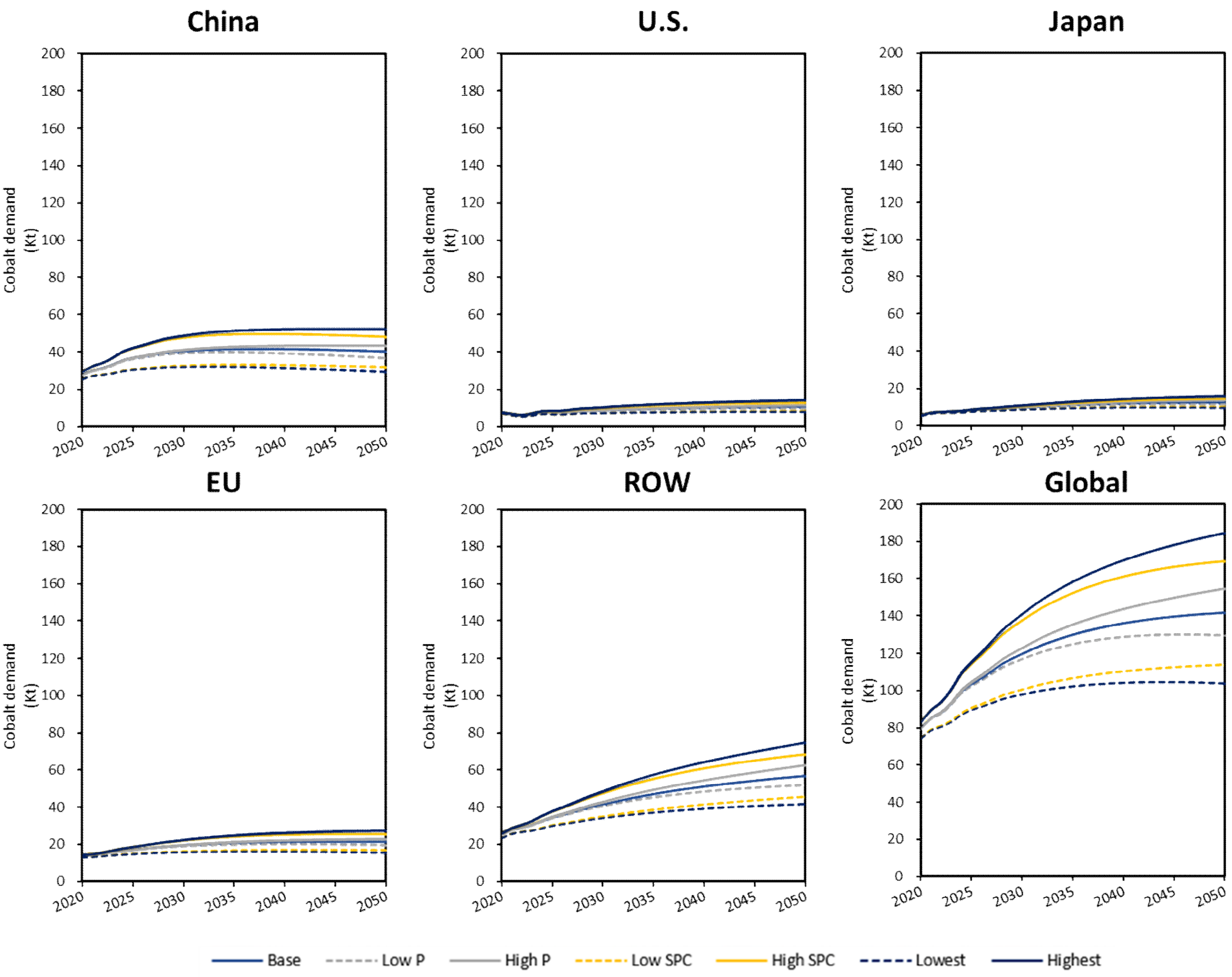

Supplementary Figure 33 Prospective regional and global cobalt demand for B-CE&O by 2050, with absolute sensitivities by altering only one parameter at one time. SPC: cobalt stock per capita.

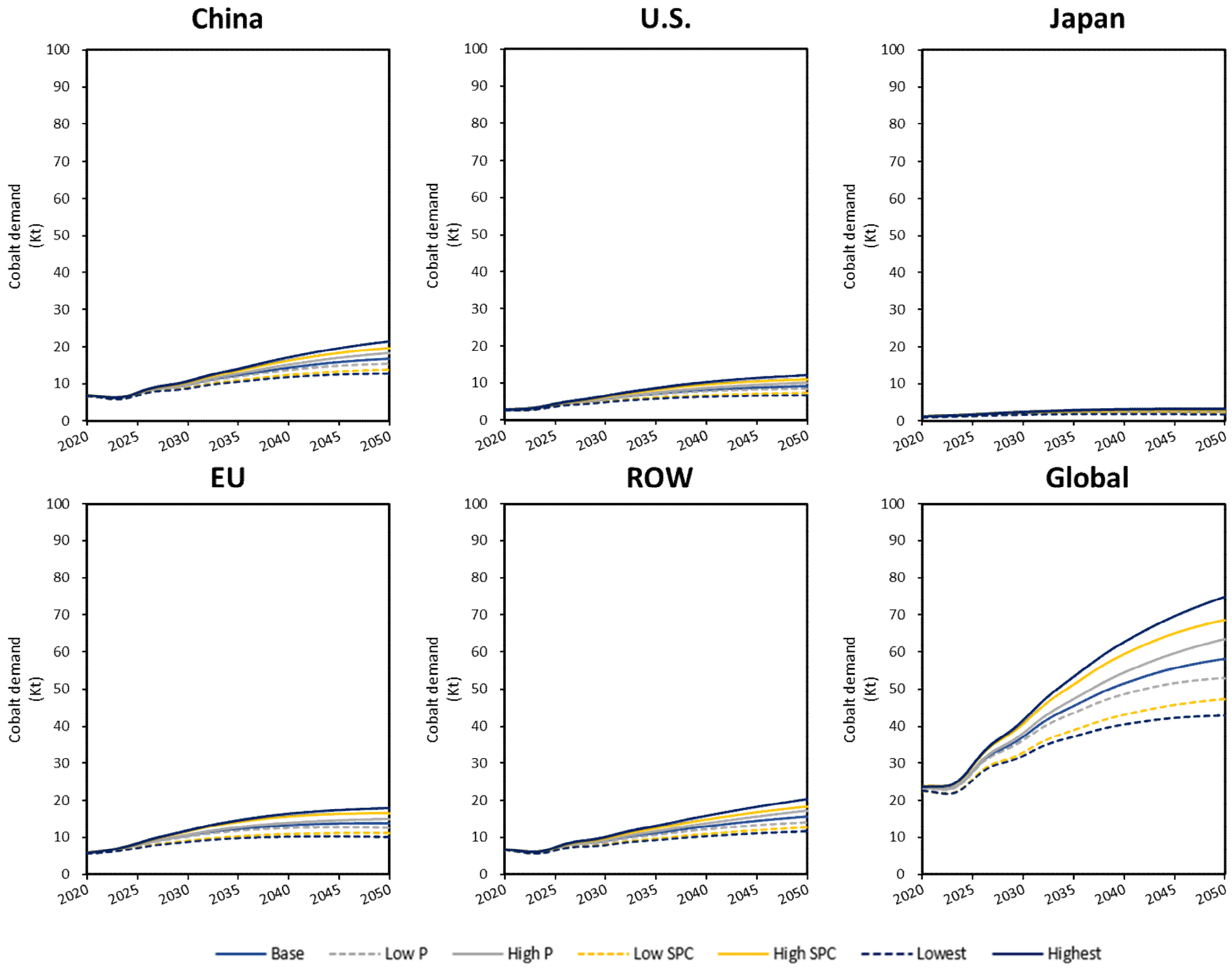

Supplementary Figure 34 Prospective regional and global cobalt demand for SA by 2050, with absolute sensitivities by altering only one parameter at one time.

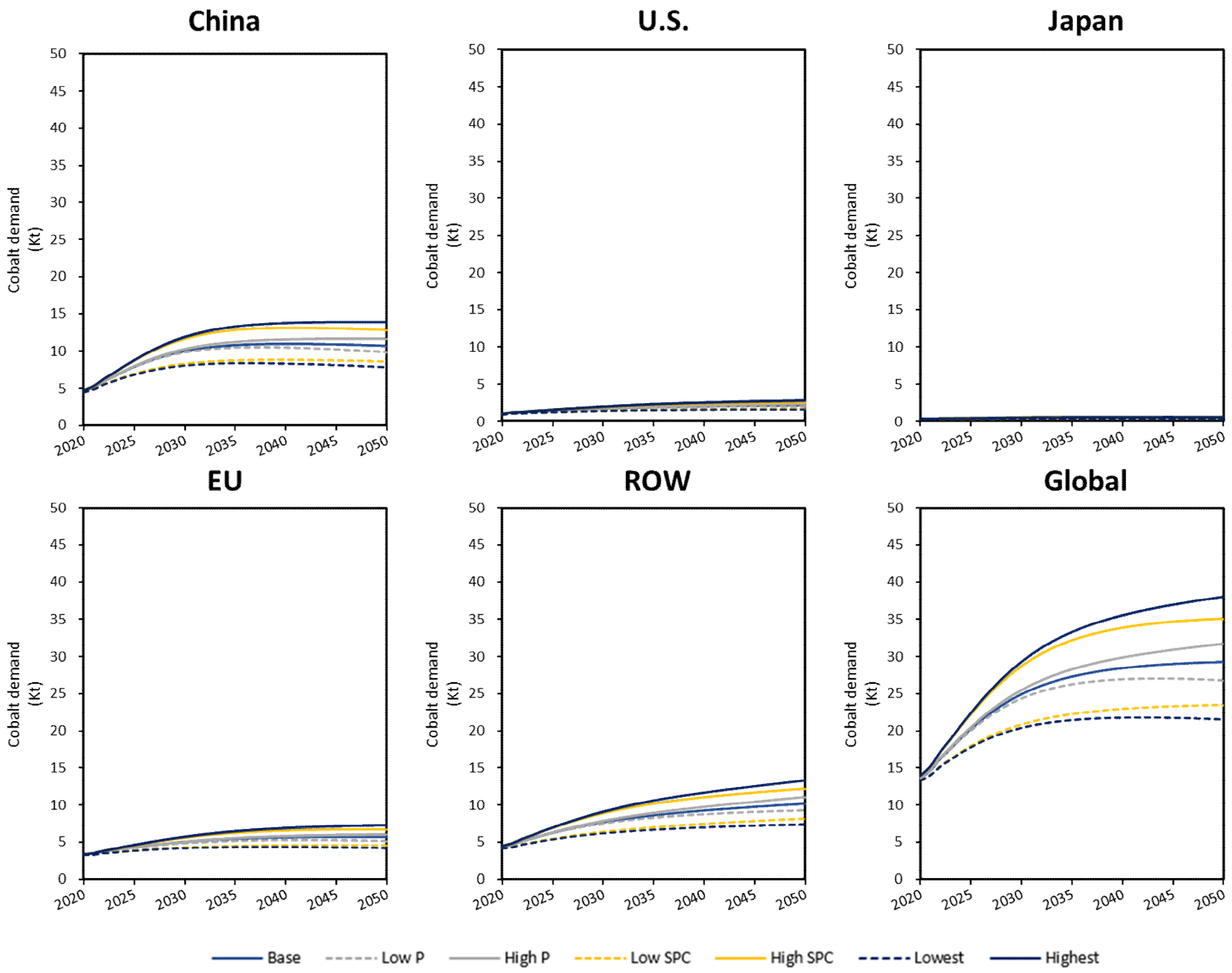

Supplementary Figure 35 Prospective regional and global cobalt demand for CC by 2050, with absolute sensitivities by altering only one parameter at one time.

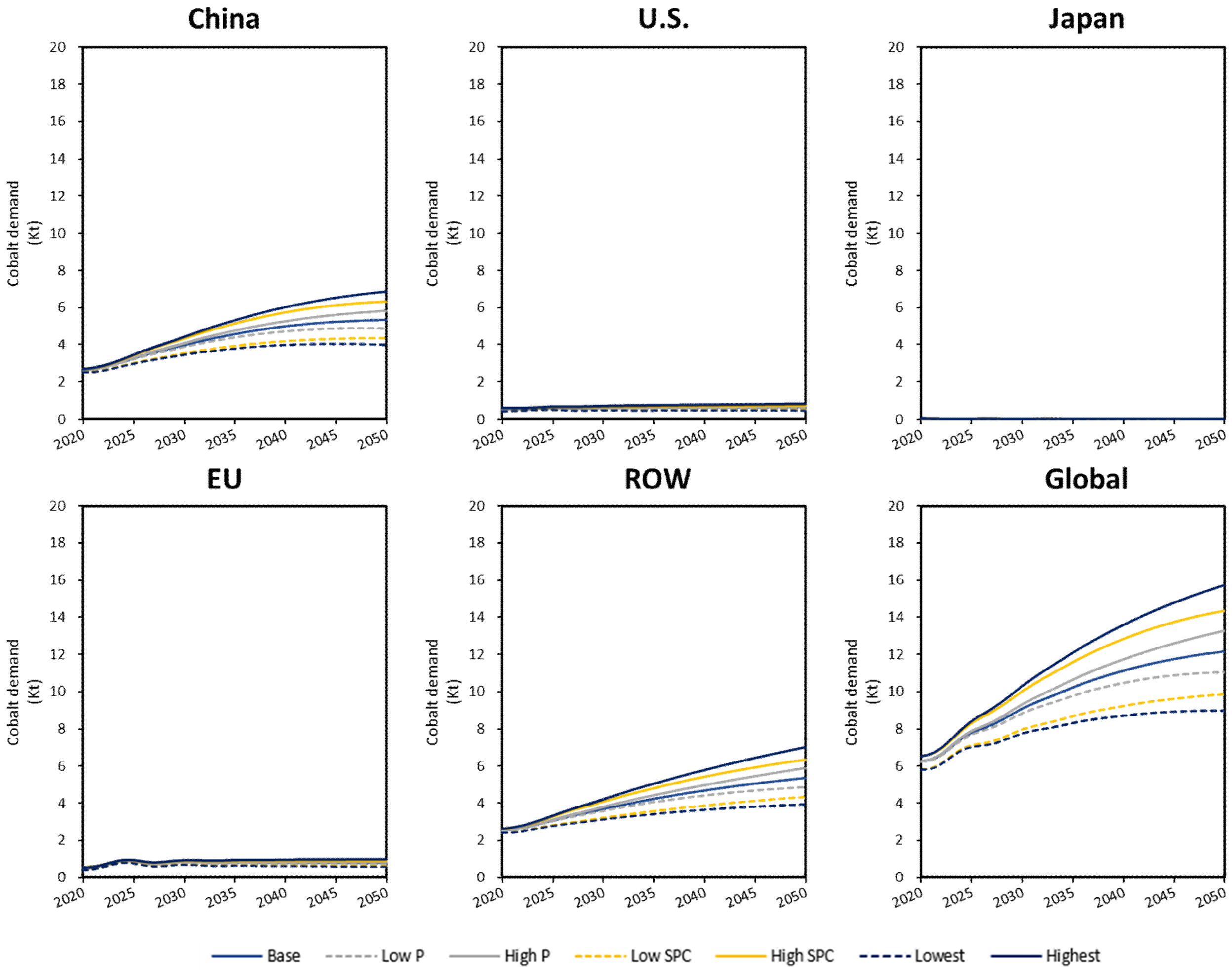

Supplementary Figure 10 Prospective regional and global cobalt demand for MAG by 2050, with absolute sensitivities by altering only one parameter at one time.

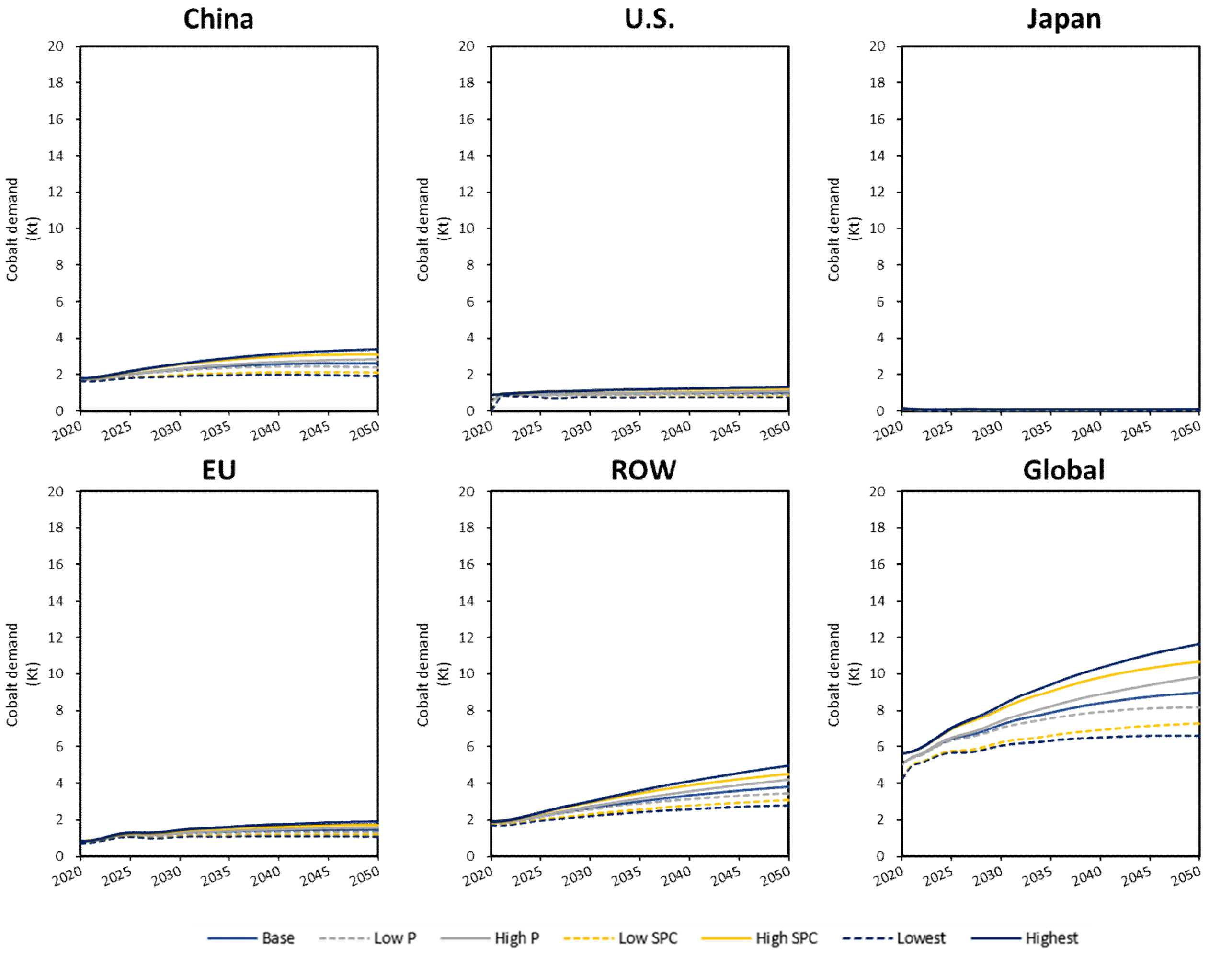

Supplementary Figure 37 Prospective regional and global cobalt demand for CAT by 2050, with absolute sensitivities by altering only one parameter at one time.

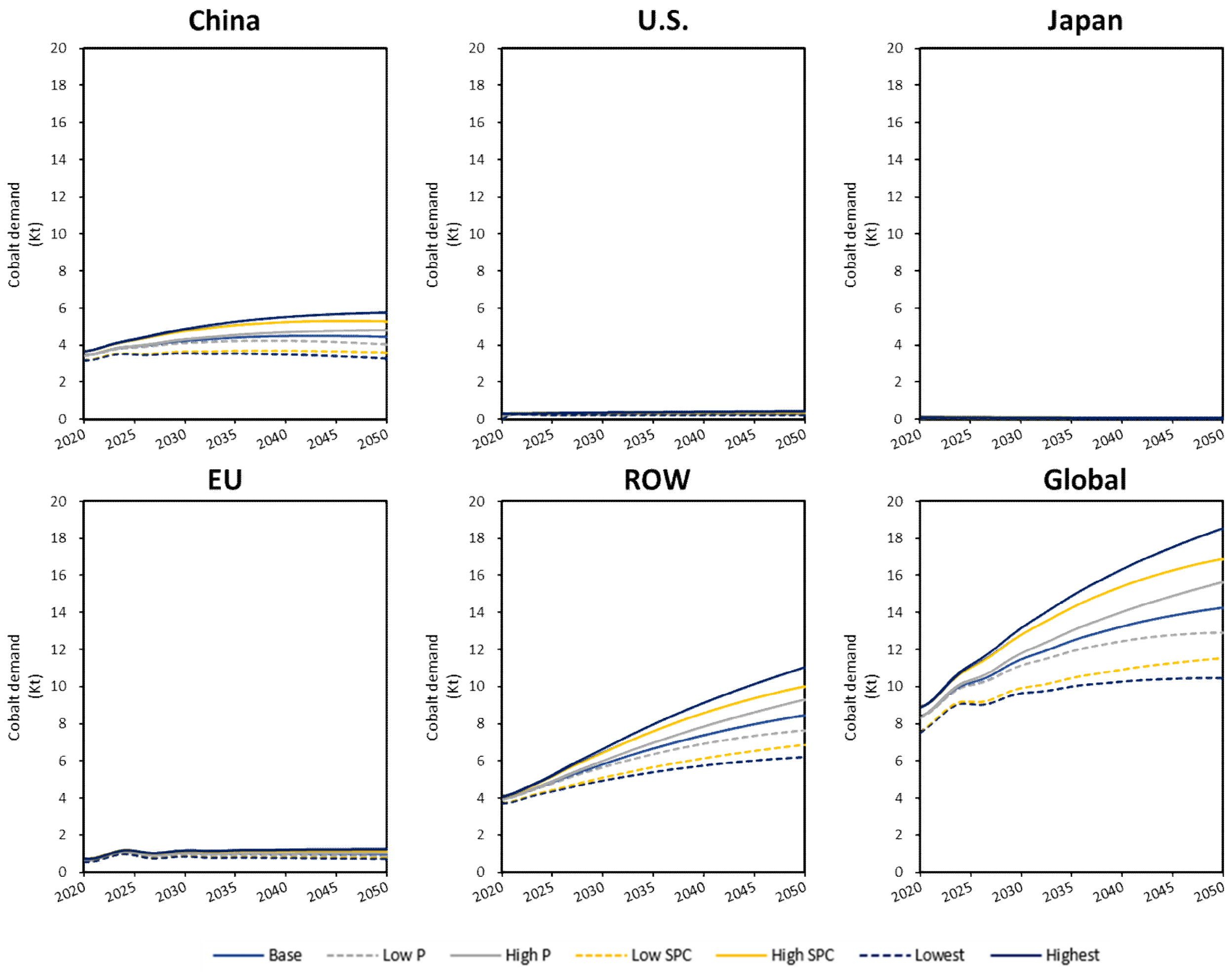

Supplementary Figure 11 Prospective regional and global cobalt demand for PI by 2050, with absolute sensitivities by altering only one parameter at one time.

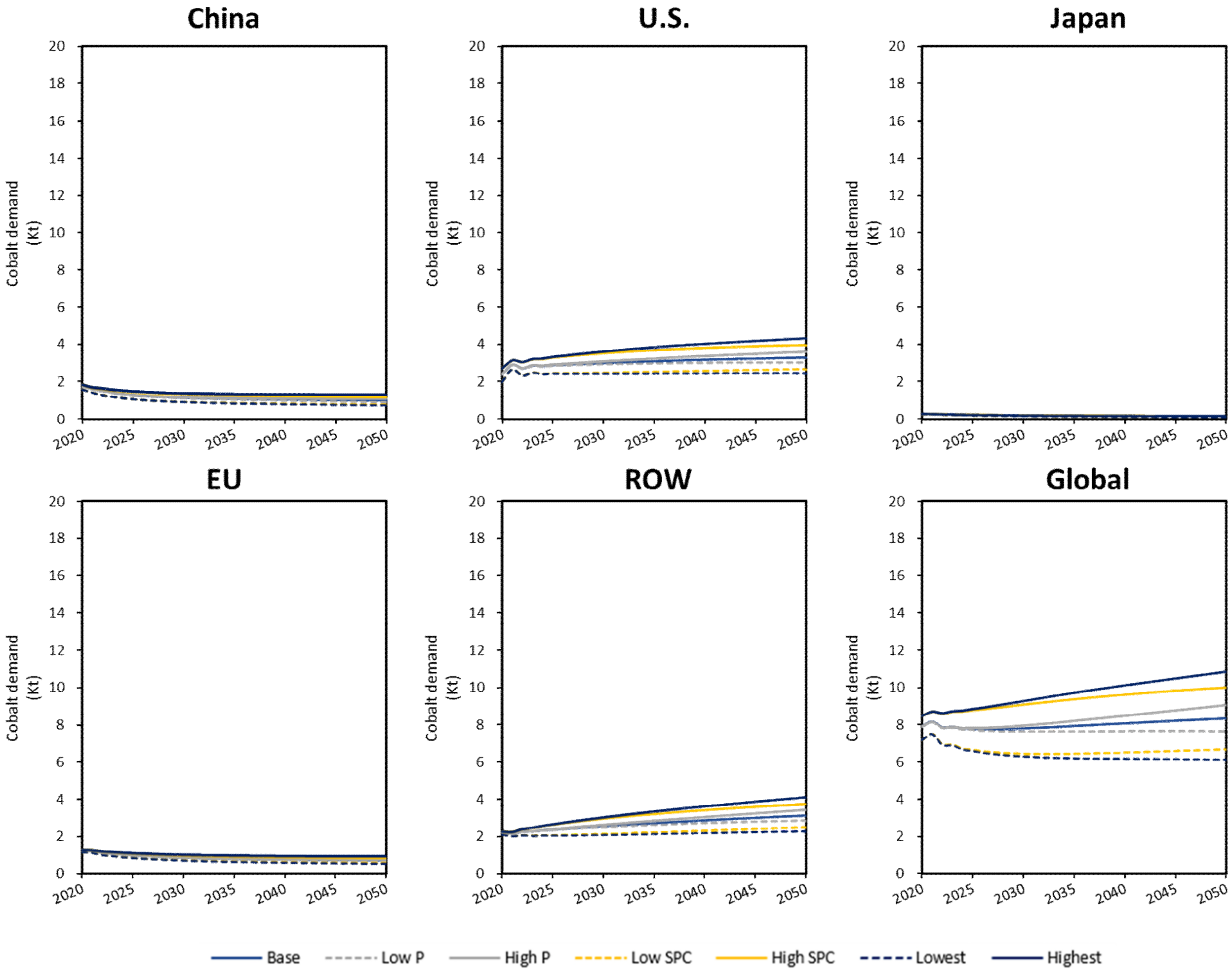

Supplementary Figure 12 Prospective regional and global cobalt demand for OTH by 2050, with absolute sensitivities by altering only one parameter at one time.

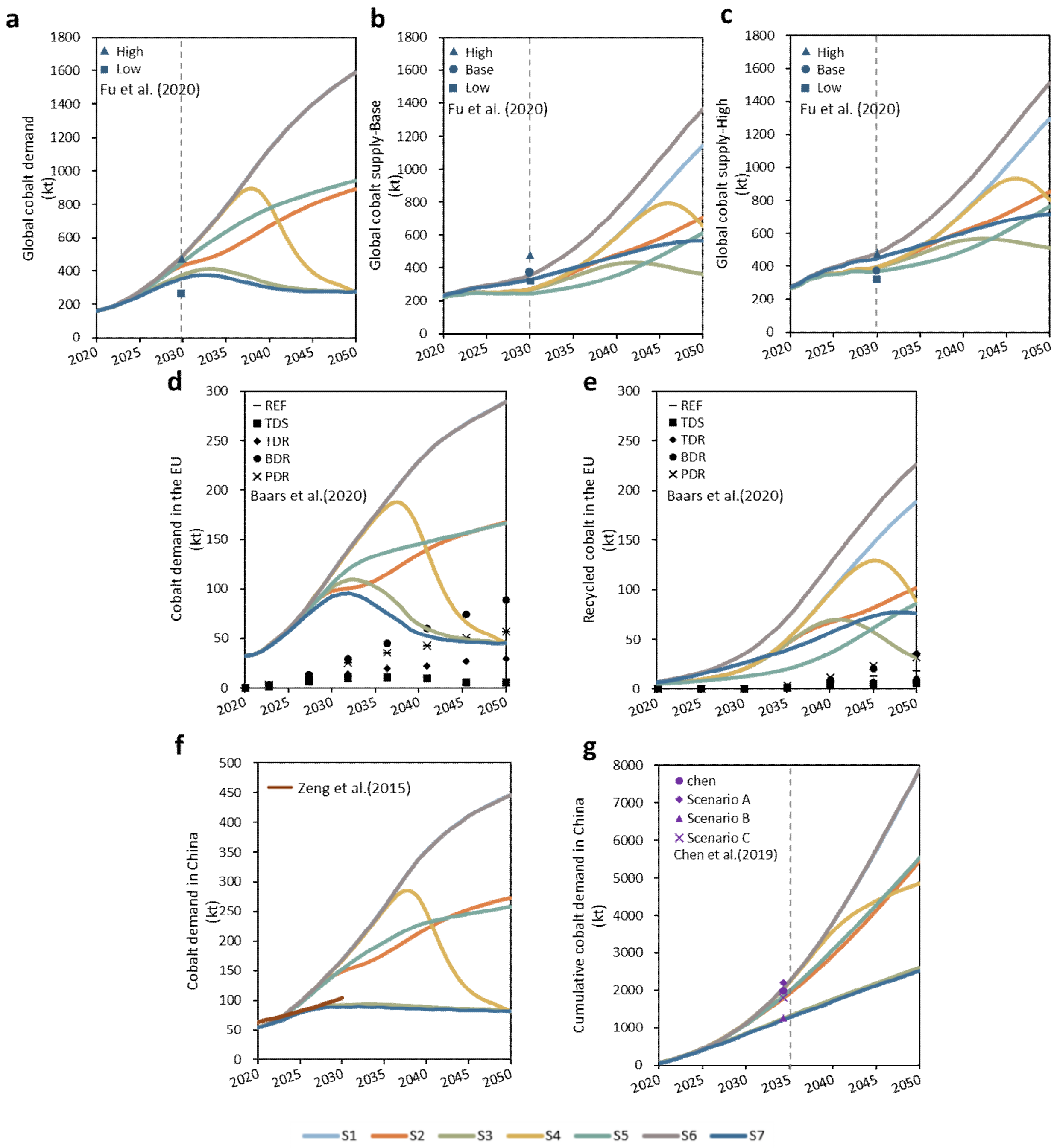

Supplementary Figure 40 Comparison of scenario results with previous studies: (a) Global cobalt demand scenarios compared with Fu et al. (2020), **(b)** Global cobalt supply scenarios (primary-base+secondary) compared with Fu et al. (2020), **(c)** Global cobalt supply scenarios (primary-high+secondary) compared with Fu et al. (2020), **(d)** Cobalt demand scenarios of EU compared with Baars et al. (2020), **(e)** Recycled cobalt scenarios of EU compared with Baars et al. (2020), **(f)** Cobalt demand scenarios of China compared with Zeng et al. (2015), **(g)** Cumulative cobalt demand scenarios of China compared with Chen et al. (2019).

Supplementary Note 1 System definition.

The global anthropogenic cobalt cycle (as shown in Supplementary Figure 1) includes five transformation processes: mining, refining, manufacturing, use, and waste management & recycling process. The cobalt flows into the refining process could be derived from three sources: cobalt ores produced from mining (primary cobalt supply), cobalt old scrap recycled from end-of-life (EoL) cobalt-containing final products (secondary cobalt supply), and net import of cobalt old scrap. The cobalt new scrap generated in the manufacturing process will be partly recycled and reentered again and the unrecycled scrap is counted as generated waste. The cobalt-containing final products could be classified into three emerging end uses and seven traditional end uses. Emerging end uses include batteries for passenger electric vehicles (B-PEV), batteries for electric buses (B-EB), batteries for energy storage systems (B-ESS). Traditional end uses include batteries for consumer electronics and others (B-CE&O), superalloys (SA), cemented carbides (CC), magnets (MAG), catalysts (CAT), pigments (PI), and other uses (OTH). Losses existing in mining, refining, manufacturing, and waste management & recycling processes are calculated by multiplying relevant coefficients (see Supplementary Table 5). The flows and in-use stocks of manufacturing, use, and waste management & recycling are characterized by five regions: China, the U.S., Japan, the EU, and ROW with higher resolution. The trade of cobalt-containing final products and cobalt waste and scrap between those five regions are also taken into consideration.

Supplementary Note 2 Trade data processing.

The trade data of cobalt-containing final products and cobalt waste come from the United Nations Comtrade Database[23]. We have considered 67 commodities as shown in Supplementary Table 1, which are compiled from the previous literatures[4,58] given the significance of the commodity and the value of trade volume and cobalt intensity. Radioactive cobalt-60, used as a radiation source mainly applied in the medical industry, is ignored in this paper because of the small trade volume[25]. Batteries of HEV are not considered for two reasons: Firstly, their battery mass is only 18.6kg per vehicle and far smaller than PHEV (88.9kg per vehicle) and BEV (210kg per vehicle)[3]. Secondly, the batteries contained in HEV are mostly NiMH battery (98%) which has low cobalt intensity (approximately 0.9 grams cobalt per battery), and lithium-ion battery only accounts for a small fraction (2%). Therefore, HEV will be slightly affected by LIB technology innovation[59] (in our scenario setting). The commodity lists are further categorized into seven end uses. All battery applications are regarded as one category. For example, as for the end use category ‘superalloys’, the related commodities include aircraft gas turbine engines as well as airplanes and helicopters.

The main problems of the trade data from UN Comtrade, such as data inconsistency, unit inconsistency, and outliers, were solved following the method in previous literature[60–62]. Specific descriptions regarding the study are listed as follows:

1) Data inconsistency. A trade flow can be reported twice as import and export by switching the status of reporter and partner, and the figure should be the same theoretically while often inconsistent owing to customs declaration discrepancy. We adopt import data as trade flows for it is deemed to be more credible in the previous studies[60]. And the export flow could also be obtained.
2) Unit inconsistency. All the cobalt end-uses commodity trade flows are reported in monetary values, while a few lacks physical value (kg) and the number of items (units). The estimated “world average price” is applied to fill in vacancies in physical values by transferring monetary values into physical values to cover physical data gaps.
3) Outliers. We use the algorithm method in the previous study[60] to correct outlier automatically when the value is 10 times larger or smaller than its neighbors on both sides. As for the outliers occurring for successive years, we delete extreme physical values in certain years when calculating “world average price” and use monetary value divided by the new “world average price” to replace the outliers.

Supplementary Note 3 Simulation of prospective cobalt demand.

3.1 Emerging end uses

Technology-rich regional cobalt demand for emerging end uses are set separately based on a product-specific stock-driven model that consists of the product stock module and the material module. The prospective EVs inflows and ESS are simulated by a product-stock-driven model[63–65].

(1) B-PEV/B-EB

The prospective cobalt demand for B-PEV and B-EB ($CoDemand_{EV}$) of region k ($k \in \{China, U.S., Japan, EU, ROW\}$) in the year t was determined by cobalt intensity (CI), battery cathode material market share ($MSCM$), average battery capacity per vehicle (ABC), and battery demand for EV($EVBD$). The equations are as follows:

$$CoDemand_{EV}(k,t) = \sum_i (CI_{i,k,t} * MSCM_{i,k,t}) * ABC_{k,t} * EVBD_{k,t} \quad (1)$$

$$ABC_{j,k,t} = \sum_j (MS_{j,k,t} * BC_{j,k,t}) \quad (2)$$

$$EVBD_{k,t} = \begin{cases} VD_{k,t} * MSEV_{k,t} + VD_{k,t-T_B} * MSEV_{k,t-T_B} & if\ T_B = 1/2\,T_V \\ VD_{k,t} * MSEV_{k,t} & if\ T_B = T_V \end{cases} \quad (3)$$

where CI indicates cobalt intensity, $MSCM$ indicates battery cathode material market share, i stands for the type of battery cathode material. And the average battery capacity (ABC) of EV type j ($j \in \{BEV, PHEV\}$) is multiplied by the market share of each type in EV sales and their battery capacity (BC) per vehicle. Battery demand for EV ($EVBD$) depends on EV market share ($MSEV$), vehicle demand (VD) and the relationship between battery lifetime (T_B) and EV lifetime (T_V). The perspective vehicle demand (VD) is simulated by the stock-driven model, in conjunction with future vehicle stock (multiplying prospective vehicle ownership and population), average vehicle lifetime, and normal distribution.

(2) B-ESS

The cobalt demand projection for B-ESS is a bit different from B-PEV and B-EB. The cobalt future demand for B-ESS of region k ($k \in \{China, U.S., Japan, EU, ROW\}$) in the year t is affected by cobalt intensity (CI), battery cathode material market share ($MSCM$), and ESS demand ($ESSD$). The equation is as follows:

$$CoDemand_{ESS}(t) = \sum_i (CI_{i,k,t} * MSCM_{i,k,t}) * ESSD_{m,k,t} \quad (4)$$

where ESS demand depends on the battery lifetime m of ESS ($m \in \{10,20\}$).

3.2 Traditional end uses

The prospective cobalt demand for traditional end uses ($CoDemand_{tra}$) is estimated by the stock-driven model, and j indicates different categories of traditional end uses ($j = 1,2,3,4,5,6,7$). The prospective cobalt stock (S) of region regional k for traditional end uses are assumed driven by regional cobalt stock per capita by end uses (SPC) and population (P) of each region, which is then combined with the lifetime distribution function $pdf(\tau - T_T)$ and the average product lifetime (T_T). τ stands for the actual time of input in the model and T is the time step of the model. ΔS is the gap between the actual stock (S) and the remaining stock (S').

$$CoDemand_{tra}(j,k,t) = \Delta S(j,k,t)/T \quad (5)$$

$$\Delta S(j,k,t) = S(j,k,t) - S'(j,k,t) = S(j,k,t) - \int_{t_0}^{t} CoDemand'_{tra}(j,k,\tau)d\tau - \int_{t_0}^{t}\int_{t_0}^{\tau} CoDemand'_{tra}(j,k,\theta)\cdot pdf(\tau-\theta)d\theta \quad (6)$$

$$S(j,k,t) = P(j,k,t) * SPC(j,k,t) \quad (7)$$

References

1. Hao, H., Liu, Z., Zhao, F., Geng, Y. & Sarkis, J. Material flow analysis of lithium in China. *Resour. Policy* **51**, 100–106 (2017).
2. O'Donovan, A., Frith, J. & McKerracher, C. *Electric Buses in Cities: Driving Towards Cleaner Air and Lower CO2*. https://assets.bbhub.io/professional/sites/24/2018/05/Electric-Buses-in-Cities-Report-BNEF-C40-Citi.pdf (2018).
3. Dunn, J. B., Gaines, L., Barnes, M., Sullivan, J. & Wang, M. *Material and energy flows in the materials production, assembly, and end-of-life stages of the automotive lithium-ion battery life cycle*. https://publications.anl.gov/anlpubs/2014/11/109509.pdf (2012).
4. Harper, E. M., Kavlak, G. & Graedel, T. E. Tracking the Metal of the Goblins: Cobalt's Cycle of Use. *Environ. Sci. Technol.* **46**, 1079–1086 (2012).
5. Olivetti, E. A., Ceder, G., Gaustad, G. G. & Fu, X. Lithium-Ion Battery Supply Chain Considerations: Analysis of Potential Bottlenecks in Critical Metals. *Joule* **1**, 229–243 (2017).
6. Richa, K., Babbitt, C. W., Gaustad, G. & Wang, X. A future perspective on lithium-ion battery waste flows from electric vehicles. *Resour. Conserv. Recycl.* **83**, 63–76 (2014).
7. Wang, X., Gaustad, G., Babbitt, C. W. & Richa, K. Economies of scale for future lithium-ion battery recycling infrastructure. *Resour. Conserv. Recycl.* **83**, 53–62 (2014).
8. Alves Dias, P., Blagoeva, D., Pavel, C. & Arvanitidis, N. *Cobalt: demand-supply balances in the transition to electric mobility*. https://data.europa.eu/doi/10.2760/97710 (2018).
9. Song, J. *et al.* Material flow analysis on critical raw materials of lithium-ion batteries in China. *J. Clean. Prod.* **215**, 570–581 (2019).

10. Wang, Y. & Ge, J. Potential of urban cobalt mines in China: An estimation of dynamic material flow from 2007 to 2016. *Resour. Conserv. Recycl.* **161**, 104955 (2020).

11. Menad, N. & Seron, A. *Characterization of permanent magnets from WEEE*. https://hal-brgm.archives-ouvertes.fr/hal-01298267/document (2016).

12. Cobalt Institute, Magnetic Alloys. https://www.cobaltinstitute.org/magnetic-alloys.html.

13. Cobalt processing: Uses, Supply, & Facts. *Encyclopedia Britannica* https://www.britannica.com/technology/cobalt-processing.

14. Cobalt Institute, Inks and Pigments. https://www.cobaltinstitute.org/inks-and-pigments.html.

15. *MCRDrier technical data sheet*. http://www.smartchem.com.ar/.

16. Graedel, T. E. *et al.* What Do We Know About Metal Recycling Rates? *J. Ind. Ecol.* **15**, 355–366 (2011).

17. Rasmussen, K. D., Wenzel, H., Bangs, C., Petavratzi, E. & Liu, G. Platinum Demand and Potential Bottlenecks in the Global Green Transition: A Dynamic Material Flow Analysis. *Environ. Sci. Technol.* **53**, 11541–11551 (2019).

18. Liu, G. & Müller, D. B. Centennial Evolution of Aluminum In-Use Stocks on Our Aluminized Planet. *Environ. Sci. Technol.* **47**, 4882–4888 (2013).

19. Japan Oil, Gas and Metals National Corporation, (JOGMEC). Mineral Resource Information. http://mric.jogmec.go.jp/.

20. China Non-Ferrous Metals Industry Association, (CNIA). Statistics of China nonferrous metals industry association of Cobalt. http://chinacobalt.org/.

21. U.S. Geological Survey, (USGS), Science for a changing world. https://www.usgs.gov/.

22. World mineral statistics | MineralsUK. https://www2.bgs.ac.uk/mineralsuk/statistics/worldStatistics.html.

23. UN Comtrade, International Trade Statistics Database. https://comtrade.un.org/.

24. Slavko, S., Petiot, C., Planchon, M., Trigo, A. de P. & Devauze, C. *Study on Data for a Raw Material System Analysis: Roadmap and Test of the Fully Operational MSA for Raw Materials*. https://ec.europa.eu/assets/jrc/msa/images/msa_final_report.pdf (2015).

25. Chen, Z., Zhang, L. & Xu, Z. Tracking and quantifying the cobalt flows in mainland China during 1994–2016: Insights into use, trade and prospective demand. *Sci. Total Environ.* **672**, 752–762 (2019).

26. Antaike personal communication. https://www.antaike.com/.

27. Berman, K. *et al. The Lithium Ion Battery and the EV Market The Science Behind What You Cant See*. https://iea.blob.core.windows.net/assets/imports/events/71/Session3ColinHamiltonBMO.pdf (2018).

28. Roland, I. & Pontes, J. EV Data Center. *EV-Volumes.com* http://www.ev-volumes.com/datacenter/ (2019).

29. Lyon, P. Tesla Might Be World’s Most Valuable Automaker But Japan Didn’t Get Memo. *Forbes* https://www.forbes.com/sites/peterlyon/2020/07/29/tesla-might-be-worlds-most-valuable-automaker-but-japan-didnt-get-memo/.

30. Azevedo, M. *et al. Lithium and cobalt – a tale of two commodities*. https://www.mckinsey.com/~/media/mckinsey/industries/metals%20and%20mining/our%20insights/lithium%20and%20cobalt%20a%20tale%20of%20two%20commodities/lithium-and-cobalt-a-tale-of-two-commodities.pdf (2018).
31. Energy Storage Service | Wood Mackenzie. https://www.woodmac.com/industry/power-and-renewables/energy-storage-service/.
32. Vandeputte, K. *Increasing the gap in Rechargeable Battery Materials*. https://www.umicore.com/storage/group/powering-ahead-kurt-vandeputte.pdf (2018).
33. International Energy Agency, (IEA). *World Energy Outlook 2018*. https://iea.blob.core.windows.net/assets/77ecf96c-5f4b-4d0d-9d93-d81b938217cb/World_Energy_Outlook_2018.pdf (2018).
34. Graedel, T. E., Harper, E. M., Nassar, N. T., Nuss, P. & Reck, B. K. Criticality of metals and metalloids. *PNAS* **112**, 4257–4262 (2015).
35. Shedd, K. B. *Cobalt recycling in the United States in 1998*. (2002).
36. Dehaine, Q., Tijsseling, L. T., Glass, H. J., Törmänen, T. & Butcher, A. R. Geometallurgy of cobalt ores: A review. *Miner. Eng.* **160**, 106656 (2021).
37. Schmidt, T., Buchert, M. & Schebek, L. Investigation of the primary production routes of nickel and cobalt products used for Li-ion batteries. *Resour. Conserv. Recycl.* **112**, 107–122 (2016).
38. Zablocki, A. *Fact Sheet: Energy Storage*. https://www.eesi.org/files/FactSheet_Energy_Storage_0219.pdf (2019).

39. Zeng, X. & Li, J. On the sustainability of cobalt utilization in China. *Resour. Conserv. Recycl.* **104**, 12–18 (2015).

40. Baars, J., Domenech, T., Bleischwitz, R., Melin, H. E. & Heidrich, O. Circular economy strategies for electric vehicle batteries reduce reliance on raw materials. *Nat. Sustain.* (2020).

41. Pillot, C. *The Rechargeable Battery Market and Main Trends 2011-2020*. https://niobium.tech/-/media/NiobiumTech/Documentos/2019-Formula-E---Berlin/NT_The-rechargeable-battery-market-and-main-trends.pdf (2016).

42. Hao, H., Cheng, X., Liu, Z. & Zhao, F. China's traction battery technology roadmap: Targets, impacts and concerns. *Energy Policy* **108**, 355–358 (2017).

43. Chen, R., Zhao, T., Zhang, X., Li, L. & Wu, F. Advanced cathode materials for lithium-ion batteries using nanoarchitectonics. *Nanoscale Horiz.* **1**, 423–444 (2016).

44. Lee, S.-H., Lee, S., Jin, B.-S. & Kim, H.-S. Optimized electrochemical performance of Ni rich LiNi0.91Co0.06Mn0.03O2 cathodes for high-energy lithium ion batteries. *Sci. Rep.* **9**, 8901 (2019).

45. Wentker, M., Greenwood, M. & Leker, J. A Bottom-Up Approach to Lithium-Ion Battery Cost Modeling with a Focus on Cathode Active Materials. *Energies* **12**, 504 (2019).

46. Li, W., Erickson, E. M. & Manthiram, A. High-nickel layered oxide cathodes for lithium-based automotive batteries. *Nat. Energy* **5**, 26–34 (2020).

47. Dunn, J., Slattery, M., Kendall, A., Ambrose, H. & Shen, S. Circularity of Lithium-Ion Battery Materials in Electric Vehicles. *Environ. Sci. Technol.* **55**, 5189–5198 (2021).

48. Ely, T. O., Kamzabek, D., Chakraborty, D. & Doherty, M. F. Lithium–Sulfur Batteries: State of the Art and Future Directions. *ACS Appl. Energy Mater.* **1**, 1783–1814 (2018).

49. Kato, Y. *et al.* High-power all-solid-state batteries using sulfide superionic conductors. *Nat. Energy* **1**, 16030 (2016).

50. Simon, B., Ziemann, S. & Weil, M. Potential metal requirement of active materials in lithium-ion battery cells of electric vehicles and its impact on reserves: Focus on Europe. *Resour. Conserv. Recycl.* **104**, 300–310 (2015).

51. Zhao, Q., Liu, X., Stalin, S., Khan, K. & Archer, L. A. Solid-state polymer electrolytes with in-built fast interfacial transport for secondary lithium batteries. *Nat. Energy* **4**, 365–373 (2019).

52. Pang, Q., Liang, X., Kwok, C. Y. & Nazar, L. F. Advances in lithium–sulfur batteries based on multifunctional cathodes and electrolytes. *Nat. Energy* **1**, 16132 (2016).

53. Jia, H. *et al.* Toward the Practical Use of Cobalt-Free Lithium-Ion Batteries by an Advanced Ether-Based Electrolyte. *ACS Appl. Mater. Interfaces* **13**, 44339–44347 (2021).

54. Louli, A. J. *et al.* Diagnosing and correcting anode-free cell failure via electrolyte and morphological analysis. *Nat. Energy* **5**, 693–702 (2020).

55. Yu, Z. *et al.* Molecular design for electrolyte solvents enabling energy-dense and long-cycling lithium metal batteries. *Nat. Energy* **5**, 526–533 (2020).

56. International Energy Agency, (IEA). *Global EV Outlook 2019: Scaling-up the transition to electric mobility*. https://www.oecd-ilibrary.org/energy/global-ev-outlook-2019_35fb60bd-en (2019).

57. Global Storage Market to Double Six Times by 2030. *BloombergNEF* https://about.bnef.com/blog/global-storage-market-double-six-times-2030/ (2017).

58. Nansai, K. *et al.* Global Flows of Critical Metals Necessary for Low-Carbon Technologies: The Case of Neodymium, Cobalt, and Platinum. *Environ. Sci. Technol.* **48**, 1391–1400 (2014).

59. Knobloch, V., Zimmermann, T. & Gößling-Reisemann, S. From criticality to vulnerability of resource supply: The case of the automobile industry. *Resour. Conserv. Recycl.* **138**, 272–282 (2018).

60. Liu, G. & Müller, D. B. Mapping the Global Journey of Anthropogenic Aluminum: A Trade-Linked Multilevel Material Flow Analysis. *Environ. Sci. Technol.* **47**, 11873–11881 (2013).

61. Cao, Z., Shen, L., Løvik, A. N., Müller, D. B. & Liu, G. Elaborating the History of Our Cementing Societies: An in-Use Stock Perspective. *Environ. Sci. Technol.* **51**, 11468–11475 (2017).

62. Pauliuk, S., Wang, T. & Müller, D. B. Steel all over the world: Estimating in-use stocks of iron for 200 countries. *Resour. Conserv. Recycl.* **71**, 22–30 (2013).

63. B. Müller, D. Stock dynamics for forecasting material flows—Case study for housing in The Netherlands. *Ecol. Econ.* **59**, 142–156 (2006).

64. Liu, G., Bangs, C. E. & Müller, D. B. Stock dynamics and emission pathways of the global aluminium cycle. *Nat. Clim. Change* **3**, 338–342 (2013).

65. Pauliuk, S., Dhaniati, N. M. A. & Müller, D. B. Reconciling Sectoral Abatement Strategies with Global Climate Targets: The Case of the Chinese Passenger Vehicle Fleet. *Environ. Sci. Technol.* **46**, 140–147 (2012).
